# Highly accurate protein structure prediction with AlphaFold

**DOI:** 10.1038/s41586-021-03819-2

**Published:** 2021-07-15

**Authors:** John Jumper, Richard Evans, Alexander Pritzel, Tim Green, Michael Figurnov, Olaf Ronneberger, Kathryn Tunyasuvunakool, Russ Bates, Augustin Žídek, Anna Potapenko, Alex Bridgland, Clemens Meyer, Simon A. A. Kohl, Andrew J. Ballard, Andrew Cowie, Bernardino Romera-Paredes, Stanislav Nikolov, Rishub Jain, Jonas Adler, Trevor Back, Stig Petersen, David Reiman, Ellen Clancy, Michal Zielinski, Martin Steinegger, Michalina Pacholska, Tamas Berghammer, Sebastian Bodenstein, David Silver, Oriol Vinyals, Andrew W. Senior, Koray Kavukcuoglu, Pushmeet Kohli, Demis Hassabis

**Affiliations:** 1 DeepMind, London, UK; 2 School of Biological Sciences, Seoul National University, Seoul, South Korea; 3 Artificial Intelligence Institute, Seoul National University, Seoul, South Korea

**Keywords:** Computational biophysics, Machine learning, Protein structure predictions, Structural biology

## Abstract

Proteins are essential to life, and understanding their structure can facilitate a mechanistic understanding of their function. Through an enormous experimental effort^1–4^, the structures of around 100,000 unique proteins have been determined^5^, but this represents a small fraction of the billions of known protein sequences^6,7^. Structural coverage is bottlenecked by the months to years of painstaking effort required to determine a single protein structure. Accurate computational approaches are needed to address this gap and to enable large-scale structural bioinformatics. Predicting the three-dimensional structure that a protein will adopt based solely on its amino acid sequence—the structure prediction component of the ‘protein folding problem’^8^—has been an important open research problem for more than 50 years^9^. Despite recent progress^10–14^, existing methods fall far short of atomic accuracy, especially when no homologous structure is available. Here we provide the first computational method that can regularly predict protein structures with atomic accuracy even in cases in which no similar structure is known. We validated an entirely redesigned version of our neural network-based model, AlphaFold, in the challenging 14th Critical Assessment of protein Structure Prediction (CASP14)^15^, demonstrating accuracy competitive with experimental structures in a majority of cases and greatly outperforming other methods. Underpinning the latest version of AlphaFold is a novel machine learning approach that incorporates physical and biological knowledge about protein structure, leveraging multi-sequence alignments, into the design of the deep learning algorithm.

## Abstract (web summary)

AlphaFold predicts protein structures with an accuracy competitive with experimental structures in the majority of cases using a novel deep learning architecture.

## Main

The development of computational methods to predict three-dimensional (3D) protein structures from the protein sequence has proceeded along two complementary paths that focus on either the physical interactions or the evolutionary history. The physical interaction programme heavily integrates our understanding of molecular driving forces into either thermodynamic or kinetic simulation of protein physics^16^ or statistical approximations thereof^17^. Although theoretically very appealing, this approach has proved highly challenging for even moderate-sized proteins due to the computational intractability of molecular simulation, the context dependence of protein stability and the difficulty of producing sufficiently accurate models of protein physics. The evolutionary programme has provided an alternative in recent years, in which the constraints on protein structure are derived from bioinformatics analysis of the evolutionary history of proteins, homology to solved structures^18,19^ and pairwise evolutionary correlations^20–24^. This bioinformatics approach has benefited greatly from the steady growth of experimental protein structures deposited in the Protein Data Bank (PDB)^5^, the explosion of genomic sequencing and the rapid development of deep learning techniques to interpret these correlations. Despite these advances, contemporary physical and evolutionary-history-based approaches produce predictions that are far short of experimental accuracy in the majority of cases in which a close homologue has not been solved experimentally and this has limited their utility for many biological applications.

In this study, we develop the first, to our knowledge, computational approach capable of predicting protein structures to near experimental accuracy in a majority of cases. The neural network AlphaFold that we developed was entered into the CASP14 assessment (May–July 2020; entered under the team name ‘AlphaFold2’ and a completely different model from our CASP13 AlphaFold system^10^). The CASP assessment is carried out biennially using recently solved structures that have not been deposited in the PDB or publicly disclosed so that it is a blind test for the participating methods, and has long served as the gold-standard assessment for the accuracy of structure prediction^25,26^.

**Fig. 1:**
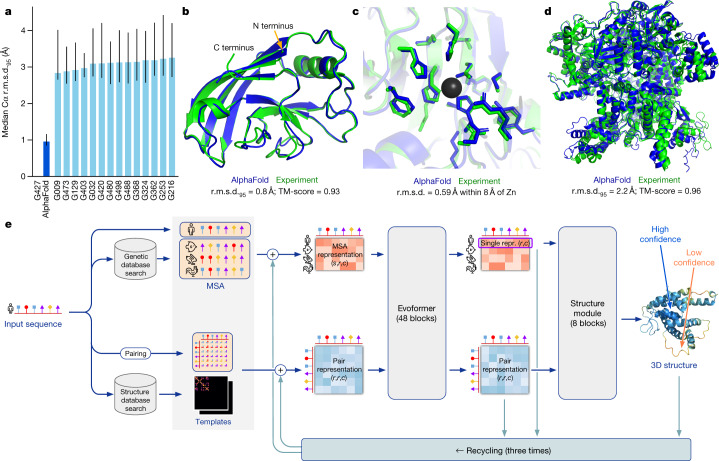
AlphaFold produces highly accurate structures. **a**, The performance of AlphaFold on the CASP14 dataset (*n* = 87 protein domains) relative to the top-15 entries (out of 146 entries), group numbers correspond to the numbers assigned to entrants by CASP. Data are median and the 95% confidence interval of the median, estimated from 10,000 bootstrap samples. **b**, Our prediction of CASP14 target T1049 (PDB 6Y4F, blue) compared with the true (experimental) structure (green). Four residues in the C terminus of the crystal structure are *B*-factor outliers and are not depicted. **c**, CASP14 target T1056 (PDB 6YJ1). An example of a well-predicted zinc-binding site (AlphaFold has accurate side chains even though it does not explicitly predict the zinc ion). **d**, CASP target T1044 (PDB 6VR4)—a 2,180-residue single chain—was predicted with correct domain packing (the prediction was made after CASP using AlphaFold without intervention). **e**, Model architecture. Arrows show the information flow among the various components described in this paper. Array shapes are shown in parentheses with *s*, number of sequences (*N*_seq_ in the main text); *r*, number of residues (*N*_res_ in the main text); *c*, number of channels.

In CASP14, AlphaFold structures were vastly more accurate than competing methods. AlphaFold structures had a median backbone accuracy of 0.96 Å r.m.s.d._95_ (Cα root-mean-square deviation at 95% residue coverage) (95% confidence interval = 0.85–1.16 Å) whereas the next best performing method had a median backbone accuracy of 2.8 Å r.m.s.d._95_ (95% confidence interval = 2.7–4.0 Å) (measured on CASP domains; see Fig. 1a for backbone accuracy and Supplementary Fig. 14 for all-atom accuracy). As a comparison point for this accuracy, the width of a carbon atom is approximately 1.4 Å. In addition to very accurate domain structures (Fig. 1b), AlphaFold is able to produce highly accurate side chains (Fig. 1c) when the backbone is highly accurate and considerably improves over template-based methods even when strong templates are available. The all-atom accuracy of AlphaFold was 1.5 Å r.m.s.d._95_ (95% confidence interval = 1.2–1.6 Å) compared with the 3.5 Å r.m.s.d._95_ (95% confidence interval = 3.1–4.2 Å) of the best alternative method. Our methods are scalable to very long proteins with accurate domains and domain-packing (see Fig. 1d for the prediction of a 2,180-residue protein with no structural homologues). Finally, the model is able to provide precise, per-residue estimates of its reliability that should enable the confident use of these predictions.

**Fig. 2:**
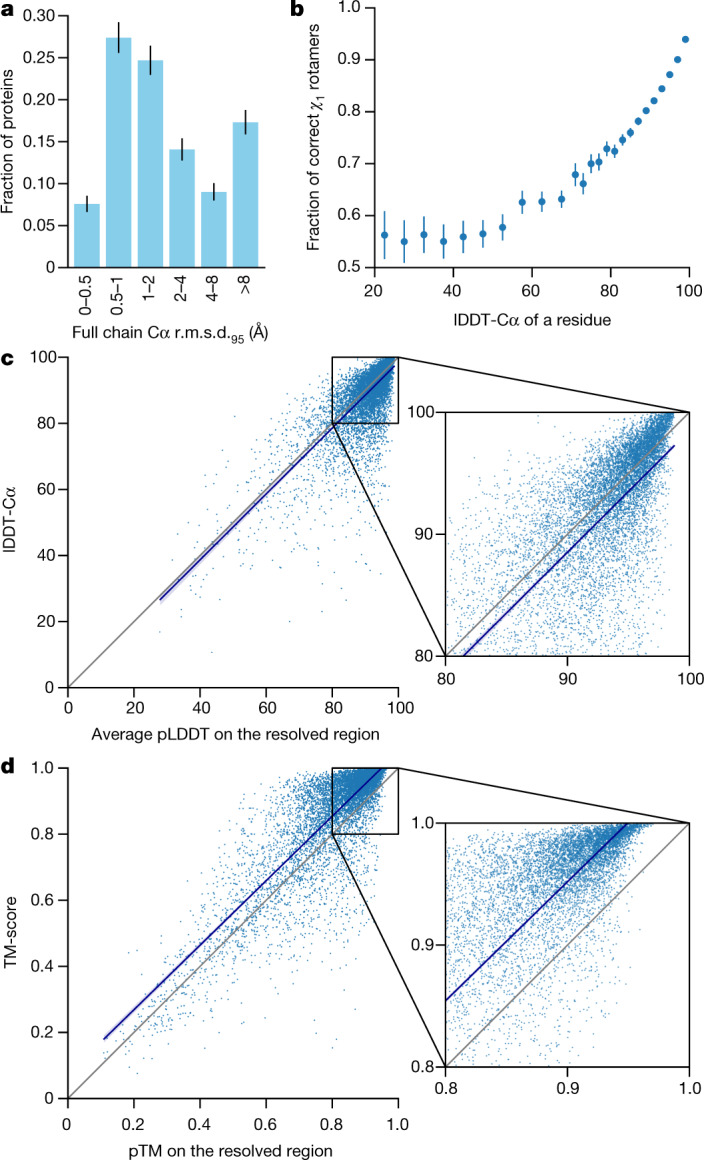
Accuracy of AlphaFold on recent PDB structures. The analysed structures are newer than any structure in the training set. Further filtering is applied to reduce redundancy (see Methods). **a**, Histogram of backbone r.m.s.d. for full chains (Cα r.m.s.d. at 95% coverage). Error bars are 95% confidence intervals (Poisson). This dataset excludes proteins with a template (identified by hmmsearch) from the training set with more than 40% sequence identity covering more than 1% of the chain (*n* = 3,144 protein chains). The overall median is 1.46 Å (95% confidence interval = 1.40–1.56 Å). Note that this measure will be highly sensitive to domain packing and domain accuracy; a high r.m.s.d. is expected for some chains with uncertain packing or packing errors. **b**, Correlation between backbone accuracy and side-chain accuracy. Filtered to structures with any observed side chains and resolution better than 2.5 Å (*n* = 5,317 protein chains); side chains were further filtered to *B*-factor <30 Å^2^. A rotamer is classified as correct if the predicted torsion angle is within 40°. Each point aggregates a range of lDDT-Cα, with a bin size of 2 units above 70 lDDT-Cα and 5 units otherwise. Points correspond to the mean accuracy; error bars are 95% confidence intervals (Student *t*-test) of the mean on a per-residue basis. **c**, Confidence score compared to the true accuracy on chains. Least-squares linear fit lDDT-Cα = 0.997 × pLDDT − 1.17 (Pearson’s *r* = 0.76). *n* = 10,795 protein chains. The shaded region of the linear fit represents a 95% confidence interval estimated from 10,000 bootstrap samples. In the companion paper^39^, additional quantification of the reliability of pLDDT as a confidence measure is provided. **d**, Correlation between pTM and full chain TM-score. Least-squares linear fit TM-score = 0.98 × pTM + 0.07 (Pearson’s *r* = 0.85). *n* = 10,795 protein chains. The shaded region of the linear fit represents a 95% confidence interval estimated from 10,000 bootstrap samples.

We demonstrate in Fig. 2a that the high accuracy that AlphaFold demonstrated in CASP14 extends to a large sample of recently released PDB structures; in this dataset, all structures were deposited in the PDB after our training data cut-off and are analysed as full chains (see Methods, Supplementary Fig. 15 and Supplementary Table 6 for more details). Furthermore, we observe high side-chain accuracy when the backbone prediction is accurate (Fig. 2b) and we show that our confidence measure, the predicted local-distance difference test (pLDDT), reliably predicts the Cα local-distance difference test (lDDT-Cα) accuracy of the corresponding prediction (Fig. 2c). We also find that the global superposition metric template modelling score (TM-score)^27^ can be accurately estimated (Fig. 2d). Overall, these analyses validate that the high accuracy and reliability of AlphaFold on CASP14 proteins also transfers to an uncurated collection of recent PDB submissions, as would be expected (see Supplementary Methods 1.15 and Supplementary Fig. 11 for confirmation that this high accuracy extends to new folds).

## The AlphaFold network

AlphaFold greatly improves the accuracy of structure prediction by incorporating novel neural network architectures and training procedures based on the evolutionary, physical and geometric constraints of protein structures. In particular, we demonstrate a new architecture to jointly embed multiple sequence alignments (MSAs) and pairwise features, a new output representation and associated loss that enable accurate end-to-end structure prediction, a new equivariant attention architecture, use of intermediate losses to achieve iterative refinement of predictions, masked MSA loss to jointly train with the structure, learning from unlabelled protein sequences using self-distillation and self-estimates of accuracy.

The AlphaFold network directly predicts the 3D coordinates of all heavy atoms for a given protein using the primary amino acid sequence and aligned sequences of homologues as inputs (Fig. 1e; see Methods for details of inputs including databases, MSA construction and use of templates). A description of the most important ideas and components is provided below. The full network architecture and training procedure are provided in the Supplementary Methods.

The network comprises two main stages. First, the trunk of the network processes the inputs through repeated layers of a novel neural network block that we term Evoformer to produce an *N*_seq_ × *N*_res_ array (*N*_seq_, number of sequences; *N*_res_, number of residues) that represents a processed MSA and an *N*_res_ × *N*_res_ array that represents residue pairs. The MSA representation is initialized with the raw MSA (although see Supplementary Methods 1.2.7 for details of handling very deep MSAs). The Evoformer blocks contain a number of attention-based and non-attention-based components. We show evidence in ‘Interpreting the neural network’ that a concrete structural hypothesis arises early within the Evoformer blocks and is continuously refined. The key innovations in the Evoformer block are new mechanisms to exchange information within the MSA and pair representations that enable direct reasoning about the spatial and evolutionary relationships.

The trunk of the network is followed by the structure module that introduces an explicit 3D structure in the form of a rotation and translation for each residue of the protein (global rigid body frames). These representations are initialized in a trivial state with all rotations set to the identity and all positions set to the origin, but rapidly develop and refine a highly accurate protein structure with precise atomic details. Key innovations in this section of the network include breaking the chain structure to allow simultaneous local refinement of all parts of the structure, a novel equivariant transformer to allow the network to implicitly reason about the unrepresented side-chain atoms and a loss term that places substantial weight on the orientational correctness of the residues. Both within the structure module and throughout the whole network, we reinforce the notion of iterative refinement by repeatedly applying the final loss to outputs and then feeding the outputs recursively into the same modules. The iterative refinement using the whole network (which we term ‘recycling’ and is related to approaches in computer vision^28,29^) contributes markedly to accuracy with minor extra training time (see Supplementary Methods 1.8 for details).

## Evoformer

**Fig. 3:**
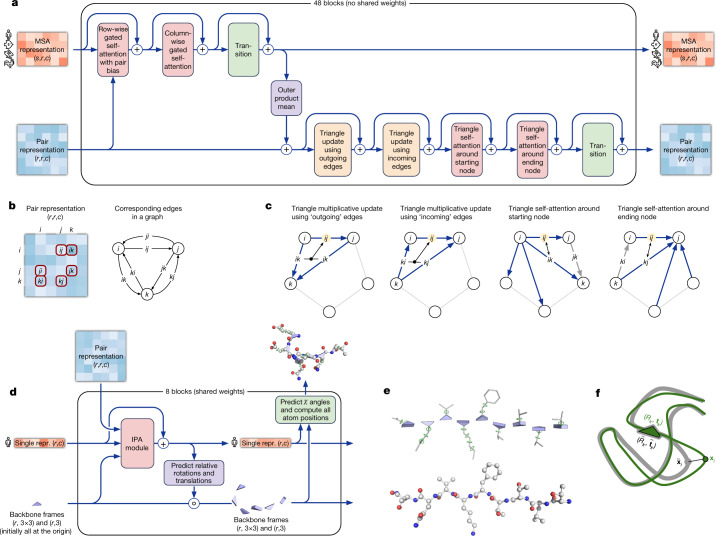
Architectural details. **a**, Evoformer block. Arrows show the information flow. The shape of the arrays is shown in parentheses. **b**, The pair representation interpreted as directed edges in a graph. **c**, Triangle multiplicative update and triangle self-attention. The circles represent residues. Entries in the pair representation are illustrated as directed edges and in each diagram, the edge being updated is *ij*. **d**, Structure module including Invariant point attention (IPA) module. The single representation is a copy of the first row of the MSA representation. **e**, Residue gas: a representation of each residue as one free-floating rigid body for the backbone (blue triangles) and *χ* angles for the side chains (green circles). The corresponding atomic structure is shown below. **f**, Frame aligned point error (FAPE). Green, predicted structure; grey, true structure; (*R*_*k*,_ **t**_*k*_), frames; **x**_i_, atom positions.

The key principle of the building block of the network—named Evoformer (Figs. 1e, 3a)—is to view the prediction of protein structures as a graph inference problem in 3D space in which the edges of the graph are defined by residues in proximity. The elements of the pair representation encode information about the relation between the residues (Fig. 3b). The columns of the MSA representation encode the individual residues of the input sequence while the rows represent the sequences in which those residues appear. Within this framework, we define a number of update operations that are applied in each block in which the different update operations are applied in series.

The MSA representation updates the pair representation through an element-wise outer product that is summed over the MSA sequence dimension. In contrast to previous work^30^, this operation is applied within every block rather than once in the network, which enables the continuous communication from the evolving MSA representation to the pair representation.

Within the pair representation, there are two different update patterns. Both are inspired by the necessity of consistency of the pair representation—for a pairwise description of amino acids to be representable as a single 3D structure, many constraints must be satisfied including the triangle inequality on distances. On the basis of this intuition, we arrange the update operations on the pair representation in terms of triangles of edges involving three different nodes (Fig. 3c). In particular, we add an extra logit bias to axial attention^31^ to include the ‘missing edge’ of the triangle and we define a non-attention update operation ‘triangle multiplicative update’ that uses two edges to update the missing third edge (see Supplementary Methods 1.6.5 for details). The triangle multiplicative update was developed originally as a more symmetric and cheaper replacement for the attention, and networks that use only the attention or multiplicative update are both able to produce high-accuracy structures. However, the combination of the two updates is more accurate.

We also use a variant of axial attention within the MSA representation. During the per-sequence attention in the MSA, we project additional logits from the pair stack to bias the MSA attention. This closes the loop by providing information flow from the pair representation back into the MSA representation, ensuring that the overall Evoformer block is able to fully mix information between the pair and MSA representations and prepare for structure generation within the structure module.

## End-to-end structure prediction

The structure module (Fig. 3d) operates on a concrete 3D backbone structure using the pair representation and the original sequence row (single representation) of the MSA representation from the trunk. The 3D backbone structure is represented as *N*_res_ independent rotations and translations, each with respect to the global frame (residue gas) (Fig. 3e). These rotations and translations—representing the geometry of the N-Cα-C atoms—prioritize the orientation of the protein backbone so that the location of the side chain of each residue is highly constrained within that frame. Conversely, the peptide bond geometry is completely unconstrained and the network is observed to frequently violate the chain constraint during the application of the structure module as breaking this constraint enables the local refinement of all parts of the chain without solving complex loop closure problems. Satisfaction of the peptide bond geometry is encouraged during fine-tuning by a violation loss term. Exact enforcement of peptide bond geometry is only achieved in the post-prediction relaxation of the structure by gradient descent in the Amber^32^ force field. Empirically, this final relaxation does not improve the accuracy of the model as measured by the global distance test (GDT)^33^ or lDDT-Cα^34^ but does remove distracting stereochemical violations without the loss of accuracy.

The residue gas representation is updated iteratively in two stages (Fig. 3d). First, a geometry-aware attention operation that we term ‘invariant point attention’ (IPA) is used to update an *N*_res_ set of neural activations (single representation) without changing the 3D positions, then an equivariant update operation is performed on the residue gas using the updated activations. The IPA augments each of the usual attention queries, keys and values with 3D points that are produced in the local frame of each residue such that the final value is invariant to global rotations and translations (see Methods ‘IPA’ for details). The 3D queries and keys also impose a strong spatial/locality bias on the attention, which is well-suited to the iterative refinement of the protein structure. After each attention operation and element-wise transition block, the module computes an update to the rotation and translation of each backbone frame. The application of these updates within the local frame of each residue makes the overall attention and update block an equivariant operation on the residue gas.

Predictions of side-chain *χ* angles as well as the final, per-residue accuracy of the structure (pLDDT) are computed with small per-residue networks on the final activations at the end of the network. The estimate of the TM-score (pTM) is obtained from a pairwise error prediction that is computed as a linear projection from the final pair representation. The final loss (which we term the frame-aligned point error (FAPE) (Fig. 3f)) compares the predicted atom positions to the true positions under many different alignments. For each alignment, defined by aligning the predicted frame (*R*_*k*_, **t**_*k*_) to the corresponding true frame, we compute the distance of all predicted atom positions **x**_*i*_ from the true atom positions. The resulting *N*_frames_ × *N*_atoms_ distances are penalized with a clamped *L*^1^ loss. This creates a strong bias for atoms to be correct relative to the local frame of each residue and hence correct with respect to its side-chain interactions, as well as providing the main source of chirality for AlphaFold (Supplementary Methods 1.9.3 and Supplementary Fig. 9).

## Training with labelled and unlabelled data

**Fig. 4:**
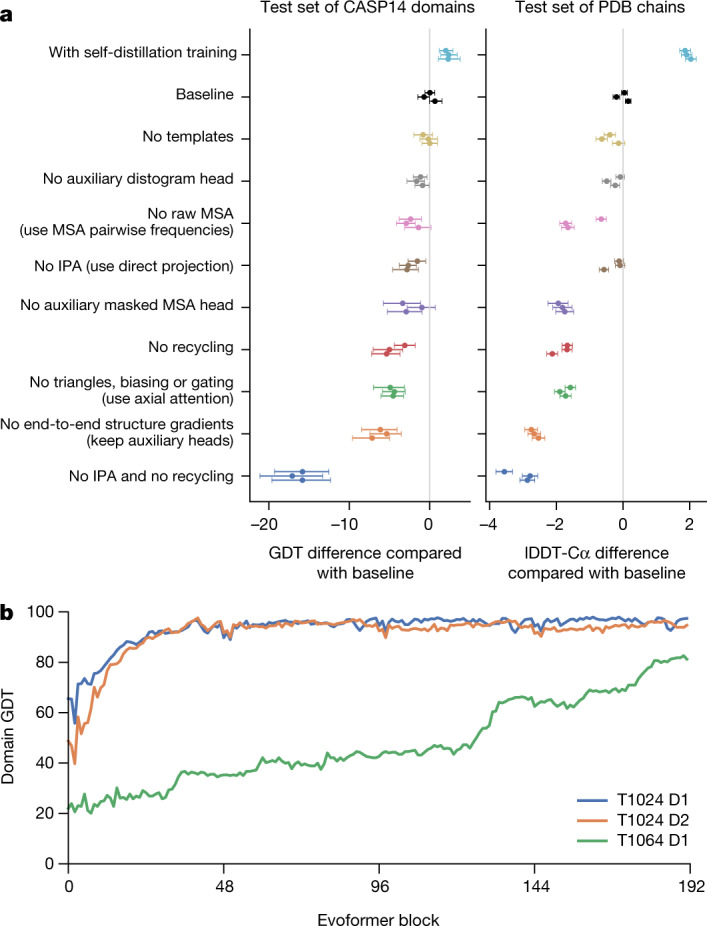
Interpreting the neural network. **a**, Ablation results on two target sets: the CASP14 set of domains (*n* = 87 protein domains) and the PDB test set of chains with template coverage of ≤30% at 30% identity (*n* = 2,261 protein chains). Domains are scored with GDT and chains are scored with lDDT-Cα. The ablations are reported as a difference compared with the average of the three baseline seeds. Means (points) and 95% bootstrap percentile intervals (error bars) are computed using bootstrap estimates of 10,000 samples. **b**, Domain GDT trajectory over 4 recycling iterations and 48 Evoformer blocks on CASP14 targets LmrP (T1024) and Orf8 (T1064) where D1 and D2 refer to the individual domains as defined by the CASP assessment. Both T1024 domains obtain the correct structure early in the network, whereas the structure of T1064 changes multiple times and requires nearly the full depth of the network to reach the final structure. Note, 48 Evoformer blocks comprise one recycling iteration.

The AlphaFold architecture is able to train to high accuracy using only supervised learning on PDB data, but we are able to enhance accuracy (Fig. 4a) using an approach similar to noisy student self-distillation^35^. In this procedure, we use a trained network to predict the structure of around 350,000 diverse sequences from Uniclust30^36^ and make a new dataset of predicted structures filtered to a high-confidence subset. We then train the same architecture again from scratch using a mixture of PDB data and this new dataset of predicted structures as the training data, in which the various training data augmentations such as cropping and MSA subsampling make it challenging for the network to recapitulate the previously predicted structures. This self-distillation procedure makes effective use of the unlabelled sequence data and considerably improves the accuracy of the resulting network.

Additionally, we randomly mask out or mutate individual residues within the MSA and have a Bidirectional Encoder Representations from Transformers (BERT)-style^37^ objective to predict the masked elements of the MSA sequences. This objective encourages the network to learn to interpret phylogenetic and covariation relationships without hardcoding a particular correlation statistic into the features. The BERT objective is trained jointly with the normal PDB structure loss on the same training examples and is not pre-trained, in contrast to recent independent work^38^.

## Interpreting the neural network

To understand how AlphaFold predicts protein structure, we trained a separate structure module for each of the 48 Evoformer blocks in the network while keeping all parameters of the main network frozen (Supplementary Methods 1.14). Including our recycling stages, this provides a trajectory of 192 intermediate structures—one per full Evoformer block—in which each intermediate represents the belief of the network of the most likely structure at that block. The resulting trajectories are surprisingly smooth after the first few blocks, showing that AlphaFold makes constant incremental improvements to the structure until it can no longer improve (see Fig. 4b for a trajectory of accuracy). These trajectories also illustrate the role of network depth. For very challenging proteins such as ORF8 of SARS-CoV-2 (T1064), the network searches and rearranges secondary structure elements for many layers before settling on a good structure. For other proteins such as LmrP (T1024), the network finds the final structure within the first few layers. Structure trajectories of CASP14 targets T1024, T1044, T1064 and T1091 that demonstrate a clear iterative building process for a range of protein sizes and difficulties are shown in Supplementary Videos 1–4. In Supplementary Methods 1.16 and Supplementary Figs. 12, 13, we interpret the attention maps produced by AlphaFold layers.

Figure 4a contains detailed ablations of the components of AlphaFold that demonstrate that a variety of different mechanisms contribute to AlphaFold accuracy. Detailed descriptions of each ablation model, their training details, extended discussion of ablation results and the effect of MSA depth on each ablation are provided in Supplementary Methods 1.13 and Supplementary Fig. 10.

## MSA depth and cross-chain contacts

**Fig. 5:**
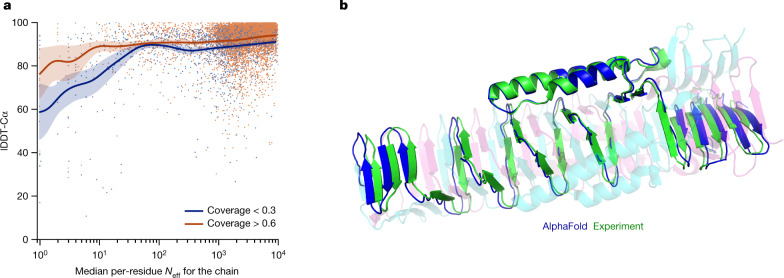
Effect of MSA depth and cross-chain contacts. **a**, Backbone accuracy (lDDT-Cα) for the redundancy-reduced set of the PDB after our training data cut-off, restricting to proteins in which at most 25% of the long-range contacts are between different heteromer chains. We further consider two groups of proteins based on template coverage at 30% sequence identity: covering more than 60% of the chain (*n* = 6,743 protein chains) and covering less than 30% of the chain (*n* = 1,596 protein chains). MSA depth is computed by counting the number of non-gap residues for each position in the MSA (using the *N*_eff_ weighting scheme; see Methods for details) and taking the median across residues. The curves are obtained through Gaussian kernel average smoothing (window size is 0.2 units in log_10_(*N*_eff_)); the shaded area is the 95% confidence interval estimated using bootstrap of 10,000 samples. **b**, An intertwined homotrimer (PDB 6SK0) is correctly predicted without input stoichiometry and only a weak template (blue is predicted and green is experimental).

Although AlphaFold has a high accuracy across the vast majority of deposited PDB structures, we note that there are still factors that affect accuracy or limit the applicability of the model. The model uses MSAs and the accuracy decreases substantially when the median alignment depth is less than around 30 sequences (see Fig. 5a for details). We observe a threshold effect where improvements in MSA depth over around 100 sequences lead to small gains. We hypothesize that the MSA information is needed to coarsely find the correct structure within the early stages of the network, but refinement of that prediction into a high-accuracy model does not depend crucially on the MSA information. The other substantial limitation that we have observed is that AlphaFold is much weaker for proteins that have few intra-chain or homotypic contacts compared to the number of heterotypic contacts (further details are provided in a companion paper^39^). This typically occurs for bridging domains within larger complexes in which the shape of the protein is created almost entirely by interactions with other chains in the complex. Conversely, AlphaFold is often able to give high-accuracy predictions for homomers, even when the chains are substantially intertwined (Fig. 5b). We expect that the ideas of AlphaFold are readily applicable to predicting full hetero-complexes in a future system and that this will remove the difficulty with protein chains that have a large number of hetero-contacts.

## Related work

The prediction of protein structures has had a long and varied development, which is extensively covered in a number of reviews^14,40–43^. Despite the long history of applying neural networks to structure prediction^14,42,43^, they have only recently come to improve structure prediction^10,11,44,45^. These approaches effectively leverage the rapid improvement in computer vision systems^46^ by treating the problem of protein structure prediction as converting an ‘image’ of evolutionary couplings^22–24^ to an ‘image’ of the protein distance matrix and then integrating the distance predictions into a heuristic system that produces the final 3D coordinate prediction. A few recent studies have been developed to predict the 3D coordinates directly^47–50^, but the accuracy of these approaches does not match traditional, hand-crafted structure prediction pipelines^51^. In parallel, the success of attention-based networks for language processing^52^ and, more recently, computer vision^31,53^ has inspired the exploration of attention-based methods for interpreting protein sequences^54–56^.

## Discussion

The methodology that we have taken in designing AlphaFold is a combination of the bioinformatics and physical approaches: we use a physical and geometric inductive bias to build components that learn from PDB data with minimal imposition of handcrafted features (for example, AlphaFold builds hydrogen bonds effectively without a hydrogen bond score function). This results in a network that learns far more efficiently from the limited data in the PDB but is able to cope with the complexity and variety of structural data.

In particular, AlphaFold is able to handle missing the physical context and produce accurate models in challenging cases such as intertwined homomers or proteins that only fold in the presence of an unknown haem group. The ability to handle underspecified structural conditions is essential to learning from PDB structures as the PDB represents the full range of conditions in which structures have been solved. In general, AlphaFold is trained to produce the protein structure most likely to appear as part of a PDB structure. For example, in cases in which a particular stochiometry, ligand or ion is predictable from the sequence alone, AlphaFold is likely to produce a structure that respects those constraints implicitly.

AlphaFold has already demonstrated its utility to the experimental community, both for molecular replacement^57^ and for interpreting cryogenic electron microscopy maps^58^. Moreover, because AlphaFold outputs protein coordinates directly, AlphaFold produces predictions in graphics processing unit (GPU) minutes to GPU hours depending on the length of the protein sequence (for example, around one GPU minute per model for 384 residues; see Methods for details). This opens up the exciting possibility of predicting structures at the proteome-scale and beyond—in a companion paper^39^, we demonstrate the application of AlphaFold to the entire human proteome^39^.

The explosion in available genomic sequencing techniques and data has revolutionized bioinformatics but the intrinsic challenge of experimental structure determination has prevented a similar expansion in our structural knowledge. By developing an accurate protein structure prediction algorithm, coupled with existing large and well-curated structure and sequence databases assembled by the experimental community, we hope to accelerate the advancement of structural bioinformatics that can keep pace with the genomics revolution. We hope that AlphaFold—and computational approaches that apply its techniques for other biophysical problems—will become essential tools of modern biology.

## Methods

### Full algorithm details

Extensive explanations of the components and their motivations are available in Supplementary Methods 1.1–1.10, in addition, pseudocode is available in Supplementary Information Algorithms 1–32, network diagrams in Supplementary Figs. 1–8, input features in Supplementary Table 1 and additional details are provided in Supplementary Tables 2, 3. Training and inference details are provided in Supplementary Methods 1.11–1.12 and Supplementary Tables 4, 5.

### IPA

The IPA module combines the pair representation, the single representation and the geometric representation to update the single representation (Supplementary Fig. 8). Each of these representations contributes affinities to the shared attention weights and then uses these weights to map its values to the output. The IPA operates in 3D space. Each residue produces query points, key points and value points in its local frame. These points are projected into the global frame using the backbone frame of the residue in which they interact with each other. The resulting points are then projected back into the local frame. The affinity computation in the 3D space uses squared distances and the coordinate transformations ensure the invariance of this module with respect to the global frame (see Supplementary Methods 1.8.2 ‘Invariant point attention (IPA)’ for the algorithm, proof of invariance and a description of the full multi-head version). A related construction that uses classic geometric invariants to construct pairwise features in place of the learned 3D points has been applied to protein design^59^.

In addition to the IPA, standard dot product attention is computed on the abstract single representation and a special attention on the pair representation. The pair representation augments both the logits and the values of the attention process, which is the primary way in which the pair representation controls the structure generation.

### Inputs and data sources

Inputs to the network are the primary sequence, sequences from evolutionarily related proteins in the form of a MSA created by standard tools including jackhmmer^60^ and HHBlits^61^, and 3D atom coordinates of a small number of homologous structures (templates) where available. For both the MSA and templates, the search processes are tuned for high recall; spurious matches will probably appear in the raw MSA but this matches the training condition of the network.

One of the sequence databases used, Big Fantastic Database (BFD), was custom-made and released publicly (see ‘Data availability’) and was used by several CASP teams. BFD is one of the largest publicly available collections of protein families. It consists of 65,983,866 families represented as MSAs and hidden Markov models (HMMs) covering 2,204,359,010 protein sequences from reference databases, metagenomes and metatranscriptomes.

BFD was built in three steps. First, 2,423,213,294 protein sequences were collected from UniProt (Swiss-Prot&TrEMBL, 2017-11)^62^, a soil reference protein catalogue and the marine eukaryotic reference catalogue^7^, and clustered to 30% sequence identity, while enforcing a 90% alignment coverage of the shorter sequences using MMseqs2/Linclust^63^. This resulted in 345,159,030 clusters. For computational efficiency, we removed all clusters with less than three members, resulting in 61,083,719 clusters. Second, we added 166,510,624 representative protein sequences from Metaclust NR (2017-05; discarding all sequences shorter than 150 residues)^63^ by aligning them against the cluster representatives using MMseqs2^64^. Sequences that fulfilled the sequence identity and coverage criteria were assigned to the best scoring cluster. The remaining 25,347,429 sequences that could not be assigned were clustered separately and added as new clusters, resulting in the final clustering. Third, for each of the clusters, we computed an MSA using FAMSA^65^ and computed the HMMs following the Uniclust HH-suite database protocol^36^.

The following versions of public datasets were used in this study. Our models were trained on a copy of the PDB^5^ downloaded on 28 August 2019. For finding template structures at prediction time, we used a copy of the PDB downloaded on 14 May 2020, and the PDB70^66^ clustering database downloaded on 13 May 2020. For MSA lookup at both training and prediction time, we used Uniref90^67^ v.2020_01, BFD, Uniclust30^36^ v.2018_08 and MGnify^6^ v.2018_12. For sequence distillation, we used Uniclust30^36^ v.2018_08 to construct a distillation structure dataset. Full details are provided in Supplementary Methods 1.2.

For MSA search on BFD + Uniclust30, and template search against PDB70, we used HHBlits^61^ and HHSearch^66^ from hh-suite v.3.0-beta.3 (version 14/07/2017). For MSA search on Uniref90 and clustered MGnify, we used jackhmmer from HMMER3^68^. For constrained relaxation of structures, we used OpenMM v.7.3.1^69^ with the Amber99sb force field^32^. For neural network construction, running and other analyses, we used TensorFlow^70^, Sonnet^71^, NumPy^72^, Python^73^ and Colab^74^.

To quantify the effect of the different sequence data sources, we re-ran the CASP14 proteins using the same models but varying how the MSA was constructed. Removing BFD reduced the mean accuracy by 0.4 GDT, removing Mgnify reduced the mean accuracy by 0.7 GDT, and removing both reduced the mean accuracy by 6.1 GDT. In each case, we found that most targets had very small changes in accuracy but a few outliers had very large (20+ GDT) differences. This is consistent with the results in Fig. 5a in which the depth of the MSA is relatively unimportant until it approaches a threshold value of around 30 sequences when the MSA size effects become quite large. We observe mostly overlapping effects between inclusion of BFD and Mgnify, but having at least one of these metagenomics databases is very important for target classes that are poorly represented in UniRef, and having both was necessary to achieve full CASP accuracy.

### Training regimen

To train, we use structures from the PDB with a maximum release date of 30 April 2018. Chains are sampled in inverse proportion to cluster size of a 40% sequence identity clustering. We then randomly crop them to 256 residues and assemble into batches of size 128. We train the model on Tensor Processing Unit (TPU) v3 with a batch size of 1 per TPU core, hence the model uses 128 TPU v3 cores. The model is trained until convergence (around 10 million samples) and further fine-tuned using longer crops of 384 residues, larger MSA stack and reduced learning rate (see Supplementary Methods 1.11 for the exact configuration). The initial training stage takes approximately 1 week, and the fine-tuning stage takes approximately 4 additional days.

The network is supervised by the FAPE loss and a number of auxiliary losses. First, the final pair representation is linearly projected to a binned distance distribution (distogram) prediction, scored with a cross-entropy loss. Second, we use random masking on the input MSAs and require the network to reconstruct the masked regions from the output MSA representation using a BERT-like loss^37^. Third, the output single representations of the structure module are used to predict binned per-residue lDDT-Cα values. Finally, we use an auxiliary side-chain loss during training, and an auxiliary structure violation loss during fine-tuning. Detailed descriptions and weighting are provided in the Supplementary Information.

An initial model trained with the above objectives was used to make structure predictions for a Uniclust dataset of 355,993 sequences with the full MSAs. These predictions were then used to train a final model with identical hyperparameters, except for sampling examples 75% of the time from the Uniclust prediction set, with sub-sampled MSAs, and 25% of the time from the clustered PDB set.

We train five different models using different random seeds, some with templates and some without, to encourage diversity in the predictions (see Supplementary Table 5 and Supplementary Methods 1.12.1 for details). We also fine-tuned these models after CASP14 to add a pTM prediction objective (Supplementary Methods 1.9.7) and use the obtained models for Fig. 2d.

### Inference regimen

We inference the five trained models and use the predicted confidence score to select the best model per target.

Using our CASP14 configuration for AlphaFold, the trunk of the network is run multiple times with different random choices for the MSA cluster centres (see Supplementary Methods 1.11.2 for details of the ensembling procedure). The full time to make a structure prediction varies considerably depending on the length of the protein. Representative timings for the neural network using a single model on V100 GPU are 4.8 min with 256 residues, 9.2 min with 384 residues and 18 h at 2,500 residues. These timings are measured using our open-source code, and the open-source code is notably faster than the version we ran in CASP14 as we now use the XLA compiler^75^.

Since CASP14, we have found that the accuracy of the network without ensembling is very close or equal to the accuracy with ensembling and we turn off ensembling for most inference. Without ensembling, the network is 8× faster and the representative timings for a single model are 0.6 min with 256 residues, 1.1 min with 384 residues and 2.1 h with 2,500 residues.

Inferencing large proteins can easily exceed the memory of a single GPU. For a V100 with 16 GB of memory, we can predict the structure of proteins up to around 1,300 residues without ensembling and the 256- and 384-residue inference times are using the memory of a single GPU. The memory usage is approximately quadratic in the number of residues, so a 2,500-residue protein involves using unified memory so that we can greatly exceed the memory of a single V100. In our cloud setup, a single V100 is used for computation on a 2,500-residue protein but we requested four GPUs to have sufficient memory.

Searching genetic sequence databases to prepare inputs and final relaxation of the structures take additional central processing unit (CPU) time but do not require a GPU or TPU.

### Metrics

The predicted structure is compared to the true structure from the PDB in terms of lDDT metric^34^, as this metric reports the domain accuracy without requiring a domain segmentation of chain structures. The distances are either computed between all heavy atoms (lDDT) or only the Cα atoms to measure the backbone accuracy (lDDT-Cα). As lDDT-Cα only focuses on the Cα atoms, it does not include the penalty for structural violations and clashes. Domain accuracies in CASP are reported as GDT^33^ and the TM-score^27^ is used as a full chain global superposition metric.

We also report accuracies using the r.m.s.d._95_ (Cα r.m.s.d. at 95% coverage). We perform five iterations of (1) a least-squares alignment of the predicted structure and the PDB structure on the currently chosen Cα atoms (using all Cα atoms in the first iteration); (2) selecting the 95% of Cα atoms with the lowest alignment error. The r.m.s.d. of the atoms chosen for the final iterations is the r.m.s.d._95_. This metric is more robust to apparent errors that can originate from crystal structure artefacts, although in some cases the removed 5% of residues will contain genuine modelling errors.

### Test set of recent PDB sequences

For evaluation on recent PDB sequences (Figs. 2a–d, 4a, 5a), we used a copy of the PDB downloaded 15 February 2021. Structures were filtered to those with a release date after 30 April 2018 (the date limit for inclusion in the training set for AlphaFold). Chains were further filtered to remove sequences that consisted of a single amino acid as well as sequences with an ambiguous chemical component at any residue position. Exact duplicates were removed, with the chain with the most resolved Cα atoms used as the representative sequence. Subsequently, structures with less than 16 resolved residues, with unknown residues or solved by NMR methods were removed. As the PDB contains many near-duplicate sequences, the chain with the highest resolution was selected from each cluster in the PDB 40% sequence clustering of the data. Furthermore, we removed all sequences for which fewer than 80 amino acids had the alpha carbon resolved and removed chains with more than 1,400 residues. The final dataset contained 10,795 protein sequences.

The procedure for filtering the recent PDB dataset based on prior template identity was as follows. Hmmsearch was run with default parameters against a copy of the PDB SEQRES fasta downloaded 15 February 2021. Template hits were accepted if the associated structure had a release date earlier than 30 April 2018. Each residue position in a query sequence was assigned the maximum identity of any template hit covering that position. Filtering then proceeded as described in the individual figure legends, based on a combination of maximum identity and sequence coverage.

The MSA depth analysis was based on computing the normalized number of effective sequences (*N*_eff_) for each position of a query sequence. Per-residue *N*_eff_ values were obtained by counting the number of non-gap residues in the MSA for this position and weighting the sequences using the *N*_eff_ scheme^76^ with a threshold of 80% sequence identity measured on the region that is non-gap in either sequence.

### Reporting summary

Further information on research design is available in the Nature Research Reporting Summary linked to this paper.

## Online content

Any methods, additional references, Nature Research reporting summaries, source data, extended data, supplementary information, acknowledgements, peer review information; details of author contributions and competing interests; and statements of data and code availability are available at 10.1038/s41586-021-03819-2.

## Supplementary information

Supplementary Information Description of the method details of the AlphaFold system, model, and analysis, including data pipeline, datasets, model blocks, loss functions, training and inference details, and ablations. Includes Supplementary Methods, Supplementary Figures, Supplementary Tables and Supplementary Algorithms.

Reporting Summary

Supplementary Video 1 Video of the intermediate structure trajectory of the CASP14 target T1024 (LmrP) A two-domain target (408 residues). Both domains are folded early, while their packing is adjusted for a longer time.

Supplementary Video 2 Video of the intermediate structure trajectory of the CASP14 target T1044 (RNA polymerase of crAss-like phage). A large protein (2180 residues), with multiple domains. Some domains are folded quickly, while others take a considerable amount of time to fold.

Supplementary Video 3 Video of the intermediate structure trajectory of the CASP14 target T1064 (Orf8). A very difficult single-domain target (106 residues) that takes the entire depth of the network to fold.

Supplementary Video 4 Video of the intermediate structure trajectory of the CASP14 target T1091. A multi-domain target (863 residues). Individual domains’ structure is determined early, while the domain packing evolves throughout the network. The network is exploring unphysical configurations throughout the process, resulting in long ‘strings’ in the visualization.

## Data Availability

All input data are freely available from public sources. Structures from the PDB were used for training and as templates (https://www.wwpdb.org/ftp/pdb-ftp-sites; for the associated sequence data and 40% sequence clustering see also https://ftp.wwpdb.org/pub/pdb/derived_data/ and https://cdn.rcsb.org/resources/sequence/clusters/bc-40.out). Training used a version of the PDB downloaded 28 August 2019, while the CASP14 template search used a version downloaded 14 May 2020. The template search also used the PDB70 database, downloaded 13 May 2020 (https://wwwuser.gwdg.de/~compbiol/data/hhsuite/databases/hhsuite_dbs/). We show experimental structures from the PDB with accession numbers 6Y4F^77^, 6YJ1^78^, 6VR4^79^, 6SK0^80^, 6FES^81^, 6W6W^82^, 6T1Z^83^ and 7JTL^84^. For MSA lookup at both the training and prediction time, we used UniRef90 v.2020_01 (https://ftp.ebi.ac.uk/pub/databases/uniprot/previous_releases/release-2020_01/uniref/), BFD (https://bfd.mmseqs.com), Uniclust30 v.2018_08 (https://wwwuser.gwdg.de/~compbiol/uniclust/2018_08/) and MGnify clusters v.2018_12 (https://ftp.ebi.ac.uk/pub/databases/metagenomics/peptide_database/2018_12/). Uniclust30 v.2018_08 was also used as input for constructing a distillation structure dataset.

## Code availability

Source code for the AlphaFold model, trained weights and inference script are available under an open-source license at https://github.com/deepmind/alphafold.

Neural networks were developed with TensorFlow v.1 (https://github.com/tensorflow/tensorflow), Sonnet v.1 (https://github.com/deepmind/sonnet), JAX v.0.1.69 (https://github.com/google/jax/) and Haiku v.0.0.4 (https://github.com/deepmind/dm-haiku). The XLA compiler is bundled with JAX and does not have a separate version number.

For MSA search on BFD+Uniclust30, and for template search against PDB70, we used HHBlits and HHSearch from hh-suite v.3.0-beta.3 release 14/07/2017 (https://github.com/soedinglab/hh-suite). For MSA search on UniRef90 and clustered MGnify, we used jackhmmer from HMMER v.3.3 (http://eddylab.org/software/hmmer/). For constrained relaxation of structures, we used OpenMM v.7.3.1 (https://github.com/openmm/openmm) with the Amber99sb force field.

Construction of BFD used MMseqs2 v.925AF (https://github.com/soedinglab/MMseqs2) and FAMSA v.1.2.5 (https://github.com/refresh-bio/FAMSA).

Data analysis used Python v.3.6 (https://www.python.org/), NumPy v.1.16.4 (https://github.com/numpy/numpy), SciPy v.1.2.1 (https://www.scipy.org/), seaborn v.0.11.1 (https://github.com/mwaskom/seaborn), Matplotlib v.3.3.4 (https://github.com/matplotlib/matplotlib), bokeh v.1.4.0 (https://github.com/bokeh/bokeh), pandas v.1.1.5 (https://github.com/pandas-dev/pandas), plotnine v.0.8.0 (https://github.com/has2k1/plotnine), statsmodels v.0.12.2 (https://github.com/statsmodels/statsmodels) and Colab (https://research.google.com/colaboratory). TM-align v.20190822 (https://zhanglab.dcmb.med.umich.edu/TM-align/) was used for computing TM-scores. Structure visualizations were created in Pymol v.2.3.0 (https://github.com/schrodinger/pymol-open-source).
