# Supplementary material for: Highly accurate protein structure prediction with AlphaFold
Source: Nature. 2021 Jul 15;596(7873):583–9. doi: 10.1038/s41586-021-03819-2 (PMC8371605; doi:10.1038/s41586-021-03819-2)
Supplement: Supplementary file 1 — Description of the method details of the AlphaFold system, model, and analysis, including data pipeline, datasets, model blocks, loss functions, training and inference details, and ablations. Includes Supplementary Methods, Supplementary Figures, Supplementary Tables and Supplementary Algorithms. [file 41586_2021_3819_MOESM1_ESM.pdf]

nature portfolio

https://doi.org/10.1038/s41586-021-03819-2

# Supplementary information

# Highly accurate protein structure prediction with AlphaFold

In the format provided by the authors and unedited

# Supplementary Information for: Highly accurate protein structure prediction with AlphaFold

John Jumper[1*+], Richard Evans[1*], Alexander Pritzel[1*], Tim Green[1*], Michael Figurnov[1*], Olaf Ronneberger[1*], Kathryn Tunyasuvunakool[1*], Russ Bates[1*], Augustin Žídek[1*], Anna Potapenko[1*], Alex Bridgland[1*], Clemens Meyer[1*], Simon A A Kohl[1*], Andrew J Ballard[1*], Andrew Cowie[1*], Bernardino Romera-Paredes[1*], Stanislav Nikolov[1*], Rishub Jain[1*], Jonas Adler[1], Trevor Back[1], Stig Petersen[1], David Reiman[1], Ellen Clancy[1], Michal Zielinski[1], Martin Steinegger[2], Michalina Pacholska[1], Tamas Berghammer[1], Sebastian Bodenstein[1], David Silver[1], Oriol Vinyals[1], Andrew W Senior[1], Koray Kavukcuoglu[1], Pushmeet Kohli[1], and Demis Hassabis[1*+]

[1]DeepMind, London, UK
[2]School of Biological Sciences and Artificial Intelligence Institute, Seoul National University, Seoul, South Korea
[*]These authors contributed equally
[+]Corresponding authors: John Jumper, Demis Hassabis

## Contents

**1 Supplementary Methods** . . . **4**
- 1.1 Notation . . . 4
- 1.2 Data pipeline . . . 5
  - 1.2.1 Parsing . . . 5
  - 1.2.2 Genetic search . . . 5
  - 1.2.3 Template search . . . 5
  - 1.2.4 Training data . . . 6
  - 1.2.5 Filtering . . . 6
  - 1.2.6 MSA block deletion . . . 6
  - 1.2.7 MSA clustering . . . 6
  - 1.2.8 Residue cropping . . . 7
  - 1.2.9 Featurization and model inputs . . . 8
- 1.3 Self-distillation dataset . . . 9
- 1.4 AlphaFold Inference . . . 10
- 1.5 Input embeddings . . . 13
- 1.6 Evoformer blocks . . . 14
  - 1.6.1 MSA row-wise gated self-attention with pair bias . . . 14

1.6.2 MSA column-wise gated self-attention . . . 15
1.6.3 MSA transition . . . 16
1.6.4 Outer product mean . . . 17
1.6.5 Triangular multiplicative update . . . 17
1.6.6 Triangular self-attention . . . 18
1.6.7 Transition in the pair stack . . . 20
1.7 Additional inputs . . . 20
1.7.1 Template stack . . . 20
1.7.2 Unclustered MSA stack . . . 21
1.8 Structure module . . . 23
1.8.1 Construction of frames from ground truth atom positions . . . 26
1.8.2 Invariant point attention (IPA) . . . 26
1.8.3 Backbone update . . . 28
1.8.4 Compute all atom coordinates . . . 29
1.8.5 Rename symmetric ground truth atoms . . . 30
1.8.6 Amber relaxation . . . 31
1.9 Loss functions and auxiliary heads . . . 32
1.9.1 Side chain and backbone torsion angle loss . . . 32
1.9.2 Frame aligned point error (FAPE) . . . 33
1.9.3 Chiral properties of AlphaFold and its loss . . . 34
1.9.4 Configurations with FAPE(X,Y) = 0 . . . 35
1.9.5 Metric properties of FAPE . . . 36
1.9.6 Model confidence prediction (pLDDT) . . . 37
1.9.7 TM-score prediction . . . 37
1.9.8 Distogram prediction . . . 39
1.9.9 Masked MSA prediction . . . 39
1.9.10 “Experimentally resolved” prediction . . . 39
1.9.11 Structural violations . . . 40
1.10 Recycling iterations . . . 41
1.11 Training and inference details . . . 43
1.11.1 Training stages . . . 43
1.11.2 MSA resampling and ensembling . . . 43
1.11.3 Optimization details . . . 44
1.11.4 Parameters initialization . . . 44
1.11.5 Loss clamping details . . . 44
1.11.6 Dropout details . . . 44
1.11.7 Evaluator setup . . . 45
1.11.8 Reducing the memory consumption . . . 45
1.12 CASP14 assessment . . . 46
1.12.1 Training procedure . . . 46
1.12.2 Inference and scoring . . . 46
1.13 Ablation studies . . . 47
1.13.1 Architectural details . . . 47
1.13.2 Procedure . . . 49
1.13.3 Results . . . 49
1.14 Network probing details . . . 51
1.15 Novel fold performance . . . 52
1.16 Visualization of attention . . . 54
1.17 Additional results . . . 56

# List of Supplementary Figures


1 Input feature embeddings . . . . . 11
2 MSA row-wise gated self-attention with pair bias . . . . . 15
3 MSA column-wise gated self-attention . . . . . 16
4 MSA transition layer . . . . . 16
5 Outer product mean . . . . . 17
6 Triangular multiplicative update using "outgoing" edges . . . . . 18
7 Triangular self-attention around starting node . . . . . 19
8 Invariant Point Attention Module . . . . . 27
9 Accuracy distribution of a model trained with dRMSD instead of the FAPE loss . . . . . 36
10 Accuracy of ablations depending on the MSA depth . . . . . 50
11 Performance on a set of novel structures . . . . . 53
12 Visualization of row-wise pair attention . . . . . 54
13 Visualization of attention in the MSA along sequences . . . . . 55
14 Median all-atom $\text{RMSD}_{95}$ on the CASP14 set . . . . . 56
15 RMSD histograms on the template-reduced recent PDB set. . . . . . 57


# List of Supplementary Tables


1 Input features to the model . . . . . 8
2 Rigid groups for constructing all atoms from given torsion angles . . . . . 24
3 Ambiguous atom names due to 180°-rotation-symmetry . . . . . 31
4 AlphaFold training protocol . . . . . 43
5 Training protocol for CASP14 models . . . . . 46
6 Quartiles of RMSD distributions on the template-reduced recent PDB set. . . . . . 57


# List of Algorithms


1 `MSABlockDeletion` MSA block deletion . . . . . 6
2 `Inference` AlphaFold Model Inference . . . . . 12
3 `InputEmbedder` Embeddings for initial representations . . . . . 13
4 `relpos` Relative position encoding . . . . . 13
5 `one_hot` One-hot encoding with nearest bin . . . . . 13
6 `EvoformerStack` Evoformer stack . . . . . 14
7 `MSARowAttentionWithPairBias` MSA row-wise gated self-attention with pair bias . . . . . 15
8 `MSAColumnAttention` MSA column-wise gated self-attention . . . . . 16
9 `MSATransition` Transition layer in the MSA stack . . . . . 17
10 `OuterProductMean` Outer product mean . . . . . 17
11 `TriangleMultiplicationOutgoing` Triangular multiplicative update using "outgoing" edges 18
12 `TriangleMultiplicationIncoming` Triangular multiplicative update using "incoming" edges 18
13 `TriangleAttentionStartingNode` Triangular gated self-attention around starting node . . . . 19
14 `TriangleAttentionEndingNode` Triangular gated self-attention around ending node . . . . . 20
15 `PairTransition` Transition layer in the pair stack . . . . . 20
16 `TemplatePairStack` Template pair stack . . . . . 21
17 `TemplatePointwiseAttention` Template pointwise attention . . . . . 21
18 `ExtraMsaStack` Extra MSA stack . . . . . 22
19 `MSAColumnGlobalAttention` MSA global column-wise gated self-attention . . . . . 22
20 `StructureModule` Structure module . . . . . 25

21 `rigidFrom3Points` Rigid from 3 points using the Gram–Schmidt process . . . . . . . . . . . 26
22 `InvariantPointAttention` Invariant point attention (IPA) . . . . . . . . . . . . . . . . . . . . . 28
23 `BackboneUpdate` Backbone update . . . . . . . . . . . . . . . . . . . . . . . . . . . . . . . . . 29
24 `computeAllAtomCoordinates` Compute all atom coordinates . . . . . . . . . . . . . . . . . . 30
25 `makeRotX` Make a transformation that rotates around the x-axis . . . . . . . . . . . . . . . . . 30
26 `renameSymmetricGroundTruthAtoms` Rename symmetric ground truth atoms . . . . . . . . . . 31
27 `torsionAngleLoss` Side chain and backbone torsion angle loss . . . . . . . . . . . . . . . . . 33
28 `computeFAPE` Compute the Frame aligned point error . . . . . . . . . . . . . . . . . . . . . . 34
29 `predictPerResidueLDDT` Predict model confidence pLDDT . . . . . . . . . . . . . . . . . . . 37
30 `RecyclingInference` Generic recycling inference procedure . . . . . . . . . . . . . . . . . . 41
31 `RecyclingTraining` Generic recycling training procedure . . . . . . . . . . . . . . . . . . . . 41
32 `RecyclingEmbedder` Embedding of Evoformer and Structure module outputs for recycling . . 42


# 1 Supplementary Methods

## 1.1 Notation

We denote the number of residues in the input primary sequence by $N_{\text{res}}$ (cropped during training), the number of templates used in the model by $N_{\text{templ}}$, the number of all available MSA sequences by $N_{\text{all\_seq}}$, the number of clusters after MSA clustering by $N_{\text{clust}}$, the number of sequences processed in the MSA stack by $N_{\text{seq}}$ (where $N_{\text{seq}} = N_{\text{clust}} + N_{\text{templ}}$), and the number of unclustered MSA sequences by $N_{\text{extra\_seq}}$ (after sub-sampling, see subsubsection 1.2.7 for details). Concrete values for these parameters are given in the training details (subsection 1.11). On the model side, we also denote the number of blocks in Evoformer-like stacks by $N_{\text{block}}$ (subsection 1.6), the number of ensembling iterations by $N_{\text{ensemble}}$ (subsubsection 1.11.2), and the number of recycling iterations by $N_{\text{cycle}}$ (subsection 1.10).

We present architectural details in Algorithms, where we use the following conventions. We use capitalized operator names when they encapsulate learnt parameters, e.g. we use $\mathrm{Linear}$ for a linear transformation with a weights matrix $W$ and a bias vector $\mathbf{b}$, and $\mathrm{LinearNoBias}$ for the linear transformation without the bias vector. Note that when we have multiple outputs from the $\mathrm{Linear}$ operator at the same line of an algorithm, we imply different trainable weights for each output. We use $\mathrm{LayerNorm}$ for the layer normalization [85] operating on the channel dimensions with learnable per-channel gains and biases. We also use capitalized names for random operators, such as those related to dropout. For functions without parameters we use lower case operator names, e.g. $\mathrm{sigmoid}$, $\mathrm{softmax}$, $\mathrm{stopgrad}$. We use $\odot$ for the element-wise multiplication, $\otimes$ for the outer product, $\oplus$ for the outer sum, and $\mathbf{a}^\top\mathbf{b}$ for the dot product of two vectors. Indices $i$, $j$, $k$ always operate on the residue dimension, indices $s$, $t$ on the sequence dimension, and index $h$ on the attention heads dimension. The channel dimension is implicit and we type the channel-wise vectors in bold, e.g. $\mathbf{z}_{ij}$. Algorithms operate on sets of such vectors, e.g. we use $\{\mathbf{z}_{ij}\}$ to denote all pair representations.

In the structure module, we denote Euclidean transformations corresponding to frames by $T = (R, \vec{\mathbf{t}})$, with $R \in \mathbb{R}^{3\times3}$ for the rotation and $\vec{\mathbf{t}} \in \mathbb{R}^3$ for the translation components. We use the $\circ$ operator to denote application of a transformation to an atomic position $\vec{\mathbf{x}} \in \mathbb{R}^3$:

$$\begin{aligned}\vec{\mathbf{x}}_{\text{result}} &= T \circ \vec{\mathbf{x}} \\ &= (R, \vec{\mathbf{t}}) \circ \vec{\mathbf{x}} \\ &= R\vec{\mathbf{x}} + \vec{\mathbf{t}}\,.\end{aligned}$$

The $\circ$ operator also denotes composition of Euclidean transformations:

$$\begin{aligned}T_{\text{result}} &= T_1 \circ T_2 \\ (R_{\text{result}}, \vec{\mathbf{t}}_{\text{result}}) &= (R_1, \vec{\mathbf{t}}_1) \circ (R_2, \vec{\mathbf{t}}_2) \\ &= (R_1 R_2,\ \ R_1\vec{\mathbf{t}}_2 + \vec{\mathbf{t}}_1)\end{aligned}$$

We use $T^{-1}$ to denote the group inverse of the transform $T$:

$$\begin{aligned} T^{-1} &= (R, \vec{\mathbf{t}})^{-1} \\ &= (R^{-1},\ -R^{-1}\vec{\mathbf{t}}) \end{aligned}$$

## 1.2 Data pipeline

The data pipeline is the first step when running AlphaFold. It takes an mmCIF file (in the training mode) or a FASTA file (in the inference mode) and produces input features for the model. In the training mode, a single mmCIF file can produce multiple separate training examples, one for each chain in the file. The data pipeline includes the following steps.

### 1.2.1 Parsing

The input file is parsed and basic metadata is extracted from it. For FASTA, this is only the sequence and name; for mmCIF this is the sequence, atom coordinates, release date, name, and resolution. We also resolve alternative locations for atoms/residues, taking the one with the largest occupancy, change MSE residues into MET residues, and fix arginine naming ambiguities (making sure that NH1 is always closer to CD than NH2).

### 1.2.2 Genetic search

Multiple genetic databases are searched using JackHMMER v3.3 [86] and HHBlits v3.0-beta.3 [87]. We used JackHMMER with MGnify [88], JackHMMER with UniRef90 [89], HHBlits with Uniclust30 [90] + BFD (see Input and Data Sources in the main text methods for details). The output multiple sequence alignments (MSAs) are de-duplicated and stacked.

The MSA depth was limited to 5,000 sequences for JackHMMER with MGnify, 10,000 sequences for JackHMMER with UniRef90 and unlimited for HHBlits. The following flags were set to a non-default value for each of the tools:

JackHMMER: -N 1 -E 0.0001 --incE 0.0001 --F1 0.0005 --F2 0.00005 --F3 0.0000005.

HHBlits: -n 3 -e 0.001 -realign_max 100000 -maxfilt 100000 -min_prefilter_hits 1000 -maxseq 1000000.

### 1.2.3 Template search

The structural templates fed to the model are retrieved with the following steps:

1. The UniRef90 MSA obtained in the previous step is used to search PDB70 using HHSearch [91]. The only flag set to a non-default value for HHSearch runs was -maxseq 1000000. Structural data for each hit is obtained from the corresponding mmCIF file in the PDB database. If the sequence from PDB70 does not exactly match the sequence in the mmCIF file then the two are aligned using Kalign[92].

2. During training we exclude all templates that were released after the query training structure. We also filter out templates that are identical to (or a subset of) the input primary sequence, or that are too small (less than 10 residues or of length less than 10% of the primary sequence).

3. At inference time we provide the model the top 4 templates, sorted by the expected number of correctly aligned residues (the "sum_probs" feature output by HHSearch). At training time we first restrict the available templates to up to 20 with the highest "sum_probs". Then we choose random $k$ templates out of this restricted set of $n$ templates, where $k = \min(\mathrm{Uniform}[0, n], 4)$. This has the effect of showing the network potentially bad templates, or no templates at all, during training so the network cannot rely on just copying the template.

### 1.2.4 Training data

With 75% probability a training example comes from the self-distillation set (see subsection 1.3) and with 25% probability the training example is a known structure from the Protein Data Bank. We loop over this hybrid set multiple times during training and we apply a number of stochastic filters (subsubsection 1.2.5), MSA preprocessing steps (subsubsection 1.2.6 and subsubsection 1.2.7), and residue cropping (subsubsection 1.2.8) every time when a protein is encountered. This means, we may observe different targets in training epochs, with different samples of the MSA data, and also cropped to different regions.

### 1.2.5 Filtering

The following filters are applied to the training data:

- Input mmCIFs are restricted to have resolution less than 9 Å. This is not a very restrictive filter and only removes around 0.2% of structures.
- Protein chains are accepted with probability $\frac{1}{512}\max(\min(N_{\text{res}}, 512), 256)$, where $N_{\text{res}}$ is the length of the chain. This re-balances the length distribution and makes the network train on crops from the longer chains more often.
- Sequences are filtered out when any single amino acid accounts for more than 80% of the input primary sequence. This filter removes about 0.8% of sequences.
- Protein chains are accepted with the probability inverse to the size of the cluster that this chain falls into. We use 40% sequence identity clusters of the Protein Data Bank clustered with MMSeqs2 [93].

### 1.2.6 MSA block deletion

During training contiguous blocks of sequences are deleted from the MSA (see Algorithm 1). The MSA is grouped by tool and ordered by the normal output of each tool, typically e-value. This means that similar sequences are more likely to be adjacent in the MSA and block deletions are more likely to generate diversity that removes whole branches of the phylogeny.

**Algorithm 1** MSA block deletion

**def** MSABlockDeletion($\mathbf{msa}$) :
block_size $= \lfloor 0.3 \cdot N_{\text{all\_seq}} \rfloor$
to_delete $= \{\}$
**for all** $j \in [1, \ldots, 5]$ **do**
  block_start $\leftarrow$ uniform$(1, N_{\text{all_seq}})$
  to_delete $\leftarrow$ to_delete $\cup$ [block_start, …, block_start + block_size − 1]
**end for**
keep $\leftarrow [1, \ldots, N_{\text{all\_seq}}] \setminus$ to_delete
$\mathbf{msa} \leftarrow \mathbf{msa}_{\text{keep}}$
**return** $\mathbf{msa}$

### 1.2.7 MSA clustering

The computational and peak memory cost of the main Evoformer module scales as $N_{\text{seq}}^2 \times N_{\text{res}}$, so it is highly desirable to reduce the number of sequences used in the main Evoformer module for purely computational reasons. Our first version of this procedure randomly chose a fixed-size subset of sequences without replacement when the MSA was too large (originally 128 sequences). This procedure has the clear downside that

sequences not included in the random subset have no influence on the prediction. The presented version is a modification of this procedure where we still choose a random subset of sequences without replacement to be representatives, but for each sequence in the full MSA we associate that sequence with the nearest sequence in the set of representatives (we call this "clustering" with random cluster centres though no attempt is made to ensure the cluster centres are well-distributed). To maintain the fixed-size and bounded cost properties, so we use only the amino acid and deletion frequencies of all sequences associated with each representative sequence and provide these mini-profiles as extra features in addition to the representative sequence (we roughly double the number of input features of each representative sequence without increasing the number of representative sequences). This allows all sequences to have some influence on the final prediction, which seems desirable.

Since this procedure achieves the goal of bounding computational cost, we have not experimented much with alternatives to the procedure. We have attempted a couple of methods to encourage diversity among the sampled sequences at inference time (e.g. a bias to pick representatives far from each other), but gains were very small to none so we did not pursue them further.

In detail the MSA is clustered (grouped) using the following procedure:

1. $N_{\text{clust}}$ sequences are selected randomly as MSA cluster centres, the first cluster centre is always set to the query amino acid sequence.

2. A mask is generated such that each position in a MSA cluster centre has a 15% probability of being included in the mask. Each element in the MSA that is included in the mask is replaced in the following way:

   - With 10% probability amino acids are replaced with a uniformly sampled random amino acid.
   - With 10% probability amino acids are replaced with an amino acid sampled from the MSA profile for a given position.
   - With 10% probability amino acids are not replaced.
   - With 70% probability amino acids are replaced with a special token (masked_msa_token).

   These masked positions are the prediction targets used in subsubsection 1.9.9. Note that this masking is used both at training time, and at inference time.

3. The remaining sequences are assigned to their closest cluster by Hamming distance (ignoring masked out residues and gaps). For each sequence cluster, several statistics are computed, such as the per residue amino acid distribution). See "cluster" features in Table 1 for a full description.

4. The MSA sequences that have not been selected as cluster centres at step 1 are used to randomly sample $N_{\text{extra\_seq}}$ sequences without replacement. If there are less than $N_{\text{extra\_seq}}$ remaining sequences available, all of them are used. These sequences form "extra" MSA features in Table 1.

Please note that throughout the rest of the manuscript we use the term "MSA clusters" to refer to the clusters produced at step 3 above.

### 1.2.8 Residue cropping

During training the residue dimension in all data is cropped in the following way.

1. As described in subsubsection 1.11.5 mini-batches are processed in two modes. In the unclamped loss mode the crop start position is sampled from Uniform$[1, n - x + 1]$ where $n$ is seq_length minus crop_size and $x$ is sampled from Uniform$[0, n]$. In clamped loss mode the crop position is sampled from Uniform$[1, n + 1]$.

2. The residues dimension is cropped to a single contiguous region, with the crop start position defined above. The final crop size is denoted by $N_{\text{res}}$ and its concrete value is provided in subsection 1.11.

### 1.2.9 Featurization and model inputs

Features in Table 1 are computed and aggregated into the following main inputs to the model:

- **target_feat** This is a feature of size $[N_{\text{res}}, 21]$ consisting of the "aatype" feature.
- **residue_index** This is a feature of size $[N_{\text{res}}]$ consisting of the "residue_index" feature.
- **msa_feat** This is a feature of size $[N_{\text{clust}}, N_{\text{res}}, 49]$ constructed by concatenating "cluster_msa", "cluster_has_deletion", "cluster_deletion_value", "cluster_deletion_mean", "cluster_profile". We draw $N_{\text{cycle}} \times N_{\text{ensemble}}$ random samples from this feature to provide each recycling/ensembling iteration of the network with a different sample (see subsubsection 1.11.2).
- **extra_msa_feat** This is a feature of size $[N_{\text{extra\_seq}}, N_{\text{res}}, 25]$ constructed by concatenating "extra_msa", "extra_msa_has_deletion", "extra_msa_deletion_value". Together with "msa_feat' above we also draw $N_{\text{cycle}} \times N_{\text{ensemble}}$ random samples from this feature (see subsubsection 1.11.2).
- **template_pair_feat** This is a feature of size $[N_{\text{templ}}, N_{\text{res}}, N_{\text{res}}, 88]$ and consists of concatenation of the pair residue features "template_distogram", "template_unit_vector", and also several residue features, which are transformed into pair features. The "template_aatype" feature is included via tiling and stacking (this is done twice, in both residue directions). Also the mask features "template_pseudo_beta_mask" and "template_backbone_frame_mask" are included, where the feature $f_{ij} = \text{mask}_i \cdot \text{mask}_j$.
- **template_angle_feat** This is a feature of size $[N_{\text{templ}}, N_{\text{res}}, 51]$ constructed by concatenating the following features: "template_aatype", "template_torsion_angles", "template_alt_torsion_angles", and "template_torsion_angles_mask".

**Table 1** | Input features to the model. Feature dimensions: $N_{\text{res}}$ is the number of residues, $N_{\text{clust}}$ is the number of MSA clusters, $N_{\text{extra\_seq}}$ is the number of additional unclustered MSA sequences, and $N_{\text{templ}}$ is the number of templates.

| **Feature & Shape** | **Description** |
|---|---|
| aatype<br>$[N_{\text{res}}, 21]$ | One-hot representation of the input amino acid sequence (20 amino acids + unknown). |
| cluster_msa<br>$[N_{\text{clust}}, N_{\text{res}}, 23]$ | One-hot representation of the msa cluster centre sequences (20 amino acids + unknown + gap + masked_msa_token). |
| cluster_has_deletion<br>$[N_{\text{clust}}, N_{\text{res}}, 1]$ | A binary feature indicating if there is a deletion to the left of the residue in the MSA cluster centres. |
| cluster_deletion_value<br>$[N_{\text{clust}}, N_{\text{res}}, 1]$ | The raw deletion counts (the number of deletions to the left of every position in the MSA cluster centres) are transformed to the range $[0, 1]$ using $\frac{2}{\pi} \arctan \frac{d}{3}$ where $d$ are the raw counts. |
| cluster_deletion_mean<br>$[N_{\text{clust}}, N_{\text{res}}, 1]$ | The mean deletions for every residue in every cluster are computed as $\frac{1}{n} \sum_{i=1}^{n} d_{ij}$ where $n$ is the number of sequences in the cluster and $d_{ij}$ is the number of deletions to the left of the ith sequence and jth residue. These are then transformed into the range $[0, 1]$ in the same way as for the cluster_deletion_value feature above. |
| cluster_profile<br>$[N_{\text{clust}}, N_{\text{res}}, 23]$ | The distribution across amino acid types for each residue in each MSA cluster (20 amino acids + unknown + gap + masked_msa_token). |

| **Feature & Shape** | **Description** |
|---|---|
| extra_msa<br>$[N_{\text{extra\_seq}}, N_{\text{res}}, 23]$ | One-hot representation of all MSA sequences not selected as cluster centres (20 amino acids + unknown + gap + masked_msa_token). |
| extra_msa_has_deletion<br>$[N_{\text{extra\_seq}}, N_{\text{res}}, 1]$ | A binary feature indicating if there is a deletion to the left of the residue in the extra MSA sequences. |
| extra_msa_deletion_value<br>$[N_{\text{extra\_seq}}, N_{\text{res}}, 1]$ | The raw deletion counts to the left of every residue in the extra_msa, converted to the range $[0, 1]$ using the same formula as for cluster_deletion_value. |
| template_aatype<br>$[N_{\text{templ}}, N_{\text{res}}, 22]$ | One-hot representation of the amino acid sequence (20 amino acids + unknown and gap). |
| template_mask<br>$[N_{\text{templ}}, N_{\text{res}}]$ | Mask indicating if a template residue exists and has coordinates. |
| template_pseudo_beta_mask<br>$[N_{\text{templ}}, N_{\text{res}}]$ | Mask indicating if the beta carbon (alpha carbon for glycine) atom has coordinates for the template at this residue. |
| template_backbone_frame_mask<br>$[N_{\text{templ}}, N_{\text{res}}]$ | A mask indicating if the coordinates of all the required atoms to compute the backbone frame (used in the template_unit_vector feature) exist. |
| template_distogram<br>$[N_{\text{templ}}, N_{\text{res}}, N_{\text{res}}, 39]$ | A one-hot pairwise feature indicating the distance between beta carbons (alpha carbon used for glycine) atoms. The pairwise distances are discretized into 38 bins of equal width between $3.25$ Å and $50.75$ Å; and one more bin contains any larger distances. |
| template_unit_vector<br>$[N_{\text{templ}}, N_{\text{res}}, N_{\text{res}}, 3]$ | The unit vector of the displacement of the alpha carbon atom of all residues within the local frame of each residue. These local frames are computed in the same way as for the target structure, see subsubsection 1.8.1. (Current models were trained with this feature set to zero.) |
| template_torsion_angles<br>$[N_{\text{templ}}, N_{\text{res}}, 14]$ | The 3 backbone torsion angles and up to 4 side-chain torsion angles for each residue represented as sine and cosine encoding. |
| template_alt_torsion_angles<br>$[N_{\text{templ}}, N_{\text{res}}, 14]$ | Alternative torsion angles for side chain parts with $180^o$-rotation symmetry. |
| template_torsion_angles_mask<br>$[N_{\text{templ}}, N_{\text{res}}, 14]$ | A mask indicating if the torsion angle is present in the template structure. |
| residue_index<br>$[N_{\text{res}}]$ | The index into the original amino acid sequence. |

## 1.3 Self-distillation dataset

We perform a self-distillation procedure similar to [94] on unlabelled protein sequences. To create the sequence dataset we compute multiple sequence alignments of every cluster in Uniclust30 (version 2018-08) against the same database. We then greedily remove sequences which appear in another sequence's MSA to give 6,318,986 sequences in total. We further filter this by removing sequences with more than 1,024 or fewer

than 200 amino acids, or whose MSA contain fewer than 200 alignments. This gives a final dataset of 355,993 sequences.

For building the distillation set predicted structures, we use a single (no re-ranking) “undistilled” model, i.e. trained on just the PDB dataset. Using this undistilled model, we predict the structure of every sequence in the set constructed above to create a dataset of structures to use at training time. For every pair of predicted residues we calculate a confidence metric by computing the Kullback–Leibler divergence between the pairwise distance distribution and a reference distribution for that residue separation:

$$c_{ij} = D_{KL}\left(p^{\text{ref}}_{|i-j|}(r) \middle|\middle| p_{i,j}(r)\right). \tag{1}$$

The reference distribution is computed by taking distance distribution predictions for 1,000 randomly sampled sequences in Uniclust30 and computing the mean distribution for a given sequence separation.

This pairwise metric $c_{ij}$ is then averaged over $j$ corresponding to a maximum sequence separation of $\pm 128$ and excluding $i = j$ to give a per-residue confidence metric $c_i$. Similar to pLDDT, we find that higher values of this confidence correlate with higher prediction accuracy. At training time we mask out residues with $c_i < 0.5$. This KL-based metric was introduced into the model before we developed pLDDT, and we expect that filtering the distillation set based on pLDDT would work equally well or better.

At training time, extra augmentation is added to distillation dataset examples by uniformly sampling the MSA to $1000$ sequences without replacement (this is on top of any sampling that happens in the data pipeline, see subsection 1.2).

## 1.4 AlphaFold Inference

For inference, the AlphaFold receives input features derived from the amino-acid sequence, MSA, and templates (see subsubsection 1.2.9) and outputs features including atom coordinates, the distogram, and per-residue confidence scores. Algorithm 2 outlines the main steps (see also Fig 1e and the corresponding description in the main article).

The whole network is executed sequentially $N_{\text{cycle}}$ times, where the outputs of the former execution are recycled as inputs for the next execution (see subsection 1.10 for details). The first part of the network (feature embedding and Evoformer, see Suppl. Fig. 1) is executed independently $N_{\text{ensemble}}$ times with differently sampled inputs (see subsubsection 1.11.2). Their outputs are averaged to create the inputs for the Structure module and for recycling. These averaged Evoformer embeddings are denoted with an extra hat, e.g. $\hat{z}_{ij}$ for pair representations and $\hat{s}_i$ for single representations. The $N_{\text{cycle}} \times N_{\text{ensemble}}$ random samples of “msa_feat” and “extra_msa_feat” are denoted as a set of sets $\left\{\{\mathbf{f}^{\text{msa\_feat}}_{sci}\}_{c,n}\right\}$, and $\left\{\{\mathbf{f}^{\text{extra\_msa\_feat}}_{sei}\}_{c,n}\right\}$. Please note that ensembling is used only during inference and it has a very minor impact on the accuracy in the final system (subsubsection 1.12.2), thus can be considered as an optional technique.

The first part of the network (Algorithm 2 line 5) starts with embedding a new sample from the MSA (see subsection 1.5 for details) to create the initial version of the MSA representation $\{\mathbf{m}_{si}\}$, and the pair representation $\{\mathbf{z}_{ij}\}$. The first row of the MSA representation and the full pair representation are updated (Algorithm 2 line 6) by the recycled outputs from the previous iteration (see subsection 1.10 for details) – for the first iteration the recycled outputs are initialized to zero.

The next steps integrate the information from the templates (Algorithm 2 line 7). The “template_angle_feat” (derived from amino acid types and the torsion angles) are embedded by a shallow MLP and concatenated to the MSA representation. The “template_pair_feat” (derived from residue pairs, see subsubsection 1.2.9) are embedded by a shallow attention network (see subsubsection 1.7.1 for details) and added to the pair representation.

The final step of the embedding procedure (Algorithm 2 line 14) processes the extra MSA features (derived from individual unclustered MSA sequences) by shallow Evoformer-like network that is optimized for the large number of sequences (see subsubsection 1.7.2) to update the pair representation.

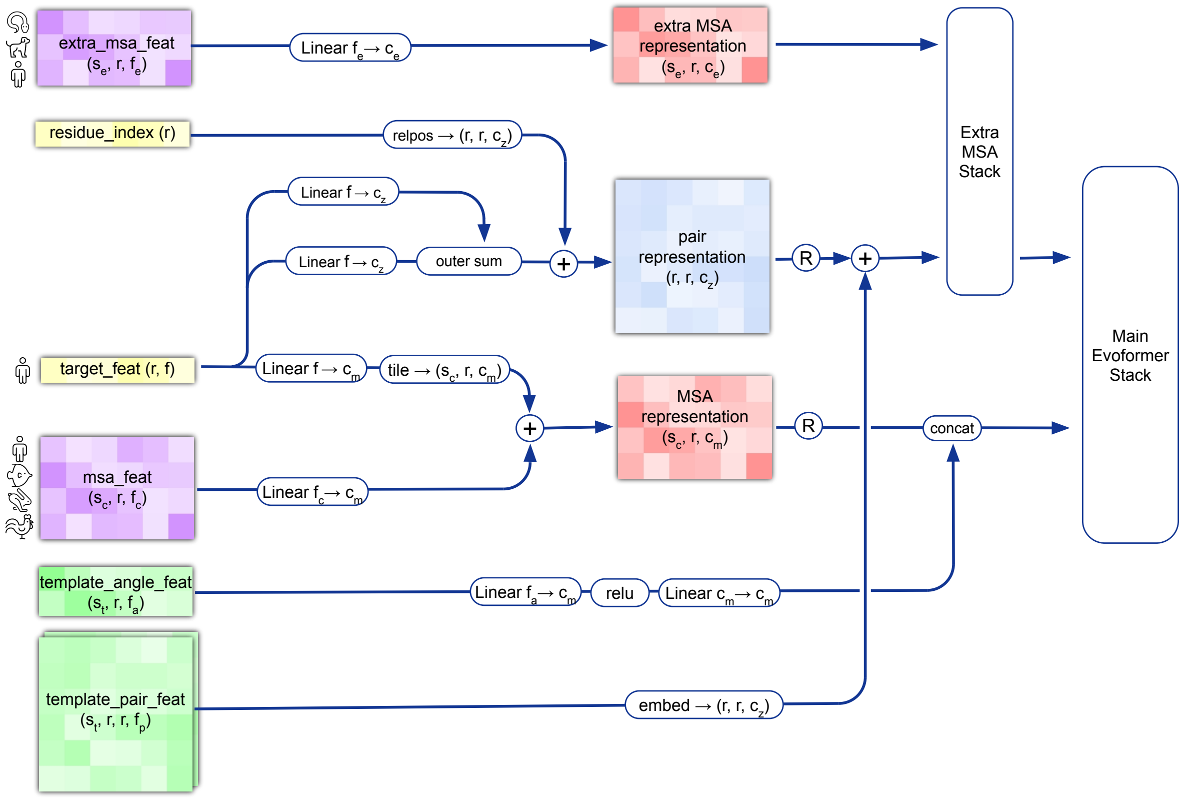


**Supplementary Figure 1** | Input feature embeddings. Dimension names: $r$: residues, $f$: features, $c$: channels, $s_c$: clustered MSA sequences, $s_e$: extra MSA sequences, $s_t$: template sequences. Operator $\mathrm{relpos}$ is defined in Algorithm 4. Template embedding and extra MSA stack are explained in subsection 1.7. Recycling injection is denoted with R, see subsection 1.10 for details.

The main trunk of the network is the Evoformer stack (subsection 1.6) that produces the final representations (Algorithm 2 line 17). The pair representation, the first row of the MSA representation and a single representation derived from that row are kept for recycling and the structure module.

The second part of the network, the Structure Module (Algorithm 2 line 21), takes the final representations as input and predicts the atom coordinates $\{\vec{\mathbf{x}}_i^a\}$ and the per-residue confidence $\{r_i^{\mathrm{pLDDT}}\}$ (see subsection 1.8).

**Algorithm 2** AlphaFold Model Inference

**def** Inference $\left(\{\mathbf{f}_i^{\text{target\_feat}}\}, \{f_i^{\text{residue\_index}}\}, \left\{\{\mathbf{f}_{s_c i}^{\text{msa\_feat}}\}_{c,n}\right\}, \left\{\{\mathbf{f}_{s_e i}^{\text{extra\_msa\_feat}}\}_{c,n}\right\},\right.$
$\left.\{\mathbf{f}_{s_t i}^{\text{template\_angle\_feat}}\}, \{\mathbf{f}_{s_t ij}^{\text{template\_pair\_feat}}\}, N_{\text{cycle}} = 4, N_{\text{ensemble}} = 3\right)$ :

*# Recycling iterations:*

$\hat{\mathbf{m}}_{1i}^{\text{prev}}, \hat{\mathbf{z}}_{ij}^{\text{prev}}, \vec{\mathbf{x}}_i^{\text{prev,C}^\beta} = \mathbf{0}, \mathbf{0}, \vec{\mathbf{0}}$

**for all** $c \in [1, \ldots, N_{\text{cycle}}]$ **do** *# shared weights*

*# Average representations in ensemble:*

$\{\hat{\mathbf{m}}_{1i}\}, \{\hat{\mathbf{z}}_{ij}\}, \{\hat{\mathbf{s}}_i\} = \mathbf{0}, \mathbf{0}, \mathbf{0}$

**for all** $n \in [1, \ldots, N_{\text{ensemble}}]$ **do** *# shared weights*

*# Embed clustered MSA (use different MSA samples in each iteration):*

$\{\mathbf{m}_{s_c i}\}, \{\mathbf{z}_{ij}\} = \text{InputEmbedder}(\{\mathbf{f}_i^{\text{target\_feat}}\}, \{f_i^{\text{residue\_index}}\}, \{\mathbf{f}_{s_c i}^{\text{msa\_feat}}\}_{c,n})$

*# Inject previous outputs for recycling:*

$\{\mathbf{m}_{1i}\}, \{\mathbf{z}_{ij}\} \mathrel{+}= \text{RecyclingEmbedder}(\{\hat{\mathbf{m}}_{1i}^{\text{prev}}\}, \{\hat{\mathbf{z}}_{ij}^{\text{prev}}\}, \{\vec{\mathbf{x}}_i^{\text{prev,C}^\beta}\})$

*# Embed templates:*

$\mathbf{a}_{s_t i} \leftarrow \text{Linear}(\text{relu}(\text{Linear}(\mathbf{f}_{s_t i}^{\text{template\_angle\_feat}})))$ $\qquad \mathbf{a}_{s_t i} \in \mathbb{R}^{c_m}, c_m = 256$

$\mathbf{m}_{si} = \text{concat}_s(\mathbf{m}_{s_c i}, \mathbf{a}_{s_t i})$

$\mathbf{t}_{s_t ij} = \text{Linear}(\mathbf{f}_{s_t ij}^{\text{template\_pair\_feat}})$ $\qquad \mathbf{t}_{s_t ij} \in \mathbb{R}^{c_t}, c_t = 64$

**for all** $s_t \in [1, \ldots, N_{\text{templ}}]$ **do** *# shared weights*

$\{\mathbf{t}_{s_t ij}\} \leftarrow \text{TemplatePairStack}(\{\mathbf{t}_{s_t ij}\})$

**end for**

$\{\mathbf{z}_{ij}\} \mathrel{+}= \text{TemplatePointwiseAttention}(\{\mathbf{t}_{s_t ij}\}, \{\mathbf{z}_{ij}\})$

*# Embed extra MSA features (use different samples in each iteration):*

$\{\mathbf{a}_{s_e i}\} \leftarrow \{\mathbf{f}_{s_e i}^{\text{extra\_msa\_feat}}\}_{c,n}$

$\mathbf{e}_{s_e i} = \text{Linear}(\mathbf{a}_{s_e i})$ $\qquad \mathbf{e}_{s_e i} \in \mathbb{R}^{c_e}, c_e = 64$

$\{\mathbf{z}_{ij}\} \leftarrow \text{ExtraMsaStack}(\{\mathbf{e}_{s_e i}\}, \{\mathbf{z}_{ij}\})$

*# Main trunk of the network:*

$\{\mathbf{m}_{si}\}, \{\mathbf{z}_{ij}\}, \{\mathbf{s}_i\} \leftarrow \text{EvoformerStack}(\{\mathbf{m}_{si}\}, \{\mathbf{z}_{ij}\})$

$\{\hat{\mathbf{m}}_{1i}\}, \{\hat{\mathbf{z}}_{ij}\}, \{\hat{\mathbf{s}}_i\} \mathrel{+}= \{\mathbf{m}_{1i}\}, \{\mathbf{z}_{ij}\}, \{\mathbf{s}_i\}$

**end for**

$\{\hat{\mathbf{m}}_{1i}\}, \{\hat{\mathbf{z}}_{ij}\}, \{\hat{\mathbf{s}}_i\} \mathrel{/}= N_{\text{ensemble}}$

*# Structure and confidence prediction:*

$\{\vec{\mathbf{x}}_i^a\}, \{r_i^{\text{pLDDT}}\} = \text{StructureModule}(\{\hat{\mathbf{s}}_i\}, \{\hat{\mathbf{z}}_{ij}\})$

$\{\hat{\mathbf{m}}_{1i}^{\text{prev}}\}, \{\hat{\mathbf{z}}_{ij}^{\text{prev}}\}, \{\vec{\mathbf{x}}_i^{\text{prev,C}^\beta}\} \leftarrow \{\hat{\mathbf{m}}_{1i}\}, \{\hat{\mathbf{z}}_{ij}\}, \{\vec{\mathbf{x}}_i^{\text{C}^\beta}\}$

**end for**

**return** $\{\vec{\mathbf{x}}_i^a\}, \{r_i^{\text{pLDDT}}\}$

## 1.5 Input embeddings

Input features listed in subsubsection 1.2.9 are embedded into the network according to the Suppl. Fig. 1. Initial embedding details are also presented in Algorithm 3. Input primary sequence and MSA features are transformed to form MSA representation $\{\mathbf{m}_{si}\}$ with $\mathbf{m}_{si} \in \mathbb{R}^{c_m}$, $c_m = 256$, and $s \in \{1 \ldots N_{\text{seq}}\}$, $i \in \{1 \ldots N_{\text{res}}\}$. Primary sequence features are transformed to form pair representation $\{\mathbf{z}_{ij}\}$ with $\mathbf{z}_{ij} \in \mathbb{R}^{c_z}$, $c_z = 128$, and $i, j \in \{1 \ldots N_{\text{res}}\}$. These two representations are also informed by the templates and unclustered MSA features. The details of their corresponding embedding modules are provided in subsection 1.7.

**Algorithm 3** Embeddings for initial representations

**def** InputEmbedder$(\{\mathbf{f}_i^{\text{target_feat}}\}, \{f_i^{\text{residue_index}}\}, \{\mathbf{f}_{s_c i}^{\text{msa_feat}}\})$ :

$\mathbf{a}_i, \mathbf{b}_i = \text{Linear}(\mathbf{f}_i^{\text{target\_feat}})$ $\qquad \mathbf{a}_i, \mathbf{b}_i \in \mathbb{R}^{c_z}, c_z = 128$

$\mathbf{z}_{ij} = \mathbf{a}_i + \mathbf{b}_j$ $\qquad \mathbf{z}_{ij} \in \mathbb{R}^{c_z}, c_z = 128$

$\{\mathbf{z}_{ij}\}$ += relpos$(\{f_i^{\text{residue_index}}\})$

$\mathbf{m}_{s_c i} = \text{Linear}(\mathbf{f}_{s_c i}^{\text{msa\_feat}}) + \text{Linear}(\mathbf{f}_i^{\text{target\_feat}})$ $\qquad \mathbf{m}_{s_c i} \in \mathbb{R}^{c_m}, c_m = 256$

**return** $\{\mathbf{m}_{s_c i}\}, \{\mathbf{z}_{ij}\}$

To provide the network with information about the positions of residues in the chain, we also encode relative positional features (Algorithm 4) into the initial pair representations. Specifically, for a residue pair $i, j \in \{1 \ldots N_{\text{res}}\}$ we compute the clipped relative distance within a chain, encode it as a one-hot vector, and add this vector's linear projection to $\mathbf{z}_{ij}$. Since we are clipping by the maximum value 32, any larger distances within the residue chain will not be distinguished by this feature. This inductive bias de-emphasizes primary sequence distances. Compared to the more traditional approach of encoding positions in the frequency space [95], this relative encoding scheme empirically allows the network to be evaluated without quality degradation on much longer sequences than it was trained on. A related construction was used in [96].

**Algorithm 4** Relative position encoding

**def** relpos$(\{f_i^{\text{residue_index}}\}, \mathbf{v}_{\text{bins}} = [-32, -31, \ldots, 32])$ :

$d_{ij} = f_i^{\text{residue\_index}} - f_j^{\text{residue\_index}}$ $\qquad d_{ij} \in \mathbb{Z}$

$\mathbf{p}_{ij} = \text{Linear}(\text{one\_hot}(d_{ij}, \mathbf{v}_{\text{bins}}))$ $\qquad \mathbf{p}_{ij} \in \mathbb{R}^{c_z}$

**return** $\{\mathbf{p}_{ij}\}$

**Algorithm 5** One-hot encoding with nearest bin

**def** one_hot$(x, \mathbf{v}_{\text{bins}})$ : $\qquad x \in \mathbb{R}, \quad \mathbf{v}_{\text{bins}} \in \mathbb{R}^{N_{\text{bins}}}$

$\mathbf{p} = \mathbf{0}$ $\qquad \mathbf{p} \in \mathbb{R}^{N_{\text{bins}}}$

$b = \arg\min(|x - \mathbf{v}_{\text{bins}}|)$

$p_b = 1$

**return** $\mathbf{p}$

## 1.6 Evoformer blocks

The network has a two tower architecture with axial self-attention in the MSA stack; triangular multiplicative updates and triangular self-attention in the pair stack; and outer product mean and attention biasing to allow communication between the stacks.

The main trunk of the network consists of $N_{\text{block}} = 48$ Evoformer blocks (**Fig. 1e, 3a**, Algorithm 6). Each block has an MSA representation $\{\mathbf{m}_{si}\}$ and a pair representation $\{\mathbf{z}_{ij}\}$ as its input and output and processes them with several layers. Each layer output is added via a residual connection to the current representations. Some layer outputs are passed through Dropout [97] before they are added (see subsubsection 1.11.6 for details).

The final Evoformer block provides a highly processed MSA representation $\{\mathbf{m}_{si}\}$ and a pair representation $\{\mathbf{z}_{ij}\}$, which contain information required for the structure module (subsection 1.8) and auxiliary network heads (subsection 1.9). The prediction modules are also using a "single" sequence representation $\{\mathbf{s}_i\}$ with $\mathbf{s}_i \in \mathbb{R}^{c_s}$, $c_s = 384$ and $i \in \{1 \dots N_{\text{res}}\}$. This single representation is derived by a linear projection of the first row of the MSA representation.

We now present details of the individual layers.

**Algorithm 6** Evoformer stack

**def** $\text{EvoformerStack}(\{\mathbf{m}_{si}\}, \{\mathbf{z}_{ij}\}, N_{\text{block}} = 48, c_s = 384):$

**for all** $l \in [1, \dots, N_{\text{block}}]$ **do**

*# MSA stack*

$\{\mathbf{m}_{si}\}$ += $\text{DropoutRowwise}_{0.15}(\text{MSARowAttentionWithPairBias}(\{\mathbf{m}_{si}\}, \{\mathbf{z}_{ij}\}))$

$\{\mathbf{m}_{si}\}$ += $\text{MSAColumnAttention}(\{\mathbf{m}_{si}\})$

$\{\mathbf{m}_{si}\}$ += $\text{MSATransition}(\{\mathbf{m}_{si}\})$

*# Communication*

$\{\mathbf{z}_{ij}\}$ += $\text{OuterProductMean}(\{\mathbf{m}_{si}\})$

*# Pair stack*

$\{\mathbf{z}_{ij}\}$ += $\text{DropoutRowwise}_{0.25}(\text{TriangleMultiplicationOutgoing}(\{\mathbf{z}_{ij}\}))$

$\{\mathbf{z}_{ij}\}$ += $\text{DropoutRowwise}_{0.25}(\text{TriangleMultiplicationIncoming}(\{\mathbf{z}_{ij}\}))$

$\{\mathbf{z}_{ij}\}$ += $\text{DropoutRowwise}_{0.25}(\text{TriangleAttentionStartingNode}(\{\mathbf{z}_{ij}\}))$

$\{\mathbf{z}_{ij}\}$ += $\text{DropoutColumnwise}_{0.25}(\text{TriangleAttentionEndingNode}(\{\mathbf{z}_{ij}\}))$

$\{\mathbf{z}_{ij}\}$ += $\text{PairTransition}(\{\mathbf{z}_{ij}\})$

**end for**

*# Extract the single representation*

$\mathbf{s}_i = \text{Linear}(\mathbf{m}_{1i})$ $\quad$ $\mathbf{s}_i \in \mathbb{R}^{c_s}$

**return** $\{\mathbf{m}_{si}\}, \{\mathbf{z}_{ij}\}, \{\mathbf{s}_i\}$

### 1.6.1 MSA row-wise gated self-attention with pair bias

MSA representations are processed with consecutive blocks of gated row-wise and column-wise self-attention blocks. The row-wise version (Suppl. Fig. 2 and Algorithm 7) builds attention weights for residue pairs and integrates the information from the pair representation as an additional bias term (line 3). This allows communication from the pair stack into the MSA stack to encourage consistency between them.

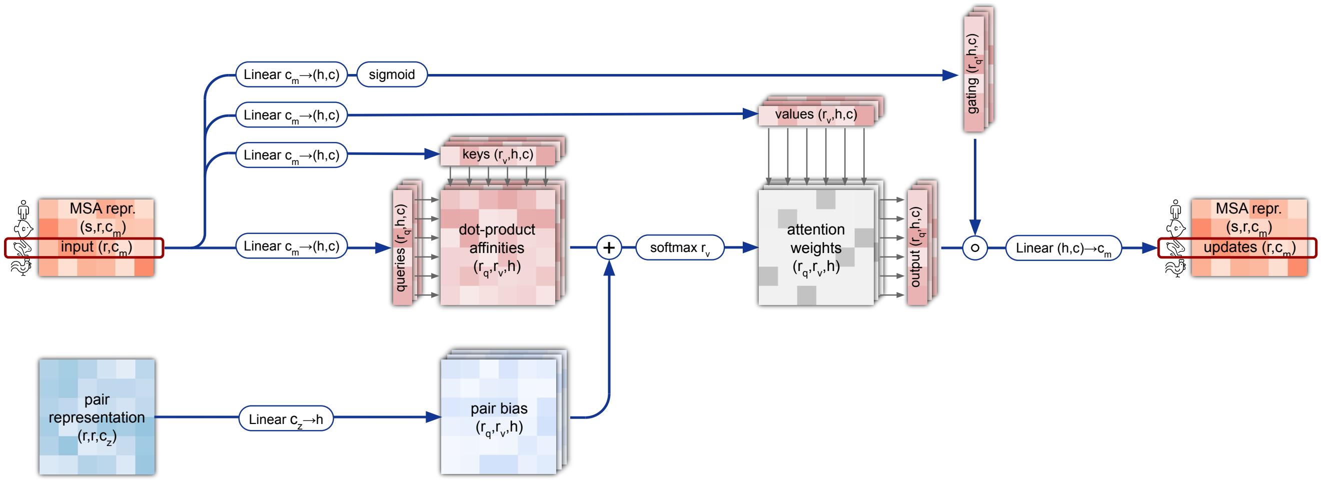


**Supplementary Figure 2** | MSA row-wise gated self-attention with pair bias. Dimensions: s: sequences, r: residues, c: channels, h: heads.

**Algorithm 7** MSA row-wise gated self-attention with pair bias

**def** MSARowAttentionWithPairBias($\{\mathbf{m}_{si}\}, \{\mathbf{z}_{ij}\}, c = 32, N_{\text{head}} = 8$) :

*# Input projections*

1: $\mathbf{m}_{si} \leftarrow \text{LayerNorm}(\mathbf{m}_{si})$

2: $\mathbf{q}_{si}^h, \mathbf{k}_{si}^h, \mathbf{v}_{si}^h = \text{LinearNoBias}(\mathbf{m}_{si})$ $\qquad \mathbf{q}_{si}^h, \mathbf{k}_{si}^h, \mathbf{v}_{si}^h \in \mathbb{R}^c,\ h \in \{1, \dots, N_{\text{head}}\}$

3: $b_{ij}^h = \text{LinearNoBias}(\text{LayerNorm}(\mathbf{z}_{ij}))$

4: $\mathbf{g}_{si}^h = \text{sigmoid}\left(\text{Linear}(\mathbf{m}_{si})\right)$ $\qquad \mathbf{g}_{si}^h \in \mathbb{R}^c$

*# Attention*

5: $a_{sij}^h = \text{softmax}_j \left( \frac{1}{\sqrt{c}}\, \mathbf{q}_{si}^{h\top} \mathbf{k}_{sj}^h + b_{ij}^h \right)$

6: $\mathbf{o}_{si}^h = \mathbf{g}_{si}^h \odot \sum_j a_{sij}^h \mathbf{v}_{sj}^h$

*# Output projection*

7: $\tilde{\mathbf{m}}_{si} = \text{Linear}\left(\text{concat}_h(\mathbf{o}_{si}^h)\right)$ $\qquad \tilde{\mathbf{m}}_{si} \in \mathbb{R}^{c_m}$

8: **return** $\{\tilde{\mathbf{m}}_{si}\}$

### 1.6.2 MSA column-wise gated self-attention

The column-wise attention (and Suppl. Fig. 3 and Algorithm 8) lets the elements that belong to the same target residue exchange information. In both attention blocks the number of heads $N_{\text{heads}} = 8$, and dimension of the keys, queries, and values $c = 32$.

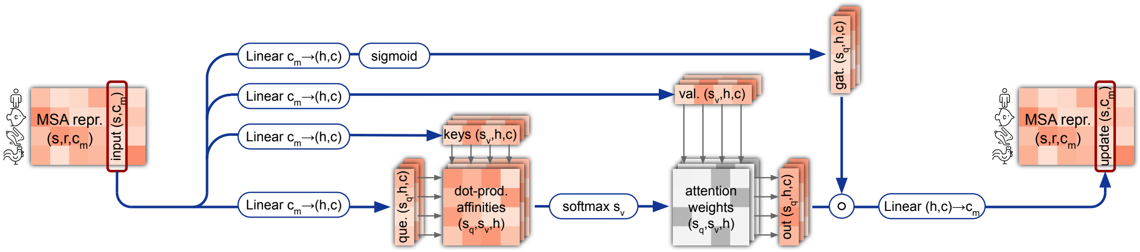


**Supplementary Figure 3** | MSA column-wise gated self-attention. Dimensions: s: sequences, r: residues, c: channels, h: heads.

**Algorithm 8** MSA column-wise gated self-attention

**def** $\mathrm{MSAColumnAttention}(\{\mathbf{m}_{si}\}, c = 32, N_{\text{head}} = 8):$

*# Input projections*

$\mathbf{m}_{si} \leftarrow \mathrm{LayerNorm}(\mathbf{m}_{si})$

$\mathbf{q}_{si}^h, \mathbf{k}_{si}^h, \mathbf{v}_{si}^h = \mathrm{LinearNoBias}(\mathbf{m}_{si})$ $\quad \mathbf{q}_{si}^h, \mathbf{k}_{si}^h, \mathbf{v}_{si}^h \in \mathbb{R}^c,\ h \in \{1, \dots, N_{\text{head}}\}$

$\mathbf{g}_{si}^h = \mathrm{sigmoid}\left(\mathrm{Linear}(\mathbf{m}_{si})\right)$ $\quad \mathbf{g}_{si}^h \in \mathbb{R}^c$

*# Attention*

$a_{sti}^h = \mathrm{softmax}_t \left( \frac{1}{\sqrt{c}}\, {\mathbf{q}_{si}^h}^\top \mathbf{k}_{ti}^h \right)$

$\mathbf{o}_{si}^h = \mathbf{g}_{si}^h \odot \sum_t a_{sti}^h \mathbf{v}_{st}^h$

*# Output projection*

$\tilde{\mathbf{m}}_{si} = \mathrm{Linear}\left(\mathrm{concat}_h(\mathbf{o}_{si}^h)\right)$ $\quad \tilde{\mathbf{m}}_{si} \in \mathbb{R}^{c_m}$

**return** $\{\tilde{\mathbf{m}}_{si}\}$

### 1.6.3 MSA transition

After row-wise and column-wise attention the MSA stack contains a 2-layer MLP as the transition layer (Suppl. Fig. 4 and Algorithm 9). The intermediate number of channels expands the original number of channels by a factor of 4.

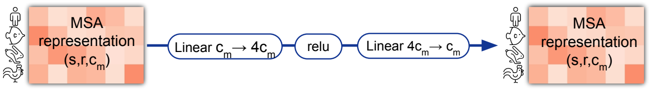


**Supplementary Figure 4** | MSA transition layer. Dimensions: s: sequences, r: residues, c: channels.

**Algorithm 9** Transition layer in the MSA stack

**def** $\mathrm{MSATransition}(\{\mathbf{m}_{si}\}, n = 4):$

$\mathbf{m}_{si} \leftarrow \mathrm{LayerNorm}(\mathbf{m}_{si})$

$\mathbf{a}_{si} = \mathrm{Linear}(\mathbf{m}_{si})$ $\qquad \mathbf{a}_{si} \in \mathbb{R}^{n \cdot c_m}$

$\mathbf{m}_{si} \leftarrow \mathrm{Linear}(\mathrm{relu}(\mathbf{a}_{si}))$

**return** $\{\mathbf{m}_{si}\}$

### 1.6.4 Outer product mean

The "Outer product mean" block transforms the MSA representation into an update for the pair representation (Suppl. Fig. 5 and Algorithm 10). All MSA entries are linearly projected to a smaller dimension $c = 32$ with two independent Linear transforms. The outer products of these vectors from two columns $i$ and $j$ are averaged over the sequences and projected to dimension $c_z$ to obtain an update for entry $ij$ in the pair representation. This is a memory intensive operation, as it requires constructing high-dimensional intermediate tensors. See section 1.11.8 for implementation details.

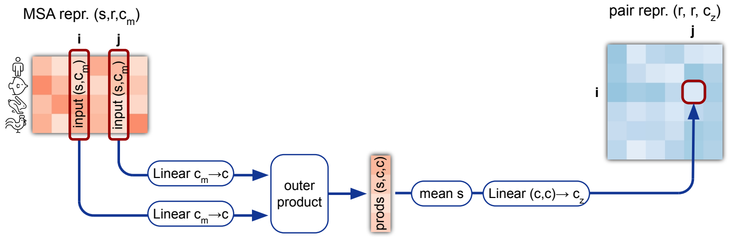


**Supplementary Figure 5** | Outer product mean. Dimensions: s: sequences, r: residues, c: channels.

**Algorithm 10** Outer product mean

**def** $\mathrm{OuterProductMean}(\{\mathbf{m}_{si}\}, c = 32):$

$\mathbf{m}_{si} \leftarrow \mathrm{LayerNorm}(\mathbf{m}_{si})$

$\mathbf{a}_{si}, \mathbf{b}_{si} = \mathrm{Linear}(\mathbf{m}_{si})$ $\qquad \mathbf{a}_{si}, \mathbf{b}_{si} \in \mathbb{R}^{c}$

$\mathbf{o}_{ij} = \mathrm{flatten}\left(\mathrm{mean}_s(\mathbf{a}_{si} \otimes \mathbf{b}_{sj})\right)$ $\qquad \mathbf{o}_{ij} \in \mathbb{R}^{c \cdot c}$

$\mathbf{z}_{ij} = \mathrm{Linear}(\mathbf{o}_{ij})$ $\qquad \mathbf{z}_{ij} \in \mathbb{R}^{c_z}$

**return** $\{\mathbf{z}_{ij}\}$

### 1.6.5 Triangular multiplicative update

The triangular multiplicative update (**Fig. 3c**) updates the pair representation in the Evoformer block by combining information within each triangle of graph edges $ij$, $ik$, and $jk$. Each edge $ij$ receives an update from the other two edges of all triangles, where it is involved. There are two symmetric versions, one for the "outgoing" edges (Suppl. Fig. 6 and Algorithm 11) and one for the "incoming" edges (Algorithm 12). The differences are highlighted in yellow.

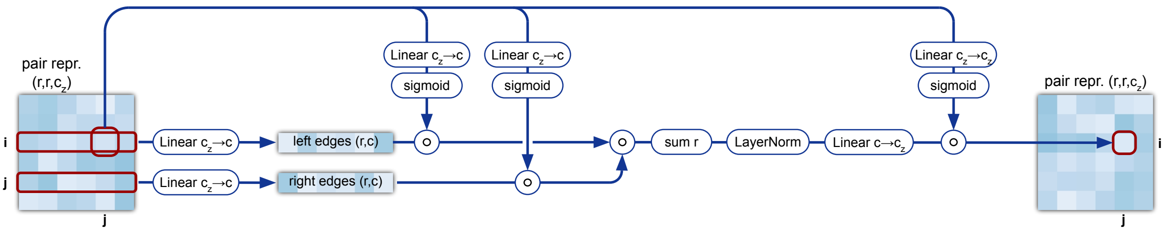


**Supplementary Figure 6** | Triangular multiplicative update using "outgoing" edges. Dimensions: r: residues, c: channels.

---

**Algorithm 11** Triangular multiplicative update using "outgoing" edges

---

**def** TriangleMultiplicationOutgoing($\{\mathbf{z}_{ij}\}, c = 128$) :

$\mathbf{z}_{ij} \leftarrow \mathrm{LayerNorm}(\mathbf{z}_{ij})$

$\mathbf{a}_{ij}, \mathbf{b}_{ij} = \mathrm{sigmoid}\left(\mathrm{Linear}(\mathbf{z}_{ij})\right) \odot \mathrm{Linear}(\mathbf{z}_{ij})$ $\qquad \mathbf{a}_{ij}, \mathbf{b}_{ij} \in \mathbb{R}^{c}$

$\mathbf{g}_{ij} = \mathrm{sigmoid}\left(\mathrm{Linear}(\mathbf{z}_{ij})\right)$ $\qquad \mathbf{g}_{ij} \in \mathbb{R}^{c_z}$

$\tilde{\mathbf{z}}_{ij} = \mathbf{g}_{ij} \odot \mathrm{Linear}(\mathrm{LayerNorm}(\sum_k \mathbf{a}_{ik} \odot \mathbf{b}_{jk}))$ $\qquad \tilde{\mathbf{z}}_{ij} \in \mathbb{R}^{c_z}$

**return** $\{\tilde{\mathbf{z}}_{ij}\}$

---

**Algorithm 12** Triangular multiplicative update using "incoming" edges

---

**def** TriangleMultiplicationIncoming($\{\mathbf{z}_{ij}\}, c = 128$) :

$\mathbf{z}_{ij} \leftarrow \mathrm{LayerNorm}(\mathbf{z}_{ij})$

$\mathbf{a}_{ij}, \mathbf{b}_{ij} = \mathrm{sigmoid}\left(\mathrm{Linear}(\mathbf{z}_{ij})\right) \odot \mathrm{Linear}(\mathbf{z}_{ij})$ $\qquad \mathbf{a}_{ij}, \mathbf{b}_{ij} \in \mathbb{R}^{c}$

$\mathbf{g}_{ij} = \mathrm{sigmoid}\left(\mathrm{Linear}(\mathbf{z}_{ij})\right)$ $\qquad \mathbf{g}_{ij} \in \mathbb{R}^{c_z}$

$\tilde{\mathbf{z}}_{ij} = \mathbf{g}_{ij} \odot \mathrm{Linear}(\mathrm{LayerNorm}(\sum_k \mathbf{a}_{ki} \odot \mathbf{b}_{kj}))$ $\qquad \tilde{\mathbf{z}}_{ij} \in \mathbb{R}^{c_z}$

**return** $\{\tilde{\mathbf{z}}_{ij}\}$

---

### 1.6.6 Triangular self-attention

The triangular self-attention (**Fig. 3c**) updates the pair representation in the Evoformer block. The "starting node" version (Suppl. Fig. 7 and Algorithm 13) updates the edge $ij$ with values from all edges that share the same starting node $i$, (i.e. all edges $ik$). The decision whether edge $ij$ will receive an update from edge $ik$ is not only determined by their query-key similarity (as in standard attention [95]), but also modulated by the information $b_{jk}$ derived from the third edge $jk$ of this triangle. Furthermore we extend the update with an additional gating $g_{ij}$ derived from edge $ij$. The symmetric pair of this module operates on the edges around the ending node (Algorithm 14). The differences are highlighted in yellow.

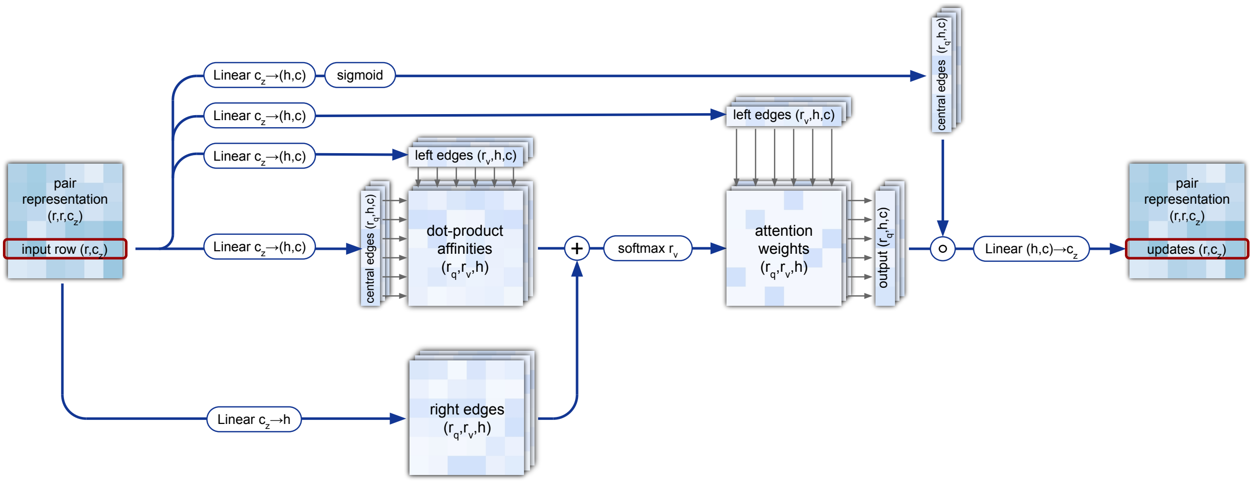


**Supplementary Figure 7** | Triangular self-attention around starting node. Dimensions: r: residues, c: channels, h: heads

---

**Algorithm 13** Triangular gated self-attention around starting node

---

**def** TriangleAttentionStartingNode($\{\mathbf{z}_{ij}\}, c = 32, N_{\text{head}} = 4$) :

*# Input projections*

$\mathbf{z}_{ij} \leftarrow \text{LayerNorm}(\mathbf{z}_{ij})$

$\mathbf{q}_{ij}^h, \mathbf{k}_{ij}^h, \mathbf{v}_{ij}^h = \text{LinearNoBias}(\mathbf{z}_{ij})$ $\quad\quad \mathbf{q}_{ij}^h, \mathbf{k}_{ij}^h, \mathbf{v}_{ij}^h \in \mathbb{R}^c,\ h \in \{1, \dots, N_{\text{head}}\}$

$b_{ij}^h = \text{LinearNoBias}(\mathbf{z}_{ij})$

$\mathbf{g}_{ij}^h = \text{sigmoid}\left(\text{Linear}(\mathbf{z}_{ij})\right)$ $\quad\quad \mathbf{g}_{ij}^h \in \mathbb{R}^c$

*# Attention*

$a_{ijk}^h = \text{softmax}_k\left(\frac{1}{\sqrt{c}}\, \mathbf{q}_{ij}^{h\top} \mathbf{k}_{ik}^h + b_{jk}^h\right)$

$\mathbf{o}_{ij}^h = \mathbf{g}_{ij}^h \odot \sum_k a_{ijk}^h \mathbf{v}_{ik}^h$

*# Output projection*

$\tilde{\mathbf{z}}_{ij} = \text{Linear}\left(\text{concat}_h(\mathbf{o}_{ij}^h)\right)$ $\quad\quad \tilde{\mathbf{z}}_{ij} \in \mathbb{R}^{c_z}$

**return** $\{\tilde{\mathbf{z}}_{ij}\}$

---

**Algorithm 14** Triangular gated self-attention around ending node

**def** $\text{TriangleAttentionEndingNode}(\{\mathbf{z}_{ij}\}, c = 32, N_{\text{head}} = 4):$

*# Input projections*

$\mathbf{z}_{ij} \leftarrow \text{LayerNorm}(\mathbf{z}_{ij})$

$\mathbf{q}_{ij}^h, \mathbf{k}_{ij}^h, \mathbf{v}_{ij}^h = \text{LinearNoBias}(\mathbf{z}_{ij})$ $\qquad \mathbf{q}_{ij}^h, \mathbf{k}_{ij}^h, \mathbf{v}_{ij}^h \in \mathbb{R}^c,\ h \in \{1, \dots, N_{\text{head}}\}$

$b_{ij}^h = \text{LinearNoBias}(\mathbf{z}_{ij})$

$\mathbf{g}_{ij}^h = \text{sigmoid}\left(\text{Linear}(\mathbf{z}_{ij})\right)$ $\qquad \mathbf{g}_{ij}^h \in \mathbb{R}^c$

*# Attention*

$a_{ijk}^h = \text{softmax}_k \left( \frac{1}{\sqrt{c}}\, \mathbf{q}_{ij}^{h\top} \mathbf{k}_{kj}^h + b_{ki}^h \right)$

$\mathbf{o}_{ij}^h = \mathbf{g}_{ij}^h \odot \sum_k a_{ijk}^h \mathbf{v}_{kj}^h$

*# Output projection*

$\tilde{\mathbf{z}}_{ij} = \text{Linear}\left(\text{concat}_h\left(\mathbf{o}_{ij}^h\right)\right)$ $\qquad \tilde{\mathbf{z}}_{ij} \in \mathbb{R}^{c_z}$

**return** $\{\tilde{\mathbf{z}}_{ij}\}$

### 1.6.7 Transition in the pair stack

The transition layer in the pair stack (Algorithm 15) is equivalent to that in the MSA stack: a 2-layer MLP where the intermediate number of channels expands the original number of channels by a factor of 4.

**Algorithm 15** Transition layer in the pair stack

**def** $\text{PairTransition}(\{\mathbf{z}_{ij}\}, n = 4):$

$\mathbf{z}_{ij} \leftarrow \text{LayerNorm}(\mathbf{z}_{ij})$

$\mathbf{a}_{ij} = \text{Linear}(\mathbf{z}_{ij})$ $\qquad \mathbf{a}_{ij} \in \mathbb{R}^{n \cdot c_z}$

$\mathbf{z}_{ij} \leftarrow \text{Linear}(\text{relu}(\mathbf{a}_{ij}))$

**return** $\{\mathbf{z}_{ij}\}$

## 1.7 Additional inputs

As outlined in Suppl. Fig. 1, the additional model inputs include templates and unclustered MSA sequences. In this section we explain how they are embedded in more detail.

### 1.7.1 Template stack

Pairwise template features are linearly projected to form initial template representations $\mathbf{t}_{s_t ij}$, with $\mathbf{t}_{s_t ij} \in \mathbb{R}^{c_t}$, $c_t = 64$, and $i, j \in \{1 \dots N_{\text{res}}\}$, $s_t \in \{1 \dots N_{\text{templ}}\}$. Each template representation is independently processed with the template pair stack (Algorithm 16) with all trainable parameters shared across templates. The output representations are aggregated with the template point-wise attention (Algorithm 17), where the pair representations $\{\mathbf{z}_{ij}\}$ are used to form the queries and attend over the individual templates. The outputs of this module are added to the pair representations (see Suppl. Fig. 1 or line 13 in Algorithm 2).

Furthermore, template torsion angle features are embedded with a small MLP and concatenated with the MSA representations as additional sequence rows (see Suppl. Fig. 1 or lines 7-8 in Algorithm 2). These additional rows are participating in all MSA stack operations, but not in the masked MSA loss (subsubsection 1.9.9). While template torsion angle and MSA features are conceptually different quantities, they are embedded with different sets of weights, thus the learning process presumably drives the embeddings to be comparable since they are processed by the same downstream modules with the same weights.

**Algorithm 16** Template pair stack

**def** $\text{TemplatePairStack}(\{\mathbf{t}_{ij}\}, N_{\text{block}} = 2):$

**for all** $l \in [1, \ldots, N_{\text{block}}]$ **do**

  $\{\mathbf{t}_{ij}\} \mathrel{+}= \text{DropoutRowwise}_{0.25}(\text{TriangleAttentionStartingNode}(\{\mathbf{t}_{ij}\}, c = 64, N_{\text{head}} = 4))$

  $\{\mathbf{t}_{ij}\} \mathrel{+}= \text{DropoutColumnwise}_{0.25}(\text{TriangleAttentionEndingNode}(\{\mathbf{t}_{ij}\}, c = 64, N_{\text{head}} = 4))$

  $\{\mathbf{t}_{ij}\} \mathrel{+}= \text{DropoutRowwise}_{0.25}(\text{TriangleMultiplicationOutgoing}(\{\mathbf{t}_{ij}\}, c = 64))$

  $\{\mathbf{t}_{ij}\} \mathrel{+}= \text{DropoutRowwise}_{0.25}(\text{TriangleMultiplicationIncoming}(\{\mathbf{t}_{ij}\}, c = 64))$

  $\{\mathbf{t}_{ij}\} \mathrel{+}= \text{PairTransition}(\{\mathbf{t}_{ij}\}, n = 2)$

**end for**

**return** $\text{LayerNorm}\left(\{\mathbf{t}_{ij}\}\right)$

**Algorithm 17** Template pointwise attention

**def** $\text{TemplatePointwiseAttention}(\{\mathbf{t}_{s_t ij}\}, \{\mathbf{z}_{ij}\}, c = 64, N_{\text{head}} = 4):$

$\mathbf{q}_{ij}^h = \text{LinearNoBias}(\mathbf{z}_{ij})$  $\mathbf{q}_{ij}^h \in \mathbb{R}^c,\ h \in \{1, \ldots, N_{\text{head}}\}$

$\mathbf{k}_{s_t ij}^h, \mathbf{v}_{s_t ij}^h = \text{LinearNoBias}(\mathbf{t}_{s_t ij})$  $\mathbf{k}_{s_t ij}^h, \mathbf{v}_{s_t ij}^h \in \mathbb{R}^c$

$a_{s_t ij}^h = \text{softmax}_{s_t}\left(\frac{1}{\sqrt{c}}\, \mathbf{q}_{ij}^{h\top} \mathbf{k}_{s_t ij}^h\right)$

$\mathbf{o}_{ij}^h = \sum_{s_t} a_{s_t ij}^h \mathbf{v}_{s_t ij}^h$

$\{\tilde{\mathbf{z}}_{ij}\} = \text{Linear}\left(\text{concat}_h(\{\mathbf{o}_{ij}^h\})\right)$  $\tilde{\mathbf{z}}_{ij} \in \mathbb{R}^{c_z}$

**return** $\{\tilde{\mathbf{z}}_{ij}\}$

### 1.7.2 Unclustered MSA stack

Unclustered MSA sequence features are linearly projected to form initial representations $\{\mathbf{e}_{s_e i}\}$ with $\mathbf{e}_{s_e i} \in \mathbb{R}^{c_e}$, $c_e = 64$, and $s_e \in \{1 \ldots N_{\text{extra\_seq}}\}$, $i \in \{1 \ldots N_{\text{res}}\}$. These representations are processed with the Extra MSA stack containing 4 blocks (Algorithm 18). They are highly similar to the main Evoformer blocks, with the notable difference of using global column-wise self-attention (Algorithm 19) and smaller representation sizes to allow processing of the large number of sequences. The final pair representations are used as inputs for the main Evoformer stack (Algorithm 6), while the final MSA activations are unused (see Suppl. Fig. 1).

**Algorithm 18** Extra MSA stack

**def** ExtraMsaStack$(\{\mathbf{e}_{s_e i}\}, \{\mathbf{z}_{ij}\}, N_{\text{block}} = 4)$ :

**for all** $l \in [1, \ldots, N_{\text{block}}]$ **do**

*# MSA stack*

$\{\mathbf{e}_{s_e i}\}$ += DropoutRowwise$_{0.15}$(MSARowAttentionWithPairBias$(\{\mathbf{e}_{s_e i}\}, \{\mathbf{z}_{ij}\}, c = 8)$)

$\{\mathbf{e}_{s_e i}\}$ += MSAColumnGlobalAttention$(\{\mathbf{e}_{s_e i}\}$

$\{\mathbf{e}_{s_e i}\}$ += MSATransition$(\{\mathbf{e}_{s_e i}\})$

*# Communication*

$\{\mathbf{z}_{ij}\}$ += OuterProductMean$(\{\mathbf{e}_{s_e i}\})$

*# Pair stack*

$\{\mathbf{z}_{ij}\}$ += DropoutRowwise$_{0.25}$(TriangleMultiplicationOutgoing$(\{\mathbf{z}_{ij}\})$)

$\{\mathbf{z}_{ij}\}$ += DropoutRowwise$_{0.25}$(TriangleMultiplicationIncoming$(\{\mathbf{z}_{ij}\})$)

$\{\mathbf{z}_{ij}\}$ += DropoutRowwise$_{0.25}$(TriangleAttentionStartingNode$(\{\mathbf{z}_{ij}\})$)

$\{\mathbf{z}_{ij}\}$ += DropoutColumnwise$_{0.25}$(TriangleAttentionEndingNode$(\{\mathbf{z}_{ij}\})$)

$\{\mathbf{z}_{ij}\}$ += PairTransition$(\{\mathbf{z}_{ij}\})$

**end for**

**return** $\{\mathbf{z}_{ij}\}$

**Algorithm 19** MSA global column-wise gated self-attention

**def** MSAColumnGlobalAttention$(\{\mathbf{m}_{si}\}, c = 8, N_{\text{head}} = 8)$ :

*# Input projections*

$\mathbf{m}_{si} \leftarrow \text{LayerNorm}(\mathbf{m}_{si})$

$\mathbf{q}_{si}^h, \mathbf{k}_{si}, \mathbf{v}_{si} = \text{LinearNoBias}(\mathbf{m}_{si})$ $\quad \mathbf{q}_{si}^h, \mathbf{k}_{si}, \mathbf{v}_{si} \in \mathbb{R}^c,\ h \in \{1, \ldots, N_{\text{head}}\}$

$\mathbf{q}_i^h = \text{mean}_s\, \mathbf{q}_{si}^h$

$\mathbf{g}_{si}^h = \text{sigmoid}\left(\text{Linear}(\mathbf{m}_{si})\right)$ $\quad \mathbf{g}_{si}^h \in \mathbb{R}^c$

*# Attention*

$a_{ti}^h = \text{softmax}_t \left( \frac{1}{\sqrt{c}}\, \mathbf{q}_i^{h\top} \mathbf{k}_{ti} \right)$

$\mathbf{o}_{si}^h = \mathbf{g}_{si}^h \odot \sum_t a_{ti}^h \mathbf{v}_{ti}$

*# Output projection*

$\tilde{\mathbf{m}}_{si} = \text{Linear}\left(\text{concat}_h\left(\mathbf{o}_{si}^h\right)\right)$ $\quad \tilde{\mathbf{m}}_{si} \in \mathbb{R}^{c_m}$

**return** $\{\tilde{\mathbf{m}}_{si}\}$

## 1.8 Structure module

The Structure Module (Algorithm 20 and **Fig. 3d** in the main article) maps the abstract representation of the protein structure (created by the Evoformer stack) to concrete 3D atom coordinates. Evoformer's single representation is used as the initial single representation $\{\mathbf{s}_i^{\text{initial}}\}$ with $\mathbf{s}_i^{\text{initial}} \in \mathbb{R}^{c_s}$ and Evoformer's pair representation $\{\mathbf{z}_{ij}\}$ with $\mathbf{z}_{ij} \in \mathbb{R}^{c_z}$ and $i, j \in \{1, ..., N_{\text{res}}\}$ biases the affinity maps in the attention operations. The module has 8 layers with shared weights. Each layer updates the abstract single representation $\{\mathbf{s}_i\}$ as well the concrete 3D representation (the "residue gas") which is encoded as one backbone frame per residue $\{T_i\}$. We represent frames via a tuple $T_i := (R_i, \vec{\mathbf{t}}_i)$. The tuple represents an Euclidean transform from the local frame to a global reference frame. I.e. it transforms a position in local coordinates $\vec{\mathbf{x}}_{\text{local}} \in \mathbb{R}^3$ to a position in global coordinates $\vec{\mathbf{x}}_{\text{global}} \in \mathbb{R}^3$ as

$$\begin{aligned} \vec{\mathbf{x}}_{\text{global}} &= T_i \circ \vec{\mathbf{x}}_{\text{local}} \\ &= R_i \vec{\mathbf{x}}_{\text{local}} + \vec{\mathbf{t}}_i \ . \end{aligned} \tag{2}$$

The backbone frames are initialized to an identity transform (Algorithm 20 line 4). We call this approach the 'black hole initialization'. This initialization means that at the start of the Structure module all residues are located at the same point (the origin of the global frame) with the same orientation. One "layer" of the structure module is composed by the following operations: First, the abstract single representation is updated by the Invariant Point Attention (Algorithm 20 line 6) (see subsubsection 1.8.2 for details) and a transition layer. Then the abstract single representation is mapped to concrete update frames that are composed to the backbone frames (Algorithm 20 line 10). The composition of two Euclidean transforms is denoted as

$$T_{\text{result}} = T_1 \circ T_2$$

In the parameterization with a rotation matrix and translation vector this is:

$$\begin{aligned} (R_{\text{result}}, \vec{\mathbf{t}}_{\text{result}}) &= (R_1, \vec{\mathbf{t}}_1) \circ (R_2, \vec{\mathbf{t}}_2) \\ &= (R_1 R_2, \ \ R_1 \vec{\mathbf{t}}_2 + \vec{\mathbf{t}}_1) \end{aligned}$$

To obtain all atom coordinates, we parameterize each residue by torsion angles. I.e., the torsion angles are the only degrees of freedom, while all bond angles and bond lengths are fully rigid. The individual atoms are identified by their names $\mathcal{S}_{\text{atom names}} = \{\text{N}, \text{C}^\alpha, \text{C}, \text{O}, \text{C}^\beta, \text{C}^\gamma, \text{C}^{\gamma 1}, \text{C}^{\gamma 2}, \dots\}$. The torsions are named as $\mathcal{S}_{\text{torsion names}} = \{\omega, \phi, \psi, \chi_1, \chi_2, \chi_3, \chi_4\}$ We group the atoms according to their dependence on the torsion angles into "rigid groups" (Table 2). E.g., the position of atoms in the $\chi_2$-group depend on $\chi_1$ and $\chi_2$, but do not depend on $\chi_3$ or $\chi_4$. We define a rigid group for every of the 3 backbone and 4 side chain torsion angles $\omega, \phi, \psi, \chi_1, \chi_2, \chi_3, \chi_4$, though with our construction of the backbone frames, no heavy atoms positions depend on the torsion angles $\omega$ or $\phi$. The $\chi_5$ angle is not included, because it only appears in arginine and only slightly varies around 0 degrees. Atoms that just depend on the backbone frame are in the "backbone rigid group", denoted as "bb".

**Table 2** | Rigid groups for constructing all atoms from given torsion angles. Boxes highlight groups that are symmetric under 180° rotations.

| aatype | bb | $\psi$ | $\chi_1$ | $\chi_2$ | $\chi_3$ | $\chi_4$ |
|---|---|---|---|---|---|---|
| ALA | N, $C^\alpha$, C, $C^\beta$ | O | - | - | - | - |
| ARG | N, $C^\alpha$, C, $C^\beta$ | O | $C^\gamma$ | $C^\delta$ | $N^\epsilon$ | $N^{\eta 1}$, $N^{\eta 2}$, $C^\zeta$ |
| ASN | N, $C^\alpha$, C, $C^\beta$ | O | $C^\gamma$ | $N^{\delta 2}$, $O^{\delta 1}$ | - | - |
| ASP | N, $C^\alpha$, C, $C^\beta$ | O | $C^\gamma$ | $\boxed{O^{\delta 1}, O^{\delta 2}}$ | - | - |
| CYS | N, $C^\alpha$, C, $C^\beta$ | O | $S^\gamma$ | - | - | - |
| GLN | N, $C^\alpha$, C, $C^\beta$ | O | $C^\gamma$ | $C^\delta$ | $N^{\epsilon 2}$, $O^{\epsilon 1}$ | - |
| GLU | N, $C^\alpha$, C, $C^\beta$ | O | $C^\gamma$ | $C^\delta$ | $\boxed{O^{\epsilon 1}, O^{\epsilon 2}}$ | - |
| GLY | N, $C^\alpha$, C | O | - | - | - | - |
| HIS | N, $C^\alpha$, C, $C^\beta$ | O | $C^\gamma$ | $C^{\delta 2}$, $N^{\delta 1}$, $C^{\epsilon 1}$, $N^{\epsilon 2}$ | - | - |
| ILE | N, $C^\alpha$, C, $C^\beta$ | O | $C^{\gamma 1}$, $C^{\gamma 2}$ | $C^{\delta 1}$ | - | - |
| LEU | N, $C^\alpha$, C, $C^\beta$ | O | $C^\gamma$ | $C^{\delta 1}$, $C^{\delta 2}$ | - | - |
| LYS | N, $C^\alpha$, C, $C^\beta$ | O | $C^\gamma$ | $C^\delta$ | $C^\epsilon$ | $N^\zeta$ |
| MET | N, $C^\alpha$, C, $C^\beta$ | O | $C^\gamma$ | $S^\delta$ | $C^\epsilon$ | - |
| PHE | N, $C^\alpha$, C, $C^\beta$ | O | $C^\gamma$ | $\boxed{C^{\delta 1}, C^{\delta 2}, C^{\epsilon 1}, C^{\epsilon 2}, C^\zeta}$ | - | - |
| PRO | N, $C^\alpha$, C, $C^\beta$ | O | $C^\gamma$ | $C^\delta$ | - | - |
| SER | N, $C^\alpha$, C, $C^\beta$ | O | $O^\gamma$ | - | - | - |
| THR | N, $C^\alpha$, C, $C^\beta$ | O | $C^{\gamma 2}$, $O^{\gamma 1}$ | - | - | - |
| TRP | N, $C^\alpha$, C, $C^\beta$ | O | $C^\gamma$ | $C^{\delta 1}$, $C^{\delta 2}$, $C^{\epsilon 2}$, $C^{\epsilon 3}$, $N^{\epsilon 1}$, $C^{\eta 2}$, $C^{\zeta 2}$, $C^{\zeta 3}$ | - | - |
| TYR | N, $C^\alpha$, C, $C^\beta$ | O | $C^\gamma$ | $\boxed{C^{\delta 1}, C^{\delta 2}, C^{\epsilon 1}, C^{\epsilon 2}, O^\eta, C^\zeta}$ | - | - |
| VAL | N, $C^\alpha$, C, $C^\beta$ | O | $C^{\gamma 1}$, $C^{\gamma 2}$ | - | - | - |

A shallow ResNet (Algorithm 20 line 11) predicts the torsion angles $\vec{\alpha}_i^f \in \mathbb{R}^2$. They get mapped to points on the unit circle via normalization whenever they are used as angles. Furthermore we introduce a small auxiliary loss (implemented in Algorithm 27) that encourages a unit norm of the raw vector to avoid degenerate values. In contrast to a $[0, 2\pi]$ angle representation this representation has no discontinuity and can be directly used to construct rotation matrices without the need for trigonometric functions. The predicted torsion angles are converted to frames for the rigid groups of atoms (see subsubsection 1.8.4 for details).

During training, the last step of each layer computes auxiliary losses for the current 3D structure (Algorithm 20 line 17). The intermediate FAPE loss operates only on the backbone frames and $C^\alpha$ atom positions to keep the computational costs low. For the same reason the side chain are here only supervised by their torsion angles. The 180° rotation symmetry of some rigid groups (highlighted by boxes in Table 2) is addressed by providing the alternative angle $\vec{\alpha}_i^{\text{alt truth},f}$ as well (see subsubsection 1.9.1 for details).

We found it helpful to zero the gradients into the orientation component of the rigid bodies between iterations (Algorithm 20 line 20), so any iteration is optimized to find an optimal orientation for the structure in the current iteration, but is not concerned by having an orientation more suitable for the next iteration. Empirically, this improves the stability of training, presumably by removing the lever effects arising in a chained composition frames. After the 8 layers, the final backbone frames and torsion angles are mapped to frames for all rigid groups (backbone and side chain) $T_i^f$ and all atom coordinates $\vec{\mathbf{x}}_i^a$ (Algorithm 20 line 24).

During training, the predicted frames and atom coordinates are compared against the ground truth by the FAPE loss (Algorithm 20 line 28) that assesses all atom coordinates (backbone and side chain) relative to all rigid groups. The 180°-rotation-symmetries of a few rigid groups are handled by a globally consistent renaming of the ambiguous atoms in the ground truth structure (see subsubsection 1.8.5 for details).

Finally the model predicts its confidence in form of a predicted lDDT-C$\alpha$ score (pLDDT) per residue. This score is trained with the true per-residue lDDT-C$\alpha$ score computed from the predicted structure and the ground truth. For details see subsubsection 1.9.6.

**Algorithm 20** Structure module

**def** StructureModule $\left(\{\mathbf{s}_i^{\text{initial}}\}, \{\mathbf{z}_{ij}\}, N_{\text{layer}} = 8, c = 128,\right.$ $\qquad \mathbf{s}_i^{\text{initial}} \in \mathbb{R}^{c_s}$

$\left.\{T_i^{\text{true},f}\}, \{T_i^{\text{alt truth},f}\}, \{\vec{\alpha}_i^{\text{true},f}\}, \{\vec{\alpha}_i^{\text{alt truth},f}\}, \{\vec{\mathbf{x}}_i^{\text{true},a}\}, \{\vec{\mathbf{x}}_i^{\text{alt truth},a}\}\right)$ :

$\mathbf{s}_i^{\text{initial}} \leftarrow \text{LayerNorm}(\mathbf{s}_i^{\text{initial}})$

$\mathbf{z}_{ij} \leftarrow \text{LayerNorm}(\mathbf{z}_{ij})$

$\mathbf{s}_i = \text{Linear}(\mathbf{s}_i^{\text{initial}})$ $\qquad \mathbf{s}_i \in \mathbb{R}^{c_s}$

$T_i = (\mathbf{I}, \vec{\mathbf{0}})$ $\qquad \mathbf{I} \in \mathbb{R}^{3\times 3}, \vec{\mathbf{0}} \in \mathbb{R}^3$

**for all** $l \in [1, \dots, N_{\text{layer}}]$ **do** *# shared weights*

$\quad \{\mathbf{s}_i\}$ += InvariantPointAttention$(\{\mathbf{s}_i\}, \{\mathbf{z}_{ij}\}, \{T_i\})$

$\quad \mathbf{s}_i \leftarrow \text{LayerNorm}(\text{Dropout}_{0.1}(\mathbf{s}_i))$

*# Transition.*

$\quad \mathbf{s}_i \leftarrow \mathbf{s}_i + \text{Linear}(\text{relu}(\text{Linear}(\text{relu}(\text{Linear}(\mathbf{s}_i)))))$ $\qquad$ all intermediate activations $\in \mathbb{R}^{c_s}$

$\quad \mathbf{s}_i \leftarrow \text{LayerNorm}(\text{Dropout}_{0.1}(\mathbf{s}_i))$

*# Update backbone.*

$\quad T_i \leftarrow T_i \circ$ BackboneUpdate$(\mathbf{s}_i)$

*# Predict side chain and backbone torsion angles $\omega, \phi, \psi, \chi_1, \chi_2, \chi_3, \chi_4$*

$\quad \mathbf{a}_i = \text{Linear}(\mathbf{s}_i) + \text{Linear}(\mathbf{s}_i^{\text{initial}})$ $\qquad \mathbf{a}_i \in \mathbb{R}^{c}$

$\quad \mathbf{a}_i \leftarrow \mathbf{a}_i + \text{Linear}(\text{relu}(\text{Linear}(\text{relu}(\mathbf{a}_i))))$ $\qquad$ all intermediate activations $\in \mathbb{R}^{c}$

$\quad \mathbf{a}_i \leftarrow \mathbf{a}_i + \text{Linear}(\text{relu}(\text{Linear}(\text{relu}(\mathbf{a}_i))))$ $\qquad$ all intermediate activations $\in \mathbb{R}^{c}$

$\quad \vec{\alpha}_i^f = \text{Linear}(\text{relu}(\mathbf{a}_i))$ $\qquad \vec{\alpha}_i^f \in \mathbb{R}^2, f \in \mathcal{S}_{\text{torsion names}}$

*# Auxiliary losses in every iteration.*

$\quad (R_i, \vec{\mathbf{t}}_i) = T_i$

$\quad \vec{\mathbf{x}}_i^{\text{C}\alpha} = \vec{\mathbf{t}}_i$

$\quad \mathcal{L}_{\text{aux}}^l = \Big($computeFAPE$(\{T_i\}, \{\vec{\mathbf{x}}_i^{\text{C}\alpha}\}, \{T_i^{\text{true}}\}, \{\vec{\mathbf{x}}_i^{\text{true,C}\alpha}\}, \epsilon = 10^{-12})$

$\qquad\qquad +$ torsionAngleLoss$(\{\vec{\alpha}_i^f\}, \{\vec{\alpha}_i^{\text{true},f}\}, \{\vec{\alpha}_i^{\text{alt truth},f}\})\Big)$

*# No rotation gradients between iterations to stabilize training.*

$\quad$ **if** $l < N_{\text{layer}}$ **then**

$\qquad T_i \leftarrow (\text{stopgrad}(R_i), \vec{\mathbf{t}}_i)$

$\quad$ **end if**

**end for**

$\mathcal{L}_{\text{aux}} = \text{mean}_l(\{\mathcal{L}_{\text{aux}}^l\})$

$T_i^f, \vec{\mathbf{x}}_i^a =$ computeAllAtomCoordinates$(T_i, \vec{\alpha}_i^f)$ $\qquad a \in \mathcal{S}_{\text{atom names}}$

$T_i^f \leftarrow \text{concat}(T_i, T_i^f)$

*# Final loss on all atom coordinates.*

$\{T_i^{\text{true},f}\}, \{\vec{\mathbf{x}}_i^{\text{true},a}\} \leftarrow$ renameSymmetricGroundTruthAtoms(

$\qquad \{T_i^f\}, \{\vec{\mathbf{x}}_i^a\}, \{T_i^{\text{true},f}\}, \{T_i^{\text{alt truth},f}\}, \{\vec{\mathbf{x}}_i^{\text{true},a}\}, \{\vec{\mathbf{x}}_i^{\text{alt truth},a}\})$

$\mathcal{L}_{\text{FAPE}} =$ computeFAPE$(\{T_i^f\}, \{\vec{\mathbf{x}}_i^a\}, \{T_i^{\text{true},f}\}, \{\vec{\mathbf{x}}_i^{\text{true},a}\}, \epsilon = 10^{-4})$

*# Predict model confidence.*

$\{r_i^{\text{true LDDT}}\} = \text{perResidueLDDT\_Ca}(\{\vec{\mathbf{x}}_i^a\}, \{\vec{\mathbf{x}}_i^{\text{true},a}\})$

$\{r_i^{\text{pLDDT}}\}, \mathcal{L}_{\text{conf}} =$ predictPerResidueLDDT_Ca$(\{\mathbf{s}_i\}, \{r_i^{\text{true LDDT}}\})$

**return** $\{\vec{\mathbf{x}}_i^a\}, \{r_i^{\text{pLDDT}}\}, \mathcal{L}_{\text{FAPE}}, \mathcal{L}_{\text{conf}}, \mathcal{L}_{\text{aux}}$

### 1.8.1 Construction of frames from ground truth atom positions

We construct frames using the position of three atoms from the ground truth PDB structures using a Gram–Schmidt process (Algorithm 21). Note that the translation vector $\vec{\mathbf{t}}$ is assigned to the centre atom $\vec{\mathbf{x}}_2$. For backbone frames, we use N as $\vec{\mathbf{x}}_1$, C$\alpha$ as $\vec{\mathbf{x}}_2$ and C as $\vec{\mathbf{x}}_3$, so the frame has C$\alpha$ at the centre. For the side chain frames, we use the atom before the torsion bond as $\vec{\mathbf{x}}_1$, the atom after the torsion bond as $\vec{\mathbf{x}}_2$ and the next atom after that as $\vec{\mathbf{x}}_3$.

**Algorithm 21** Rigid from 3 points using the Gram–Schmidt process

**def** rigidFrom3Points$(\vec{\mathbf{x}}_1, \vec{\mathbf{x}}_2, \vec{\mathbf{x}}_3)$ : $\quad \vec{\mathbf{x}}_1, \vec{\mathbf{x}}_2, \vec{\mathbf{x}}_3 \in \mathbb{R}^3$

$\vec{\mathbf{v}}_1 = \vec{\mathbf{x}}_3 - \vec{\mathbf{x}}_2$

$\vec{\mathbf{v}}_2 = \vec{\mathbf{x}}_1 - \vec{\mathbf{x}}_2$

$\vec{\mathbf{e}}_1 = \vec{\mathbf{v}}_1 / \left\|\vec{\mathbf{v}}_1\right\|$

$\vec{\mathbf{u}}_2 = \vec{\mathbf{v}}_2 - \vec{\mathbf{e}}_1 \left(\vec{\mathbf{e}}_1^\top \vec{\mathbf{v}}_2\right)$

$\vec{\mathbf{e}}_2 = \vec{\mathbf{u}}_2 / \left\|\vec{\mathbf{u}}_2\right\|$

$\vec{\mathbf{e}}_3 = \vec{\mathbf{e}}_1 \times \vec{\mathbf{e}}_2$

$R = \mathrm{concat}(\vec{\mathbf{e}}_1, \vec{\mathbf{e}}_2, \vec{\mathbf{e}}_3)$ $\quad R \in \mathbb{R}^{3\times 3}$

$\vec{\mathbf{t}} = \vec{\mathbf{x}}_2$

**return** $(R, \vec{\mathbf{t}})$

### 1.8.2 Invariant point attention (IPA)

Invariant Point Attention (IPA) (Suppl. Fig. 8 and Algorithm 22) is a form of attention that acts on a set of frames (parametrized as Euclidean transforms $T_i$) and is invariant under global Euclidean transformations $T_{\text{global}}$ on said frames. We represent all the coordinates within IPA in nanometres; the choice of units affects the scaling of the point component of the attention affinities.

To define the initial weightings for the different terms, we assume that all queries and keys come iid from a unit normal distribution $N(0, 1)$ and compute the variances of the attention logits. Each scalar pair $q, k$ contributes $\mathrm{Var}[qk] = 1$. Each point pair $(\vec{\mathbf{q}}, \vec{\mathbf{k}})$ contributes $\mathrm{Var}[0.5 \left\|\vec{\mathbf{q}}\right\|^2 - \vec{\mathbf{q}}^\top \vec{\mathbf{k}}] = 9/2$. The weighting factors $w_L$ and $w_C$ are computed such that all three terms contribute equally and that the resulting variance is 1. The weight per head $\gamma^h \in \mathbb{R}$ is the softplus of a learnable scalar.

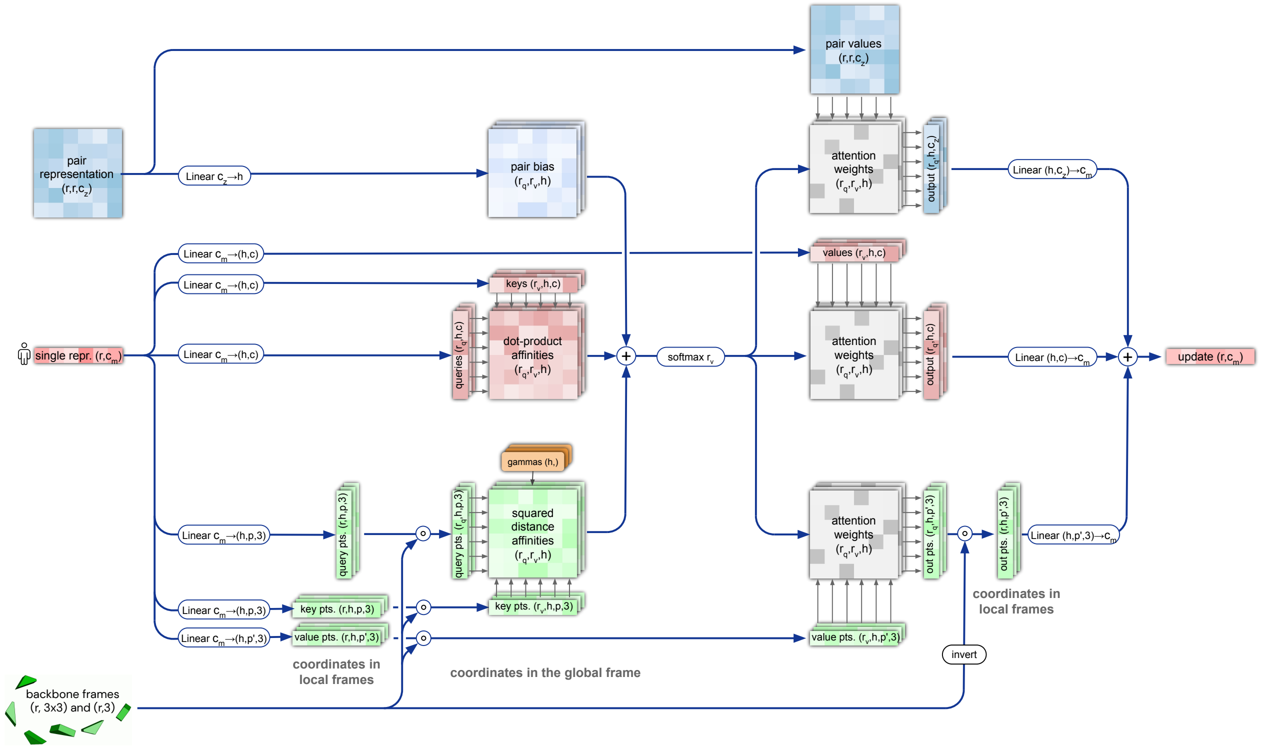


**Supplementary Figure 8** | Invariant Point Attention Module. (**top, blue arrays**) modulation by the pair representation. (**middle, red arrays**) standard attention on abstract features. (**bottom, green arrays**) Invariant point attention. Dimensions: r: residues, c: channels, h: heads, p: points.

**Algorithm 22** Invariant point attention (IPA)

**def** $\mathrm{InvariantPointAttention}(\{\mathbf{s}_i\}, \{\mathbf{z}_{ij}\}, \{T_i\}, N_{\text{head}} = 12, c = 16, N_{\text{query points}} = 4, N_{\text{point values}} = 8)$ :

1: $\mathbf{q}_i^h, \mathbf{k}_i^h, \mathbf{v}_i^h = \mathrm{LinearNoBias}(\mathbf{s}_i)$ $\qquad \mathbf{q}_i^h, \mathbf{k}_i^h, \mathbf{v}_i^h \in \mathbb{R}^c,\ h \in \{1, \dots, N_{\text{head}}\}$

2: $\vec{\mathbf{q}}_i^{hp}, \vec{\mathbf{k}}_i^{hp} = \mathrm{LinearNoBias}(\mathbf{s}_i)$ $\qquad \vec{\mathbf{q}}_i^{hp}, \vec{\mathbf{k}}_i^{hp}, \in \mathbb{R}^3,\ p \in \{1, \dots, N_{\text{query points}}\}$, units: nanometres

3: $\vec{\mathbf{v}}_i^{hp} = \mathrm{LinearNoBias}(\mathbf{s}_i)$ $\qquad \vec{\mathbf{v}}_i^{hp} \in \mathbb{R}^3,\ p \in \{1, \dots, N_{\text{point values}}\}$, units: nanometres

4: $b_{ij}^h = \mathrm{LinearNoBias}(\mathbf{z}_{ij})$

5: $w_C = \sqrt{\frac{2}{9 N_{\text{query points}}}},$

6: $w_L = \sqrt{\frac{1}{3}}$

7: $a_{ij}^h = \mathrm{softmax}_j\left(w_L\left(\frac{1}{\sqrt{c}}\, \mathbf{q}_i^{h\top} \mathbf{k}_j^h \ + b_{ij}^h \ - \frac{\gamma^h w_C}{2} \sum_p \left\| T_i \circ \vec{\mathbf{q}}_i^{hp} - T_j \circ \vec{\mathbf{k}}_j^{hp} \right\|^2\right)\right)$

8: $\tilde{\mathbf{o}}_i^h = \sum_j a_{ij}^h \mathbf{z}_{ij}$

9: $\mathbf{o}_i^h = \sum_j a_{ij}^h \mathbf{v}_j^h$

10: $\vec{\mathbf{o}}_i^{hp} = T_i^{-1} \circ \sum_j a_{ij}^h \left(T_j \circ \vec{\mathbf{v}}_j^{hp}\right)$

11: $\tilde{\mathbf{s}}_i = \mathrm{Linear}\left(\mathrm{concat}_{h,p}(\tilde{\mathbf{o}}_i^h, \mathbf{o}_i^h, \vec{\mathbf{o}}_i^{hp}, \left\|\vec{\mathbf{o}}_i^{hp}\right\|)\right)$

12: **return** $\{\tilde{\mathbf{s}}_i\}$

The proof for invariance is straight-forward: The global transformation cancels out in the affinity computation (Algorithm 22 line 7), because the L2-norm of a vector is invariant under rigid transformations:

$$\left\|\left(T_{\text{global}} \circ T_i\right) \circ \vec{\mathbf{q}}_i^{hp} - \left(T_{\text{global}} \circ T_j\right) \circ \vec{\mathbf{k}}_j^{hp}\right\|^2 = \left\|T_{\text{global}} \circ \left(T_i \circ \vec{\mathbf{q}}_i^{hp} - T_j \circ \vec{\mathbf{k}}_j^{hp}\right)\right\|^2 \tag{3}$$

$$= \left\|T_i \circ \vec{\mathbf{q}}_i^{hp} - T_j \circ \vec{\mathbf{k}}_j^{hp}\right\|^2 . \tag{4}$$

In the computation of the output points (Algorithm 22 line 10) it cancels out when mapping back to the local frame:

$$\left(T_{\text{global}} \circ T_i\right)^{-1} \circ \sum_j a_{ij}^h \left(\left(T_{\text{global}} \circ T_j\right) \circ \vec{\mathbf{v}}_j^{hp}\right) = T_i^{-1} \circ T_{\text{global}}^{-1} \circ T_{\text{global}} \circ \sum_j a_{ij}^h \left(T_j \circ \vec{\mathbf{v}}_j^{hp}\right) \tag{5}$$

$$= T_i^{-1} \circ \sum_j a_{ij}^h \left(T_j \circ \vec{\mathbf{v}}_j^{hp}\right) \tag{6}$$

The invariance with respect to the global reference frame in turn implies that applying a shared rigid motion to all residues, while keeping the embeddings fixed, will lead to the same update in the local frames. Therefore, the updated structure will be transformed by the same shared rigid motion showing that this update rule is equivariant under rigid motions. Here and elsewhere, “rigid motion” includes proper rotation and translation but not reflection.

### 1.8.3 Backbone update

The updates for the backbone frames are created by predicting a quaternion for the rotation and a vector for the translation (Algorithm 23). The first component of the non-unit quaternion is fixed to 1. The three components

defining the Euler axis are predicted by the network. This procedure guarantees a valid normalized quaternion and furthermore favours small rotations over large rotations (quaternion (1,0,0,0) is the identity rotation).

**Algorithm 23** Backbone update

**def** $\mathrm{BackboneUpdate}(\mathbf{s}_i)$ :

$b_i, c_i, d_i, \vec{\mathbf{t}}_i = \mathrm{Linear}(\mathbf{s}_i)$ $\quad b_i, c_i, d_i \in \mathbb{R},\ \vec{\mathbf{t}}_i \in \mathbb{R}^3$

*# Convert (non-unit) quaternion to rotation matrix.*

$(a_i, b_i, c_i, d_i) \leftarrow (1, b_i, c_i, d_i)/\sqrt{1 + b_i^2 + c_i^2, +d_i^2}$

$$R_i = \begin{pmatrix} a_i^2 + b_i^2 - c_i^2 - d_i^2 & 2b_ic_i - 2a_id_i & 2b_id_i + 2a_ic_i \\ 2b_ic_i + 2a_id_i & a_i^2 - b_i^2 + c_i^2 - d_i^2 & 2c_id_i - 2a_ib_i \\ 2b_id_i - 2a_ic_i & 2c_id_i + 2a_ib_i & a_i^2 - b_i^2 - c_i^2 + d_i^2 \end{pmatrix}$$

$T_i = (R_i, \vec{\mathbf{t}}_i)$

**return** $T_i$

### 1.8.4 Compute all atom coordinates

The Structure Module predicts backbone frames $T_i$ and torsion angles $\vec{\alpha}_i^f$. The atom coordinates are then constructed by applying the torsion angles to the corresponding amino acid structure with idealized bond angles and bond lengths. We attach a local frame to each rigid group (see Table 2), such that the torsion axis is the x-axis, and store the ideal literature atom coordinates [98] for each amino acid relative to these frames in a table $\vec{\mathbf{x}}_{r,f,a}^{\mathrm{lit}}$, where $r \in \{\mathrm{ALA}, \mathrm{ARG}, \mathrm{ASN}, \ldots\}$ denotes the residue type, $f \in \mathcal{S}_{\text{torsion names}}$ denotes the frame and $a$ the atom name. We further pre-compute rigid transformations that transform atom coordinates from each frame to the frame that is higher up in the hierarchy. E.g. $T_{r,(\chi_2 \rightarrow \chi_1)}^{\mathrm{lit}}$ maps atoms in amino-acid type $r$ from the $\chi_2$-frame to the $\chi_1$-frame. As we are only predicting heavy atoms, the extra backbone rigid groups $\omega$ and $\phi$ do not contain atoms, but the corresponding frames contribute to the FAPE loss for alignment to the ground truth (like all other frames).

**Algorithm 24** Compute all atom coordinates

**def** computeAllAtomCoordinates$(T_i, \vec{\alpha}_i^f, F_i^{\text{aatype}})$ :

$\hat{\vec{\alpha}}_i^f = \vec{\alpha}_i^f / \|\vec{\alpha}_i^f\|$

$(\vec{\omega}_i, \vec{\phi}_i, \vec{\psi}_i, \vec{\chi}_{1_i}, \vec{\chi}_{2_i}, \vec{\chi}_{3_i}, \vec{\chi}_{4_i}) = \hat{\vec{\alpha}}_i^f$

*# Make extra backbone frames.*

$r_i = F_i^{\text{aatype}}$

$T_{i1} = T_i \circ T^{\text{lit}}_{r_i,(\omega \to \text{bb})} \circ \text{makeRotX}(\vec{\omega}_i)$

$T_{i2} = T_i \circ T^{\text{lit}}_{r_i,(\phi \to \text{bb})} \circ \text{makeRotX}(\vec{\phi}_i)$

$T_{i3} = T_i \circ T^{\text{lit}}_{r_i,(\psi \to \text{bb})} \circ \text{makeRotX}(\vec{\psi}_i)$

*# Make side chain frames (chain them up along the side chain).*

$T_{i4} = T_i \circ T^{\text{lit}}_{r_i,(\chi_1 \to \text{bb})} \circ \text{makeRotX}(\vec{\chi}_{1_i})$

$T_{i5} = T_{i4} \circ T^{\text{lit}}_{r_i,(\chi_2 \to \chi_1)} \circ \text{makeRotX}(\vec{\chi}_{2_i})$

$T_{i6} = T_{i5} \circ T^{\text{lit}}_{r_i,(\chi_3 \to \chi_2)} \circ \text{makeRotX}(\vec{\chi}_{3_i})$

$T_{i7} = T_{i6} \circ T^{\text{lit}}_{r_i,(\chi_4 \to \chi_3)} \circ \text{makeRotX}(\vec{\chi}_{4_i})$

*# Map atom literature positions to the global frame.*

$\vec{\mathbf{x}}_i^a = \text{concat}_{f,a'} \left( \{ T_i^f \circ \vec{\mathbf{x}}^{\text{lit}}_{r_i,f,a'} \} \right)$

**return** $T_i^f, \vec{\mathbf{x}}_i^a$

**Algorithm 25** Make a transformation that rotates around the x-axis

**def** makeRotX$(\vec{\alpha})$ : $\vec{\alpha} \in R^2$ with $\|\vec{\alpha}\| = 1$

$R = \begin{pmatrix} 1 & 0 & 0 \\ 0 & \alpha_1 & -\alpha_2 \\ 0 & \alpha_2 & \alpha_1 \end{pmatrix}$

$\vec{\mathbf{t}} = \begin{pmatrix} 0 \\ 0 \\ 0 \end{pmatrix}$

$T = (R, \vec{\mathbf{t}})$

**return** $T$

### 1.8.5 Rename symmetric ground truth atoms

The 180°-rotation-symmetry for some of the rigid groups (see Table 2) leads to naming ambiguities for the atoms in this group for all atoms that are not on the rotation axis (see Table 3).

**Table 3** | Ambiguous atom names due to $180°$-rotation-symmetry for some of the rigid groups

| aatype | renaming swaps |
|---|---|
| ASP | $\mathrm{O}^{\delta 1} \leftrightarrow \mathrm{O}^{\delta 2}$ |
| GLU | $\mathrm{O}^{\epsilon 1} \leftrightarrow \mathrm{O}^{\epsilon 2}$ |
| PHE | $\mathrm{C}^{\delta 1} \leftrightarrow \mathrm{C}^{\delta 2}$, $\mathrm{C}^{\epsilon 1} \leftrightarrow \mathrm{C}^{\epsilon 2}$ |
| TYR | $\mathrm{C}^{\delta 1} \leftrightarrow \mathrm{C}^{\delta 2}$, $\mathrm{C}^{\epsilon 1} \leftrightarrow \mathrm{C}^{\epsilon 2}$ |

Algorithm 26 resolves the naming ambiguities in a globally consistent way by renaming the ground truth structure: For each residue, it computes the lDDT of the atoms against all non-ambiguous atoms for both possible namings ("true" and "alt truth") of the ground truth atoms. The set of non-ambiguous atoms $\mathcal{S}_{\text{non-ambiguous atoms}}$ is the set of all (residue-type, atom-type) tuples from Table 2 minus the set of ambiguous atoms from Table 3. Subsequently the algorithm renames the ambiguous ground truth atoms such that they fit best to the predicted structure.

**Algorithm 26** Rename symmetric ground truth atoms

**def** $\mathrm{renameSymmetricGroundTruthAtoms}(\{T_i^f\}, \{\vec{\mathbf{x}}_i^a\}, \{T_i^{\text{true},f}\}, \{T_i^{\text{alt truth},f}\}, \{\vec{\mathbf{x}}_i^{\text{true},a}\}, \{\vec{\mathbf{x}}_i^{\text{alt truth},a}\})$ :

$d_{(i,a),(j,b)} = \left\| \vec{\mathbf{x}}_i^a - \vec{\mathbf{x}}_j^b \right\| \quad \forall (j,b) \in \mathcal{S}_{\text{non-ambiguous atoms}}$

$d^{\text{true}}_{(i,a),(j,b)} = \left\| \vec{\mathbf{x}}_i^{\text{true},a} - \vec{\mathbf{x}}_j^{\text{true},b} \right\| \quad \forall (j,b) \in \mathcal{S}_{\text{non-ambiguous atoms}}$

$d^{\text{alt truth}}_{(i,a),(j,b)} = \left\| \vec{\mathbf{x}}_i^{\text{alt truth},a} - \vec{\mathbf{x}}_j^{\text{true},b} \right\| \quad \forall (j,b) \in \mathcal{S}_{\text{non-ambiguous atoms}}$

**for all** $i \in [1 \ldots N_{\text{res}}]$ **do**

  **if** $\mathrm{sum}_{a,(j,b)} \left( \left| d_{(i,a),(j,b)} - d^{\text{alt truth}}_{(i,a),(j,b)} \right| \right) < \mathrm{sum}_{a,(j,b)} \left( \left| d_{(i,a),(j,b)} - d^{\text{true}}_{(i,a),(j,b)} \right| \right)$ **then**

    $\vec{\mathbf{x}}_i^{\text{true},a} \leftarrow \vec{\mathbf{x}}_i^{\text{alt truth},a}$

    $T_i^{\text{true},f} \leftarrow T_i^{\text{alt truth},f}$

  **end if**

**end for**

**return** $\{T_i^{\text{true},f}\}, \{\vec{\mathbf{x}}_i^{\text{true},a}\}$

### 1.8.6 Amber relaxation

In order to resolve any remaining structural violations and clashes [99], we relax our model predictions by an iterative restrained energy minimization procedure. At each round, we perform minimization of the AMBER99SB [100] force field with additional harmonic restraints that keep the system near its input structure. The restraints are applied independently to heavy atoms, with a spring constant of 10 kcal/mol $\text{Å}^2$. Once the minimizer has converged, we determine which residues still contain violations. We then remove restraints from all atoms within these residues and perform restrained minimization once again, starting from the minimized structure of the previous iteration. This process is repeated until all violations are resolved. In the CASP14 assessment we used a single iteration; targets with unresolved violations were re-run.

Full energy minimization and hydrogen placement was performed using the OpenMM simulation package [101]. The minimization procedure was run with a tolerance of 2.39 kcal/mol and an unbounded maximum number of steps, which are OpenMM's default values.

## 1.9 Loss functions and auxiliary heads

The network is trained end-to-end, with gradients coming from the main Frame Aligned Point Error (FAPE) loss $\mathcal{L}_{\text{FAPE}}$ and a number of auxiliary losses. The total per-example loss can be defined as follows

$$\mathcal{L} = \begin{cases} 0.5\mathcal{L}_{\text{FAPE}} + 0.5\mathcal{L}_{\text{aux}} + 0.3\mathcal{L}_{\text{dist}} + 2.0\mathcal{L}_{\text{msa}} + 0.01\mathcal{L}_{\text{conf}} & \text{training} \\ 0.5\mathcal{L}_{\text{FAPE}} + 0.5\mathcal{L}_{\text{aux}} + 0.3\mathcal{L}_{\text{dist}} + 2.0\mathcal{L}_{\text{msa}} + 0.01\mathcal{L}_{\text{conf}} + 0.01\mathcal{L}_{\text{exp resolved}} + 1.0\mathcal{L}_{\text{viol}} & \text{fine-tuning} \end{cases}, \tag{7}$$

where $\mathcal{L}_{\text{aux}}$ is the auxiliary loss from the Structure Module (averaged FAPE and torsion losses on the intermediate structures, defined in Algorithm 20 line 23), $\mathcal{L}_{\text{dist}}$ is an averaged cross-entropy loss for distogram prediction, $\mathcal{L}_{\text{msa}}$ is an averaged cross-entropy loss for masked MSA prediction, $\mathcal{L}_{\text{conf}}$ is the model confidence loss defined in subsubsection 1.9.6, $\mathcal{L}_{\text{exp resolved}}$ is the "experimentally resolved" loss defined in subsubsection 1.9.10, and $\mathcal{L}_{\text{viol}}$ is the violation loss defined in subsubsection 1.9.11. The last two losses are only used during fine-tuning.

To decrease the relative importance of short sequences, we multiply the final loss of each training example by the square root of the number of residues after cropping. This implies equal weighting for all proteins that are longer than the crop size, and a square-root penalty for the shorter ones.

The purpose of the FAPE, aux, distogram, and MSA losses is to attach an individual loss to each major subcomponent of the model (including both the pair and MSA final embeddings) as a guide during the training of the "purpose" of each unit. The FAPE and aux are direct structural terms for the Structure module. The distogram loss assures that all entries in the pair representation have a clear relationship to the associated $ij$ residue pair and to make sure that the pair representation will be useful for the structure module (ablations show that this is only a minor effect however). The distogram is also a distributional prediction, such that it is a method by which we interpret the model's confidence in interdomain interactions. The MSA loss is intended to force the network to consider inter-sequence or phylogenetic relationships to complete the BERT task, which we intend as a way to encourage the model to consider co-evolution-like relationships without explicitly encoding covariance statistics (this is the intention but we only observe the outcome that it increases model accuracy). The very small confidence loss allows the construction of the pLDDT value without compromising the accuracy of the structures themselves – we had previously fine-tuned this loss after training but it is just as accurate to train from the beginning with a small loss. Finally, the "violation" losses encourage the model to produce a physically plausible structure with correct bond geometry and avoidance of clashes, even in cases where the model is highly unsure of the structure. This avoids rare failures or accuracy loss in the final AMBER relaxation. Using the violation losses early in training causes a small drop in final accuracy since the model overly optimizes for the avoidance of clashes, so we only use this during fine-tuning.

The various loss weights were hand-selected and only lightly-tuned over the course of AlphaFold development (typically a couple of values for the coefficient of each loss was tried when the loss term was introduced and the weights rarely adjusted after that). We did some tuning early in model development on the ratio of FAPE, distogram, and MSA losses, but did not re-tune much as the model developed. It is possible that automated or more extensive tuning of these weights could improve accuracy, but we have generally not observed strong sensitivity to the precise values that would motivate us to do so.

Below we provide details of the individual losses and transformations that are applied to the Evoformer output representations to obtain auxiliary predictions.

### 1.9.1 Side chain and backbone torsion angle loss

The predicted side chain and backbone torsion angles are represented as points on the unit circle, i.e., $\hat{\vec{\alpha}}_i^f \in \mathbb{R}^2$ with $\|\hat{\vec{\alpha}}_i^f\| = 1$. They are compared to the true torsion angles $\vec{\alpha}_i^{\text{true},f}$ with an L2 loss in $\mathbb{R}^2$, which is mathematically equivalent to the cosine of the angle difference (see below).

Some side chains parts are $180°$-rotation-symmetric (see Table 2), such that the predicted torsion angle $\chi$ and $\chi + \pi$ result in the same physical structure (just the internal atom names are exchanged). We allow the

network to produce either of these torsion angles by providing the alternative angle as $\vec{\alpha}_i^{\text{alt truth},f} = \vec{\alpha}_i^{\text{true},f} + \pi$. For all non-symmetric configurations we set $\vec{\alpha}_i^{\text{alt truth},f} = \vec{\alpha}_i^{\text{true},f}$.

We also introduce a small auxiliary loss $\mathcal{L}_{\text{anglenorm}}$ that keeps the predicted points close to the unit circle. There are two reasons for this: One is to avoid vectors too close to the origin, which can lead to numerically unstable gradients. The other is that while the norm of the vector does not influence the output it does influence the learning dynamics of the network. When looking at how the gradients transform in the backward pass of the normalization the gradients will get rescaled by the norm of the unnormalized vector.

Given that the model is highly nonlinear the length of these vectors can change strongly during training, leading to undesirable learning dynamics. The weighting factor was selected on an ad hoc basis, testing a few values and picking the lowest one that keeps the norm of the vectors stable. We have not observed any strong dependence on the precise value in terms of model performance.

**Algorithm 27** Side chain and backbone torsion angle loss

**def** $\text{torsionAngleLoss}(\{\vec{\alpha}_i^f\}, \{\vec{\alpha}_i^{\text{true},f}\}, \{\vec{\alpha}_i^{\text{alt truth},f}\})$ :

$\ell_i^f = \|\vec{\alpha}_i^f\|$

$\hat{\vec{\alpha}}_i^f = \vec{\alpha}_i^f / \ell_i^f$

$\mathcal{L}_{\text{torsion}} = \text{mean}_{i,f}(\text{minimum}(\|\hat{\vec{\alpha}}_i^f - \vec{\alpha}_i^{\text{true},f}\|^2,\ \|\hat{\vec{\alpha}}_i^f - \vec{\alpha}_i^{\text{alt truth},f}\|^2))$

$\mathcal{L}_{\text{anglenorm}} = \text{mean}_{i,f}(\{|\ell_i^f - 1|\})$

**return** $\mathcal{L}_{\text{torsion}} + 0.02\mathcal{L}_{\text{anglenorm}}$

The comparison of two angles ($\alpha$ and $\beta$) represented as points on the unit circle by an L2 norm is mathematically equivalent to the cosine of the angle difference:

$$\begin{aligned} \cos(\alpha - \beta) &= \cos\alpha \cdot \cos\beta + \sin\alpha \cdot \sin\beta \\ &= \begin{pmatrix} \cos\alpha \\ \sin\alpha \end{pmatrix}^\top \begin{pmatrix} \cos\beta \\ \sin\beta \end{pmatrix} \end{aligned} \tag{8}$$

$$\begin{aligned} \left\| \begin{pmatrix} \cos\alpha \\ \sin\alpha \end{pmatrix} - \begin{pmatrix} \cos\beta \\ \sin\beta \end{pmatrix} \right\|^2 &= \left\| \begin{pmatrix} \cos\alpha \\ \sin\alpha \end{pmatrix} \right\|^2 + \left\| \begin{pmatrix} \cos\beta \\ \sin\beta \end{pmatrix} \right\|^2 - 2 \begin{pmatrix} \cos\alpha \\ \sin\alpha \end{pmatrix}^\top \begin{pmatrix} \cos\beta \\ \sin\beta \end{pmatrix} \\ &= 2 - 2 \begin{pmatrix} \cos\alpha \\ \sin\alpha \end{pmatrix}^\top \begin{pmatrix} \cos\beta \\ \sin\beta \end{pmatrix} \\ &= 2 - 2\cos(\alpha - \beta), \end{aligned} \tag{9}$$

where the first identity is just the normal cosine difference formula.

### 1.9.2 Frame aligned point error (FAPE)

The Frame Aligned Point Error (FAPE) (see Algorithm 28, and **Fig. 3f** with its corresponding section in the main article) scores a set of predicted atom coordinates $\{\vec{\mathbf{x}}_j\}$ under a set of predicted local frames $\{T_i\}$ against the corresponding ground truth atom coordinates $\{\vec{\mathbf{x}}_j^{\text{true}}\}$ and ground truth local frames $\{T_i^{\text{true}}\}$. The final FAPE loss (Algorithm 20 line 28) scores all atoms in all backbone and side chain frames. Additionally a cheaper version (scoring only all $C\alpha$ atoms in all backbone frames) is used as an auxiliary loss in every layer of the Structure Module (Algorithm 20 line 17).

In order to formulate the loss we compute the atom position $\vec{\mathbf{x}}_j$ relative to frame $T_i$ (Algorithm 28 line 1) and the location of the corresponding true atom position $\vec{\mathbf{x}}_j^{\text{true}}$ relative to the true frame $T_i^{\text{true}}$ (Algorithm 28 line 2). The deviation is computed as a robust L2 norm (Algorithm 28 line 3) ($\epsilon$ is a small constant added to ensure that gradients are numerically well behaved for small differences. The exact value of this constant does not matter, as long as it is small enough. We used values of $10^{-4}$ and $10^{-12}$ in our experiments.). The

resulting $N_{\text{frames}} \times N_{\text{atoms}}$ deviations are penalized with a clamped L1-loss (Algorithm 28 line 4) with a length scale $Z = 10$ Å to make the loss unitless.

In this section, we represent the point positions and the hyperparameters in Å, although the loss is invariant to the choice of units.

We now discuss the behaviour of the loss under global rigid transformations of both the true and the predicted structure. Firstly we should note that $\vec{\mathbf{x}}_{ij}$ is invariant under rigid motions (not including reflection); as such if the predicted structure differs from the ground truth by an arbitrary rotation and translation the loss will stay constant. However, the loss is not invariant under reflections due to the way the local frames are constructed, as the z-axis of the local frames transforms as a pseudo-vector. Furthermore, we can see that the error is invariant when applying the same rigid motion to both target frames and predicted frames. This implies that the way frames are constructed from the structure is not constrained to the precise choice we made, but can differ as long as they are constructed in a consistent manner between predicted and target structure.

**Algorithm 28** Compute the Frame aligned point error

**def** $\text{computeFAPE}(\{T_i\}, \{\vec{\mathbf{x}}_j\}, \{T_i^{\text{true}}\}, \{\vec{\mathbf{x}}_j^{\text{true}}\}, Z = 10\text{Å}, d_{\text{clamp}} = 10\text{Å}, \epsilon = 10^{-4}\text{Å}^2):$

$T_i, T_i^{\text{true}} \in (\mathbb{R}^{3\times3}, \mathbb{R}^3)$
$\vec{\mathbf{x}}_j, \vec{\mathbf{x}}_j^{\text{true}} \in \mathbb{R}^3,$
$i \in \{1, ..., N_{\text{frames}}\}, j \in \{1, ..., N_{\text{atoms}}\}$

1: $\vec{\mathbf{x}}_{ij} = T_i^{-1} \circ \vec{\mathbf{x}}_j$ $\qquad \vec{\mathbf{x}}_{ij} \in \mathbb{R}^3$

2: $\vec{\mathbf{x}}_{ij}^{\text{true}} = T_i^{\text{true}-1} \circ \vec{\mathbf{x}}_j^{\text{true}}$ $\qquad \vec{\mathbf{x}}_{ij}^{\text{true}} \in \mathbb{R}^3$

3: $d_{ij} = \sqrt{\|\vec{\mathbf{x}}_{ij} - \vec{\mathbf{x}}_{ij}^{\text{true}}\|^2 + \epsilon}$ $\qquad d_{ij} \in \mathbb{R}$

4: $\mathcal{L}_{\text{FAPE}} = \frac{1}{Z} \,\text{mean}_{i,j}(\text{minimum}(d_{\text{clamp}}, d_{ij}))$

5: **return** $\mathcal{L}_{\text{FAPE}}$

### 1.9.3 Chiral properties of AlphaFold and its loss

In this section we will look in detail at the transformation properties of the various components under global reflections

$$\vec{\mathbf{x}}_i \overset{\text{reflection}}{\mapsto} -\vec{\mathbf{x}}_i \tag{10}$$

Under this global reflection, the coordinates of the frames also change in a non-trivial way (use Algorithm 21 with negated positions). Simple algebra shows that

$$T_i = (R_i, \vec{\mathbf{t}}_i) \overset{\text{reflection}}{\mapsto} \left( R_i \begin{pmatrix} -1 & 0 & 0 \\ 0 & -1 & 0 \\ 0 & 0 & 1 \end{pmatrix}, -\vec{\mathbf{t}}_i \right), \tag{11}$$

where the non-trivial transformation of the rotation matrix $R_i$ arises from the cross-product in Algorithm 21. The non-trivial transformation of the rotation matrix also means that the local points $T_i^{-1} \circ \vec{\mathbf{x}}_j$ are *not* invariant under reflection,

$$T_i^{-1} \circ \vec{\mathbf{x}}_j = R_i^{-1}(\vec{\mathbf{x}}_j - \vec{\mathbf{t}}_i) \tag{12}$$

$$\overset{\text{reflection}}{\mapsto} \begin{pmatrix} -1 & 0 & 0 \\ 0 & -1 & 0 \\ 0 & 0 & 1 \end{pmatrix} R_i^{-1}(-\vec{\mathbf{x}}_j + \vec{\mathbf{t}}_i) \tag{13}$$

$$= \begin{pmatrix} 1 & 0 & 0 \\ 0 & 1 & 0 \\ 0 & 0 & -1 \end{pmatrix} R_i^{-1}(\vec{\mathbf{x}}_j - \vec{\mathbf{t}}_i) \tag{14}$$

$$= \begin{pmatrix} 1 & 0 & 0 \\ 0 & 1 & 0 \\ 0 & 0 & -1 \end{pmatrix} \left( T_i^{-1} \circ \vec{\mathbf{x}}_j \right). \tag{15}$$

The effect of a global reflection is to reflect only the z-component of the local coordinates $T_i^{-1} \circ \vec{\mathbf{x}}_j$.

In the following we denote a set of frames and points by large Roman letters, for example $X = \left(\{\vec{\mathbf{x}}_i\}, \{T_j\}\right)$.

In particular, this means that both FAPE and IPA can distinguish global reflections of the protein assuming that the rigid frames are related to the underlying points by Algorithm 21, e.g.

$$\mathrm{FAPE}(X, X_{\text{reflection}}) = \sum_{ij} \left\| T_i^{-1} \circ \vec{\mathbf{x}}_j - \left(\begin{smallmatrix} 1 & 0 & 0 \\ 0 & 1 & 0 \\ 0 & 0 & -1 \end{smallmatrix}\right) \left(T_i^{-1} \circ \vec{\mathbf{x}}_j\right) \right\| \tag{16}$$

$$= 2 \sum_{ij} \left| (T_i^{-1} \circ \vec{\mathbf{x}}_j)_z \right|, \tag{17}$$

which is a large positive value outside of degenerate cases. For a more general proof that FAPE can distinguish chirality regardless of how the frames are constructed, see the next section.

There are other sources of chirality in AlphaFold. The atom positions are computed from a combination of the backbone frames and the predicted $\chi$ angles, and this procedure will always generate a left-handed molecule since it uses ideal values outside of the $\chi$ angles (i.e. the CB will always be in the left-handed location). AlphaFold can produce almost exactly the chiral pair for the backbone atoms, but it cannot build the chiral pair of the side chains. The model also has a small loss on the $\chi$-angle values and those values are not invariant under reflection.

In order to test the importance of the chirality of FAPE we trained a model using the dRMSD loss [102] instead of FAPE, we show the results on the CASP14 set in Figure 9. Here we can see that the lDDT-C$\alpha$ performance is still good, where we note that lDDT-C$\alpha$ is a metric that cannot distinguish molecules of the opposite chirality.

However GDT shows a bimodal distribution if trained with a dRMSD loss, where one of the two modes is only somewhat worse than baseline AlphaFold and the other mode has very low accuracy (Suppl. Fig. 9). This suggests that the second mode composes molecules of reverse chirality. To test this we compute $\overline{GDT}$, which is the GDT of the mirror reflection of the structure used to compute GDT. These mirror structures also show bimodality of the GDT values. Finally taking the maximum over the GDT of the structure and its mirror produces consistently high GDT. This confirms that AlphaFold trained with a dRMSD loss frequently produces mirror structures, and that FAPE is the main component that ensures the correct chirality of predicted structures.

### 1.9.4 Configurations with FAPE(X,Y) = 0

To understand the points at which zero FAPE loss is achieved, we will introduce an RMSD-like auxiliary measure that operates only on points and not frames

$$S(\{\vec{\mathbf{x}}_i\}, \{\vec{\mathbf{y}}_i\}) = \min_{T^{\text{align}} \in \mathrm{SE}(3)} \frac{1}{N_{\text{point}}} \sum_j \left\| \vec{\mathbf{x}}_j - T^{\text{align}} \circ \vec{\mathbf{y}}_j \right\| \tag{18}$$

where $T$ is a proper rigid transformation. Then we can show a lower bound of FAPE irrespective of the values of the frames,

$$\mathrm{FAPE}(X, \tilde{X}) = \frac{1}{N_{\text{frames}} N_{\text{point}}} \sum_{ij} \left\| T_i^{-1} \circ \vec{\mathbf{x}}_j - \tilde{T}_i^{-1} \circ \vec{\tilde{\mathbf{x}}}_j \right\| \tag{19}$$

$$= \frac{1}{N_{\text{frames}}} \sum_i \left( \frac{1}{N_{\text{point}}} \sum_j \left\| \vec{\mathbf{x}}_j - (T_i \circ \tilde{T}_i^{-1}) \circ \vec{\tilde{\mathbf{x}}}_j \right\| \right) \tag{20}$$

$$\geq \frac{1}{N_{\text{frames}}} \sum_i S(\{\vec{\mathbf{x}}_j\}, \{\vec{\tilde{\mathbf{x}}}_j\}) \tag{21}$$

$$= S(\{\vec{\mathbf{x}}_j\}, \{\vec{\tilde{\mathbf{x}}}_j\}), \tag{22}$$

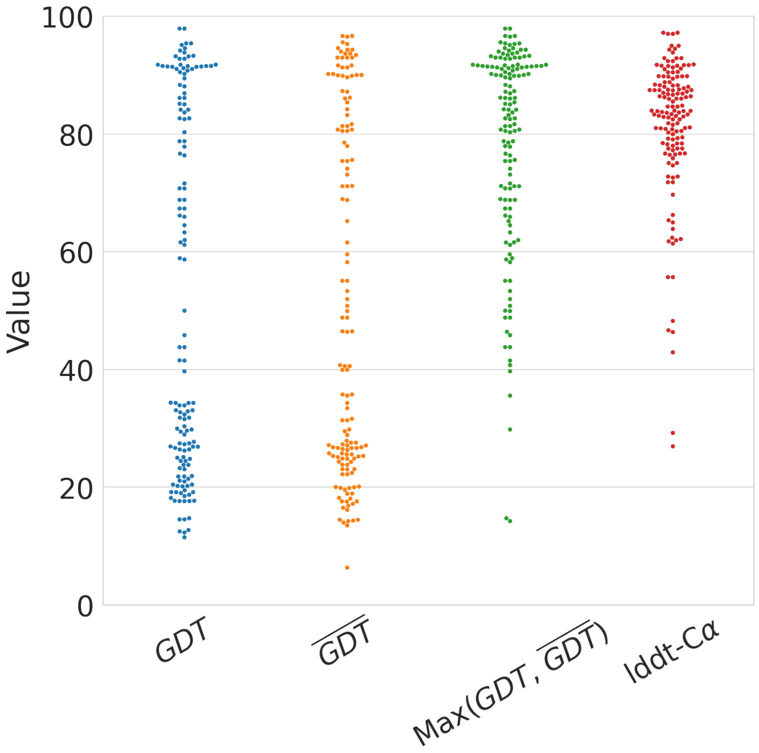


**Supplementary Figure 9** | Accuracy distribution of a model trained with dRMSD instead of the FAPE loss ($N = 87$ protein domains). Here $\overline{\text{GDT}}$ denotes the GDT score obtained when comparing to the chirally reflected target structure.

since the $S$ function minimizes the quantity in parentheses over all proper rigid transformations and $T_i \tilde{T}_i^{-1}$ is a proper rigid transformation. This inequality simply shows that the point errors averaged over all local frames are no less than the point error for the best single global alignment under the same distance function.

The value $S(\{\vec{\mathbf{x}}_i\}, \{\tilde{\vec{\mathbf{x}}}_i\})$ is zero iff $\text{RMSD}(\{\vec{\mathbf{x}}_i\}, \{\tilde{\vec{\mathbf{x}}}_i\}) = 0$, which shows that $\text{FAPE}(X, \tilde{X}) = 0$ is only possible if the points have $\text{RMSD}(\{\vec{\mathbf{x}}_i\}, \{\vec{\mathbf{y}}_i\}) = 0$. We can characterize all pairs such that $\text{FAPE}(X, \tilde{X}) = 0$ as those where $\text{RMSD}(\{\vec{\mathbf{x}}_i\}, \{\tilde{\vec{\mathbf{x}}}_i\}) = 0$ and $T_i^{-1} \circ \tilde{T}_i$ is one of rigid motions that achieves this zero RMSD (which is unique unless the point sets are degenerate). In particular, the FAPE loss always has non-zero values chiral pairs if the point sets are non-degenerate, regardless of the how the frames are constructed.

### 1.9.5 Metric properties of FAPE

In this section, we show that idealized FAPE (i.e. using $\epsilon = 0$) has all the properties of a pseudometric, which is a generalized distance function used in topology. To reduce clutter, the proofs are shown without a cutoff but hold equally well when $\|\cdot\|$ is treated as a clipped vector norm. The reader uninterested in the mathematical details may skip this section.

It is simple to show from the defining equation that

$$\text{FAPE}(X, Y) \geq 0 \tag{23}$$

$$\text{FAPE}(X, X) = 0 \tag{24}$$

$$\text{FAPE}(X, Y) = \text{FAPE}(Y, X), \tag{25}$$

so the only remaining pseudometric property to prove is the triangle inequality, $\text{FAPE}(X, Z) \leq \text{FAPE}(X, Y) + \text{FAPE}(Y, Z)$. The triangle inequality is proven below, using tildes and overbars to designate the different con-

figurations.

$$\begin{aligned}
\mathrm{FAPE}(X, \tilde{X}) &= \sum_{i,j} \left\| T_i^{-1} \circ \vec{\mathbf{x}}_j - \tilde{T}_i^{-1} \circ \vec{\tilde{\mathbf{x}}}_j \right\| && (26) \\
&= \sum_{i,j} \left\| T_i^{-1} \circ \vec{\mathbf{x}}_j - \bar{T}_i^{-1} \circ \vec{\bar{\mathbf{x}}}_j + \bar{T}_i^{-1} \circ \vec{\bar{\mathbf{x}}}_j - \tilde{T}_i^{-1} \circ \vec{\tilde{\mathbf{x}}}_j \right\| && (27) \\
&\leq \sum_{i,j} \left( \left\| T_i^{-1} \circ \vec{\mathbf{x}}_j - \bar{T}_i^{-1} \circ \vec{\bar{\mathbf{x}}}_j \right\| + \left\| \bar{T}_i^{-1} \circ \vec{\bar{\mathbf{x}}}_j - \tilde{T}_i^{-1} \circ \vec{\tilde{\mathbf{x}}}_j \right\| \right) && (28) \\
&= \sum_{i,j} \left\| T_i^{-1} \circ \vec{\mathbf{x}}_j - \bar{T}_i^{-1} \circ \vec{\bar{\mathbf{x}}}_j \right\| + \sum_{i,j} \left\| \bar{T}_i^{-1} \circ \vec{\bar{\mathbf{x}}}_j - \tilde{T}_i^{-1} \circ \vec{\tilde{\mathbf{x}}}_j \right\| && (29) \\
&= \mathrm{FAPE}(X, \bar{X}) + \mathrm{FAPE}(\bar{X}, \tilde{X}). && (30)
\end{aligned}$$

### 1.9.6 Model confidence prediction (pLDDT)

Our model provides intrinsic model accuracy estimates by predicting the per-residue lDDT-C$\alpha$ [99] scores. We call this confidence measure pLDDT. Algorithm 29 computes it by taking the final single representation from the structure module (Algorithm 20 line 30), and projecting it into 50 bins (each bin covering a 2 lDDT-C$\alpha$ range).

For training, we score the final predicted structure with per-residue lDDT-C$\alpha$ against the ground truth structure. This score is discretized into 50 bins and used as the target value for the cross-entropy loss $\mathcal{L}_{\mathrm{conf}}$ averaged over the residues. Additionally, we only train on the examples with resolution between 0.1 Å and 3.0 Å, and ignoring any NMR structure, to ensure high-quality ground truth data.

For evaluation, we compute the expected value of the per-residue pLDDT distribution (Algorithm 29 line 5). The *chain* pLDDT is obtained as an average of the per-residue pLDDT scores within the chain.

**Algorithm 29** Predict model confidence pLDDT

**def** predictPerResidueLDDT_Ca($\{\mathbf{s}_i\}, \mathbf{v}_{\mathrm{bins}} = [1, 3, 5, \ldots, 99]^\top, \{r_i^{\mathrm{true\ LDDT}}\}, c = 128$) :

1: $\mathbf{a}_i = \mathrm{relu}(\mathrm{Linear}(\mathrm{relu}(\mathrm{Linear}(\mathrm{LayerNorm}(\mathbf{s}_i)))))$ — $\mathbf{a}_i$, and intermediate activations $\in \mathbb{R}^c$
2: $\mathbf{p}_i^{\mathrm{pLDDT}} = \mathrm{softmax}(\mathrm{Linear}(\mathbf{a}_i))$ — $\mathbf{p}_i^{\mathrm{pLDDT}} \in \mathbb{R}^{|\mathbf{v}_{\mathrm{bins}}|}$
3: $\mathbf{p}_i^{\mathrm{true\ LDDT}} = \mathrm{one\_hot}(r_i^{\mathrm{true\ LDDT}}, \mathbf{v}_{\mathrm{bins}})$
4: $\mathcal{L}_{\mathrm{conf}} = \mathrm{mean}_i(\mathbf{p}_i^{\mathrm{true\ LDDT}\top} \log \mathbf{p}_i^{\mathrm{pLDDT}})$
5: $r_i^{\mathrm{pLDDT}} = \mathbf{p}_i^{\mathrm{pLDDT}\top} \mathbf{v}_{\mathrm{bins}}$ — $r_i^{\mathrm{pLDDT}} \in \mathbb{R}$
6: **return** $\{r_i^{\mathrm{pLDDT}}\}, \mathcal{L}_{\mathrm{conf}}$

### 1.9.7 TM-score prediction

The pLDDT head above predicts the value of lDDT-C$\alpha$, which is a local error metric that operates pairwise but by design is not sensitive to what fraction of the residues can be aligned using a single global rotation and translation. This can be disadvantageous for assessing whether the model is confident in its overall domain packing for large chains. In this section we develop a predictor of the global superposition metric TM-score [103].

We denote the ground truth structure's C$\alpha$ atoms $X^{\mathrm{true}} = \{\vec{\mathbf{x}}_j^{\mathrm{true}}\}$, its backbone frames $\{T_i^{\mathrm{true}}\}$, the predicted structure's C$\alpha$ atoms via $X = \{\vec{\mathbf{x}}_j\}$, and the corresponding backbone frames $\{T_i\}$. Let the number of residues be $N_{\mathrm{res}}$, and assume all residues are resolved in the ground truth. Denoting $T^{\mathrm{align}} = (R^{\mathrm{align}}, \vec{\mathbf{t}}^{\mathrm{align}})$ an arbitrary

rigid transformation, the TM-score is defined as

$$\mathrm{TM}(X, X^{\mathrm{true}}) = \max_{T^{\mathrm{align}} \in \mathrm{SE}(3)} \frac{1}{N_{\mathrm{res}}} \sum_{j=1}^{N_{\mathrm{res}}} f\left(\|\vec{\mathbf{x}}_j - T^{\mathrm{align}} \circ \vec{\mathbf{x}}_j^{\mathrm{true}}\|\right), \tag{31}$$

$$f(d) = \frac{1}{1 + \left(\frac{d}{d_0(N_{\mathrm{res}})}\right)^2}, \tag{32}$$

$$d_0(N_{\mathrm{res}}) = 1.24 \sqrt[3]{\mathrm{maximum}(N_{\mathrm{res}}, 19) - 15} - 1.8 \tag{33}$$

In the last equation, $N_{\mathrm{res}}$ is clipped to avoid negative or undefined values for very short proteins. In practice, global optimisation over $T^{\mathrm{align}}$ is infeasible, so computational methods rely on approximations, e.g. TM-align [104] uses an iterative procedure of choosing a best-fit alignment on a subset of residues and then re-computing this set of residues.

We now develop an approximation to the TM-score that relies on a pairwise accuracy measure which can be predicted from a pair representation. Crucially, this approximation allows prediction of the TM-score for an arbitrary subset of residues (such as a domain) without rerunning the structure prediction.

$$\mathrm{TM}(X, X^{\mathrm{true}}) = \max_{T^{\mathrm{align}} \in \mathrm{SE}(3)} \frac{1}{N_{\mathrm{res}}} \sum_{j} f\left(\|\vec{\mathbf{x}}_j - T^{\mathrm{align}} \circ \vec{\mathbf{x}}_j^{\mathrm{true}}\|\right) \tag{34}$$

$$\geq \max_{i \in [1,\ldots,N_{\mathrm{res}}]} \frac{1}{N_{\mathrm{res}}} \sum_{j} f\left(\|\vec{\mathbf{x}}_j - (T_i T_i^{\mathrm{true}\,-1}) \circ \vec{\mathbf{x}}_j^{\mathrm{true}}\|\right) \tag{35}$$

$$= \max_{i} \frac{1}{N_{\mathrm{res}}} \sum_{j} f\left(\|T_i^{-1} \circ \vec{\mathbf{x}}_j - T_i^{\mathrm{true}\,-1} \circ \vec{\mathbf{x}}_j^{\mathrm{true}}\|\right). \tag{36}$$

Above, we replace the continuous maximum over all possible alignments with a discrete maximum set of $N_{\mathrm{res}}$ single-residue alignments ($T_i T_i^{\mathrm{true}\,-1}$). This clearly lower-bounds the original maximum, with the bound becoming tight when the global superposition exactly aligns the backbone of any residue. Then, we symmetrize the vector difference by applying the rigid transformation $T_i$. Note that the expression in Equation 36 is closely related to FAPE, except that the $f$ function is somewhat different and FAPE has a mean instead of maximum (i.e. FAPE considers *all* 1-residue alignments instead of only the best one).

Next, we assume that the correct structure $X^{\mathrm{true}}$ is unknown and that we have a distribution of probable structures for which we seek the expected value of the TM-score for our prediction $X$:

$$\mathbb{E}\left[\mathrm{TM}(X, X^{\mathrm{true}})\right] \geq \mathbb{E}\left[\max_{i} \frac{1}{N_{\mathrm{res}}} \sum_{j} f\left(\|T_i^{-1} \circ \vec{\mathbf{x}}_j - T_i^{\mathrm{true}\,-1} \circ \vec{\mathbf{x}}_j^{\mathrm{true}}\|\right)\right] \tag{37}$$

$$\geq \max_{i} \frac{1}{N_{\mathrm{res}}} \sum_{j} \mathbb{E}\left[f\left(\|T_i^{-1} \circ \vec{\mathbf{x}}_j - T_i^{\mathrm{true}\,-1} \circ \vec{\mathbf{x}}_j^{\mathrm{true}}\|\right)\right]. \tag{38}$$

In the final line, we replace the expectation-of-maximum with a maximum-of-expectation (a lower bound due to Jensen's inequality), and use the linearity of expectation.

The pairwise matrix $e_{ij} = \|T_i^{-1} \circ \vec{\mathbf{x}}_j - T_i^{\mathrm{true}\,-1} \circ \vec{\mathbf{x}}_j^{\mathrm{true}}\|$ is a non-symmetric matrix that captures the error in the position of the C$\alpha$ atom of residue $j$ when the predicted and true structures are aligned using the backbone frame of residue $i$. The probability distribution of its elements can be readily predicted by a neural network. To do that, we discretize the distribution of $e_{ij}$ into 64 bins, covering the range from 0 to 31.5 Å with 0.5 Å bin width. During training, the final bin also captures any larger errors. We compute $e_{\mathrm{ij}}$ as a linear projection of the pair representation $\mathbf{z}_{ij}$, followed by a softmax. We fine-tune the CASP models to additionally predict $e_{ij}$ using the average categorical cross-entropy loss, with weight 0.1 (weights 0.01 and 1.0 were also tried but those weights lowered either pTM accuracy or structure prediction accuracy, respectively). Just as for

the pLDDT prediction, we only train this prediction module on non-NMR examples with resolution between $0.1$ Å and $3.0$ Å. The fine-tuning took roughly 16 hours and processed $3 \cdot 10^5$ samples.

Using $e_{ij}$, we approximate the TM-score as

$$\text{pTM} = \max_i \frac{1}{N_{\text{res}}} \sum_j \mathbb{E}\left[f(e_{ij})\right], \tag{39}$$

where the expectation is taken over the probability distribution defined by $e_{ij}$. We find that pTM is a pessimistic predictor of the true TM-score, which is as expected, given that both approximations used in the derivation of pTM are lower bounds.

Note that given a full-chain prediction of $e_{ij}$, we can obtain a TM-score prediction for any subset of residues $\mathcal{D}$ (such as a particular domain) simply by restricting the range of residues considered:

$$\text{pTM}(\mathcal{D}) = \max_{i \in \mathcal{D}} \frac{1}{|\mathcal{D}|} \sum_{j \in \mathcal{D}} \mathbb{E} \frac{1}{1 + \left(\frac{e_{ij}}{d_0(|\mathcal{D}|)}\right)^2}, \tag{40}$$

where $|\mathcal{D}|$ is the number of residues in the set $\mathcal{D}$. By small modifications, a similar expression could be derived to estimate GDT, FAPE, or RMSD from the $e_{ij}$ matrix, though we do not pursue this further. Additionally, the 2-D image of the expected values of $f(e_{ij})$ serves as a good visualization of confident domain packings within the structure.

### 1.9.8 Distogram prediction

We linearly project the symmetrized pair representations $(\mathbf{z}_{ij} + \mathbf{z}_{ji})$ into $64$ distance bins and obtain the bin probabilities $p_{ij}^b$ with a softmax. The bins cover the range from 2 Å to 22 Å; and they have equal width apart from the last bin, which also includes any more distant residue pairs. For prediction targets $y_{ij}^b$ we use one-hot encoded binned residue distances, which are computed from the ground-truth beta carbon positions for all amino acids except glycine where use alpha carbon instead. We use the cross-entropy loss averaged over all residue pairs:

$$\mathcal{L}_{\text{dist}} = -\frac{1}{N_{\text{res}}^2} \sum_{i,j} \sum_{b=1}^{64} y_{ij}^b \log p_{ij}^b \,. \tag{41}$$

### 1.9.9 Masked MSA prediction

Similarly to the common masked language modelling objectives [105], we use the final MSA representations to reconstruct MSA values that have been previously masked out (see subsubsection 1.2.7). We consider 23 classes, which include 20 common amino acid types, an unknown type, a gap token, and a mask token. MSA representations $\{\mathbf{m}_{si}\}$ are linearly projected into the output classes, passed through a softmax, and scored with the cross-entropy loss:

$$\mathcal{L}_{\text{msa}} = -\frac{1}{N_{\text{mask}}} \sum_{s,i \in \text{mask}} \sum_{c=1}^{23} y_{si}^c \log p_{si}^c \,, \tag{42}$$

where $p_{si}^c$ are predicted class probabilities, $y_{si}^c$ are one-hot encoded ground-truth values, and averaging happens over the masked positions.

### 1.9.10 "Experimentally resolved" prediction

The model contains a head that predicts if an atom is experimentally resolved in a high-resolution structure. The input for this head is the single representation $\{\mathbf{s}_i\}$ produced by the Evoformer stack (subsection 1.6). The single representation is projected with a Linear layer and a sigmoid to atom-wise probabilities $\{p_i^{\text{exp resolved},a}\}$

with $i \in [1, \dots, N_{\text{res}}]$ and $a \in \mathcal{S}_{\text{atom names}}$. We train this head during fine-tuning (see subsubsection 1.12.1) on high-resolution X-ray crystals and cryo-EM structures (resolution better than 3Å) with standard cross-entropy,

$$\mathcal{L}_{\text{exp resolved}} = \text{mean}_{(i,a)} \left( -y_i^a \log p_i^{\text{exp resolved},a} - (1 - y_i^a) \log(1 - p_i^{\text{exp resolved},a}) \right) , \tag{43}$$

where $y_i^a \in \{0, 1\}$ denotes the ground truth, i.e. if atom $a$ in residue $i$ was resolved in the experiment.

### 1.9.11 Structural violations

The construction of the atom coordinates from independent backbone frames and torsion-angles (**Fig. 3e**), subsubsection 1.8.4) produces idealized bond lengths and bond angles for most of the atom bonds, but the geometry for inter-residue bonds (peptide bonds) and the avoidance of atom clashes need to be learned. We introduce extra losses that penalize these structural violations to ensure these constraints everywhere, i.e. also in regions where no ground truth atom coordinates are available. We constructed the losses in a way that loss-free structures will pass the stereochemical quality checks in the lDDT metric [99].

The losses use a flat-bottom L1 loss that penalizes violations above a certain tolerance threshold $\tau$. The bond length violation loss is computed as

$$\mathcal{L}_{\text{bondlength}} = \frac{1}{N_{\text{bonds}}} \sum_{i=1}^{N_{\text{bonds}}} \max \left( |\ell_{\text{pred}}^i - \ell_{\text{lit}}^i| - \tau,\ 0 \right) , \tag{44}$$

where $\ell_{\text{pred}}^i$ is a bond length in the predicted structure and $\ell_{\text{lit}}^i$ is the literature value [98] for this bond length. $N_{\text{bonds}}$ is the number of all bonds in this structure. We set the tolerance $\tau$ to $12\sigma_{\text{lit}}$, where $\sigma_{\text{lit}}$ is the literature standard deviation of this bond length. We chose the factor 12 to ensure that the produced bond lengths pass the stereochemical quality checks lDDT metric [99], which also uses by default a tolerance factor of 12.

The bond angle violation loss uses the cosine of the angle computed from the dot-product of the unit vectors of the bonds, $\cos \alpha = \hat{\vec{\mathbf{v}}}_1^\top \hat{\vec{\mathbf{v}}}_2$ and also applies a flat-bottom L1 loss on the deviations:

$$\mathcal{L}_{\text{bondangle}} = \frac{1}{N_{\text{angles}}} \sum_{i=1}^{N_{\text{angles}}} \max \left( |\cos \alpha_{\text{pred}}^i - \cos \alpha_{\text{lit}}^i| - \tau,\ 0 \right) \tag{45}$$

where $\alpha_{\text{pred}}^i$ is a bond angle in the predicted structure and $\alpha_{\text{lit}}^i$ is the literature value for this bond angle. $N_{\text{angles}}$ is the number of all bond angles in this structure. The tolerance $\tau$ is computed such that the flat bottom extends from -12 to 12 times the literature standard deviation of this bond angle.

The clash loss uses a one-sided flat-bottom-potential, that penalizes too short distances only,

$$\mathcal{L}_{\text{clash}} = \sum_{i=1}^{N_{\text{nbpairs}}} \max \left( d_{\text{lit}}^i - \tau - d_{\text{pred}}^i,\ 0 \right) , \tag{46}$$

where $d_{\text{pred}}^i$ is the distance of two non-bonded atoms in the predicted structure and $d_{\text{lit}}^i$ is the "clashing distance" of these two atoms according to their literature Van der Waals radii. $N_{\text{nbpair}}$ is the number of all non-bonded atom pairs in this structure. The tolerance $\tau$ is set to $1.5$ Å.

All these losses together build the violation loss

$$\mathcal{L}_{\text{viol}} = \mathcal{L}_{\text{bondlength}} + \mathcal{L}_{\text{bondangle}} + \mathcal{L}_{\text{clash}} . \tag{47}$$

We apply this violation loss only during the fine-tuning training phase. Switching it on in the early training leads to strong instabilities in the training dynamics.

### 1.10 Recycling iterations

We find it helpful to execute the network multiple times, each time embedding the previous outputs as additional inputs. We call this technique "recycling", and we find it relatively important in the ablation studies (subsection 1.13). In this section we first explain how recycling operates in general, both at training and the inference time. Then, we describe the specific features being recycled in the AlphaFold model. Algorithm 2 shows the full inference of AlphaFold with recycling and ensembling (MSA resampling and ensembling is further explained in subsubsection 1.11.2).

**Algorithm 30** Generic recycling inference procedure

**def** $\text{RecyclingInference}(\{\text{inputs}_c\}, N_\text{cycle})$ :

outputs $= \mathbf{0}$

*# Recycling iterations*

**for all** $c \in [1, \ldots, N_\text{cycle}]$ **do** *# shared weights*

  outputs $\leftarrow$ Model(inputs$_c$, outputs)

**end for**

**return** outputs

**Algorithm 31** Generic recycling training procedure

**def** $\text{RecyclingTraining}(\{\text{inputs}_c\}, N_\text{cycle})$ :

$N' = \text{uniform}(1, N_\text{cycle})$ *# shared value across the batch*

outputs $= \mathbf{0}$

*# Recycling iterations*

**for all** $c \in [1, \ldots, N']$ **do** *# shared weights*

  outputs $\leftarrow$ stopgrad(outputs) *# no gradients between iterations*

  outputs $\leftarrow$ Model(inputs$_c$, outputs)

**end for**

**return** loss(outputs) *# only the final iteration's outputs are used*

Recycling allows to make the network deeper and to process multiple versions of the input features (e.g. incorporate the MSA resampling) without significantly increasing the training time or the number of parameters. At the inference time, recycling yields a recurrent network with shared weights, where each iteration $c$ takes in the input features inputs$_c$ and the previous iteration's outputs, and produces the new refined outputs, see Algorithm 30. The initial "outputs" are constant zeros, since we find that networks can easily accommodate this special case. We will denote the number of iterations of this network via $N_\text{cycle}$. We use $N_\text{cycle} = 4$ in AlphaFold.

While it is possible to train recycling by simply unrolling the $N_\text{cycle}$ iterations, the computational and memory cost of that may be prohibitive. Instead, we introduce an approximate training scheme, which empirically works well and significantly reduces the extra cost of recycling during training, Algorithm 31. We first define an objective, the average loss of the model over all iterations:

$$\frac{1}{N_\text{cycle}} \sum_{c=1}^{N_\text{cycle}} \text{loss}(\text{outputs}_c) \tag{48}$$

where $\text{outputs}_i$ are the model's outputs at iteration $c$. Next, we construct an unbiased Monte Carlo estimate of this objective by uniformly sampling the number of iterations $N'$ between 1 an $N_{\text{cycle}}$, and only train the loss for that step. This means that any following iterations can be skipped. The random choice of the number of iterations $N'$ is synchronized across the batch (i.e. every batch element trains the same iteration on each step) to increase efficiency. Finally, we stop the gradients flowing from the chosen iteration to the previous ones, allowing to skip the backward pass (backpropagation) for the previous iterations $c < N'$. As an example, if we sampled $N' = 3$, and $N_{\text{cycle}} = 4$, then we perform just the forward pass for the first two iterations; both the forward and the backward pass for the third iteration; and the fourth iteration is completely skipped.

We now explain the motivation behind this procedure, compared to simple unrolling. Randomly sampling the iteration $N'$ improves the efficiency, since we now perform $\frac{1}{N_{\text{cycle}}} \sum_{c=1}^{N_{\text{cycle}}} c = \frac{N_{\text{cycle}}+1}{2}$ iterations on average instead of $N_{\text{cycle}}$. Importantly, it also acts as auxiliary loss for the model, requiring to provide plausible outputs mid-way through the inference. We hypothesize that this allows the network to refine its own predictions multiple times. Stopping the gradients of the intermediate outputs makes the procedure much cheaper, both memory-wise and computationally. Memory-wise, it allows to eschew storing the intermediate activations of the previous iterations. Computationally, this procedure requires a single backward pass and $N'$ forward passes. With rematerialization (see subsubsection 1.11.8), a forward pass is approximately 4 times cheaper than the full forward and backward round, thus each forward pass adds only 25% of the baseline training time. Therefore, training a model with $N_{\text{cycle}}$ iterations has an additional average cost of $(12.5\,(N_{\text{cycle}} - 1))\%$. For $N_{\text{cycle}} = 4$ used in AlphaFold, this corresponds to just 37.5% extra training time, compared to 300% with unrolling. While stopping the gradients between iterations leads to a bias in the gradients, we find that does not hamper training.

In AlphaFold, we recycle the predicted backbone atom coordinates from the Structure module (Algorithm 20), and output pair and first row MSA representations from the Evoformer (Algorithm 6). Algorithm 32 shows their embedding details. Both types of representations are passed through LayerNorm to prepare updates for the corresponding input representations $\{\mathbf{z}_{ij}\}$ and $\{\mathbf{m}_{1i}\}$. Predicted coordinates of beta carbon atoms (alpha carbon for glycine) are used to compute pairwise distances, which are then discretized into 15 bins of equal width 1.25Å that span the range to approx. 20Å (Due to historical reasons the precise bin values range from $3\frac{3}{8}$ Å to $21\frac{3}{8}$ Å). The resulting one-hot distogram is linearly projected and added to the pair representation update. The recycling updates are injected to the network as shown in line 6 of Algorithm 2 (for a visual scheme, see R sign in Suppl. Fig. 1). This is the only mechanism employed for recycling previous predictions, and the rest of the network is identical on all recycling iterations.

**Algorithm 32** Embedding of Evoformer and Structure module outputs for recycling

**def** $\text{RecyclingEmbedder}(\{\mathbf{m}_{1i}\}, \{\mathbf{z}_{ij}\}, \{\vec{\mathbf{x}}_i^{\mathrm{C}^\beta}\})$ :

*# Embed pair distances of backbone atoms:*

$d_{ij} = \left\| \vec{\mathbf{x}}_i^{\mathrm{C}^\beta} - \vec{\mathbf{x}}_j^{\mathrm{C}^\beta} \right\|$ $\qquad$ $C^\alpha$ used for glycin

$\mathbf{d}_{ij} = \text{Linear}(\text{one\_hot}(d_{ij}, \mathbf{v}_{\text{bins}} = [3\frac{3}{8}\,\text{Å},\ 5\frac{1}{8}\,\text{Å},\ \ldots, 21\frac{3}{8}\,\text{Å}]))$ $\qquad$ $\mathbf{d}_{ij} \in \mathbb{R}^{c_z}$

*# Embed output Evoformer representations:*

$\tilde{\mathbf{z}}_{ij} = \mathbf{d}_{ij} + \text{LayerNorm}(\mathbf{z}_{ij})$

$\tilde{\mathbf{m}}_{1i} = \text{LayerNorm}(\mathbf{m}_{1i})$

**return** $\{\tilde{\mathbf{m}}_{1i}\}, \{\tilde{\mathbf{z}}_{ij}\}$

## 1.11 Training and inference details

**Table 4** | AlphaFold training protocol. We train each stage until convergence with the approximate timings and number of samples provided.

| **Model** | **Initial training** | **Fine-tuning** |
|---|---|---|
| Number of templates $N_{\text{templ}}$ | 4 | 4 |
| Sequence crop size $N_{\text{res}}$ | 256 | 384 |
| Number of sequences $N_{\text{seq}}$ | 128 | 512 |
| Number of extra sequences $N_{\text{extra\_seq}}$ | 1024 | 5120 |
| Parameters initialized from | Random | Initial training |
| Initial learning rate | $10^{-3}$ | $5 \cdot 10^{-4}$ |
| Learning rate linear warm-up samples | 128000 | 0 |
| Structural violation loss weight | 0.0 | 1.0 |
| Training samples ($\cdot 10^6$) | $\approx 10$ | $\approx 1.5$ |
| Training time | $\approx 7$ days | $\approx 4$ days |

### 1.11.1 Training stages

The training proceeds in two stages outlined in Table 4. We train with sequence crop size 256 and then fine-tune with the larger crop size 384, where the second stage starts from the pre-trained model weights and uses the lower learning rate and no warm-up steps. Additionally, we use the increased number of MSA clusters as well as unclustered (extra) sequences. We also apply the structural violation loss.

### 1.11.2 MSA resampling and ensembling

There are several sources of stochastic behaviour in the network. The most important one is MSA pre-processing, which includes MSA block deletion (Algorithm 1) and MSA clustering procedure (subsubsection 1.2.7). To reduce the stochastic effects of these steps, we perform them multiple times, resulting in a set of sampled MSA and extra MSA features. Note that the residue cropping (subsubsection 1.2.8), which follows these steps, is done consistently across all samples, i.e. the same residue ranges are taken. Also note that we do not perform template re-sampling, i.e. the same templates (up to 4) are used for one training example.

To take advantage of the multiple samples of these features, we embed them multiple times with the Evoformer network and average the output embeddings. Concretely, at each recycling iteration we perform multiple passes of the Evoformer (including all preceding input embedding transformations) for the different instances of ”msa_feat” and ”extra_msa_feat”, and we average the output pair and single representations before providing them to the structure module or any other heads of the network (see Algorithm 2). We call this technique ensembling (although it does not ensemble final predictions) and we employ it during inference, but not during training. Finally, since we also use different samples of the MSA and extra MSA features at each recycling iteration, the full system processes the total of $N_{\text{cycle}} \times N_{\text{ensemble}}$ samples.

We use $N_{\text{ensemble}} = 3$ for the best checkpoint selection in the evaluator (subsubsection 1.11.7) and also for the inference on CASP14 targets performed for this paper. However, with the recycling and pLDDT-based selection from 5 model runs, we have found that the additional ensembling has only a minor impact on the accuracy of the system (see subsubsection 1.12.2 for details). Given that $N_{\text{ensemble}}$ is almost a linear multiplier for the inference time, we are gradually deprecating it in our systems, and the inference on the recent PDB dataset for this paper has been performed with $N_{\text{ensemble}} = 1$.

### 1.11.3 Optimization details

For optimization we use Adam [106] with the base learning rate $10^{-3}$, $\beta_1 = 0.9$, $\beta_2 = 0.999$, $\epsilon = 10^{-6}$. We linearly increase (warm-up) the learning rate over the first $0.128 \cdot 10^6$ samples, and further decrease the learning rate by a factor of $0.95$ after $6.4 \cdot 10^6$ samples. During the fine-tuning stage we have no learning rate warm-up, but we reduce the base learning rate by half.

We train using the mini-batch size of $128$, one example per TPU-core. To stabilize the training, we apply gradient clipping by the global norm [107] on the parameters independently to every training example in a mini-batch, with a clipping value $0.1$.

### 1.11.4 Parameters initialization

**Linear layers.** By default, the weights of the Linear layers are initialized using the LeCun (fan-in) initialization strategy [108] with a truncated normal distribution. By default, the biases of the Linear layers are initialized by zero. For the layers immediately followed by a ReLU activations, we use He initializer [109]. The queries, keys and values projection layers in self-attention layers are initialized using the 'fan-average' Glorot uniform scheme [110].

To improve stability of training, we zero-initialize the weights of the *final* weight layers [111] in every residual layer. This ensures that every residual layer acts as an identity operation at initialization. Furthermore, we zero-initialize all the final projection weight layers of the network: masked MSA prediction logits, residue distance prediction logits, model confidence prediction logits. We also identity-initialize the rigid frame updates in the Structure module.

**Gating Linear layers,** i.e. the Linear layers immediately followed by a $\mathrm{sigmoid}$, are initialized with zero weights. The biases are initialized with a constant value of $1$, ensuring that at initialisation, the gates are mostly-opened with a value of $\mathrm{sigmoid}(1) = 1/(1 + \exp(-1)) \approx 0.73$.

**Layer normalization** layers are initialized using the standard scheme: gains set to $1$ and biases set to $0$.

**Point weights in Invariant Point Attention** $\gamma^h$ are initialized to be $1$ after the softplus transformation.

### 1.11.5 Loss clamping details

In $90\%$ of training mini-batches the FAPE backbone loss is clamped by $e_{\max} = 10$ Å, in the remaining $10\%$ it is not clamped, $e_{\max} = +\infty$. For side-chains it is always clamped by $e_{\max} = 10$ Å.

### 1.11.6 Dropout details

We denote the standard Dropout [97] with operator $\mathrm{Dropout}_x$, where $x$ is the dropout rate, i.e. the probability to zero an entry. In residual updates for the self-attention operations, we modify it such that dropout masks are shared across one of the dimensions. We use $\mathrm{DropoutRowwise}_x$ operator to denote the version with the mask of shape $[1, N_{\text{res}}, N_{\text{channel}}]$, which is shared across rows. It is used in the residual updates following the MSA row-wise self-attention and the triangular self-attention around starting node. For a given residue (column) the same channels are set to zero across all rows in these updates. Similarly, we use $\mathrm{DropoutColumnwise}_x$ operator to denote the version with the mask of the shape $[N_{\text{res}}, 1, N_{\text{channel}}]$, which is shared across columns. It is used in the residual update following the triangular self-attention around ending node. In the main Evoformer stack we also apply the row-wise Dropout when performing the residual updates for the triangular multiplicative operations in the pair stack. We keep the same configuration for the unclustered MSA stack and the Template pair stack. In the Structure module, we apply Dropout to the results of the Invariant Point Attention and the Transition layer. Please refer to Algorithm 6, Algorithm 16, Algorithm 18, and Algorithm 20 for the exact locations of all Dropout operations and their rates.

### 1.11.7 Evaluator setup

During evaluation, we use an exponential moving average of the trained parameters [112] with the decay 0.999. To select the best model during training, we monitor lDDT-C$\alpha$ performance on a validation set of targets collected from CAMEO [113] over 3 months period (2018-12-14 to 2019-03-09). It is filtered to a maximum sequence length of 700, and there is no residue cropping at evaluation time.

### 1.11.8 Reducing the memory consumption

AlphaFold model has high memory consumption, both in training and during inference. This is largely driven by the logits in the self-attention layers and the large pair and MSA activiations. For example, $a^h_{ijk}$ in Algorithm 13 and Algorithm 14 consists of $(N^3_{\text{res}} \cdot N_{\text{head}})$ floating point numbers. We now describe the techniques we employ to perform memory-efficient training and inference of this model.

**Training.** We store all intermediate activations of the model in `bfloat16` format, which only requires two bytes per floating point number. Training is performed on relatively short sequence crop sizes, up to $N_{\text{res}} = 384$. The cubic scaling of the self-attention logits makes it impractical to store them all for backpropagation, as is done in naive backpropagation. Specifically, storing just the attention logits of the largest sublayer type for all blocks requires more memory than the 16 GiB available on TPUv3 core:

$$\underset{N^3_{\text{res}}}{384^3} \cdot \underset{N_{\text{head}}}{4} \cdot \underset{N_{\text{block}}}{48} \cdot \underset{\text{bytes per bfloat16}}{2} = 20.25 \text{ GiB}. \tag{49}$$

To solve this, we leverage a standard technique called gradient checkpointing, or rematerialization [114, 115]. Concretely, during the forward pass, we store the activations that are passed between the $N_{\text{block}} = 48$ Evoformer blocks. During the backward pass, we recompute all activations within the blocks. This means we need to store the logits for only one layer at a time, bringing down the memory consumption to just 0.4 GiB. Storing the intermediate pair activations requires an extra

$$\underset{N^2_{\text{res}}}{384^2} \cdot \underset{c_z}{128} \cdot \underset{N_{\text{block}}}{48} \cdot \underset{\text{bytes per bfloat16}}{2} = 1.7 \text{ GiB}. \tag{50}$$

Rematerialization's computational cost is equivalent to an extra forward pass through the network. Empirically, the backward pass is twice as expensive as the forward pass, so rematerialization increases the training time by 33%.

**Inference.** The intermediate activations are stored in `float32` format, requiring four bytes per floating point number. During inference, we do not need to store the intermediate activations, which reduces the memory consumption. However, we also consider proteins with dramatically larger $N_{\text{res}}$ than during training. For example, T1044 has $N_{\text{res}} = 2180$, so a single layer's logits have the size of

$$\underset{N^3_{\text{res}}}{2180^3} \cdot \underset{N_{\text{head}}}{4} \cdot \underset{\text{bytes per float32}}{4} = 154.4 \text{ GiB}, \tag{51}$$

which again exceeds the memory size of modern accelerators. To reduce this burden, for each type of layer in the network, we identify a 'batch-like' dimension where the computation is independent along that dimension. We then execute the layer one 'chunk' at a time, meaning that only the intermediate activations for that chunk need to be stored in memory at a given time. Thus, reducing the chunk size improves memory efficiency, but at a cost of performance, as small chunks cannot fully exploit the parallelism of the hardware. In practice, we often use a chunk size of 4, resulting in much lower memory consumption for the logits:

$$\underset{N^2_{\text{res}}}{2180^2} \cdot \underset{N_{\text{head}}}{4} \cdot \underset{\text{chunk size}}{4} \cdot \underset{\text{bytes per float32}}{4} = 0.3 \text{ GiB}. \tag{52}$$

The same technique was used in a similar context in [116].

## 1.12 CASP14 assessment

### 1.12.1 Training procedure

**Table 5** | Training protocol for CASP14 models. The models in **bold** (i.e. **1.1.1** – **1.2.3**) were used in the assessment. We report the number of training samples and the training time (in days and hours) until the best validation score. Three dots (···) indicate the same value as in the former column.

| | initial training | first fine-tuning | | second fine-tuning | | | | |
|---|---|---|---|---|---|---|---|---|
| Model | 1 | 1.1 | 1.2 | **1.1.1** | **1.1.2** | **1.2.1** | **1.2.2** | **1.2.3** |
| Parameters initialized from | Random | Model 1 | ··· | Model 1.1 | ··· | Model 1.2 | ··· | ··· |
| Number of templates $N_{\mathrm{templ}}$ | 4 | 4 | 0 | 4 | ··· | 0 | ··· | ··· |
| Sequence crop size $N_{\mathrm{res}}$ | 256 | ··· | ··· | 384 | ··· | ··· | ··· | ··· |
| Number of sequences $N_{\mathrm{seq}}$ | 128 | 512 | ··· | ··· | ··· | ··· | ··· | ··· |
| Number of extra sequences $N_{\mathrm{extra\_seq}}$ | 1024 | ··· | ··· | 5120 | 1024 | 5120 | ··· | 1024 |
| Initial learning rate | $10^{-3}$ | $5 \cdot 10^{-4}$ | ··· | ··· | ··· | ··· | ··· | ··· |
| Learning rate linear warm-up samples | 128000 | 0 | ··· | ··· | ··· | ··· | ··· | ··· |
| Structural violation loss weight | 0.0 | 1.0 | ··· | ··· | ··· | ··· | ··· | ··· |
| "Experimentally resolved" loss weight | 0.0 | 0.01 | ··· | ··· | ··· | ··· | ··· | ··· |
| Training samples ($\cdot 10^6$) | 9.2 | 1.1 | 1.7 | 0.3 | 0.6 | 1.4 | 1.1 | 2.4 |
| Training time | 6d 6h | 1d 10h | 2d 3h | 20h | 1d 13h | 4d 1h | 3d | 5d 12h |

We now describe the exact training process for the models used in the CASP14 assessment. The training of the model proceeds in three stages with exact details presented in Table 5. First, we train model 1 with a lower crop size and number of sequences for about a week. Then, we fine-tune this model with more sequences, enabled violation losses, and a lower learning rate. For historical reasons, we also added the "experimentally resolved" predictor (subsubsection 1.9.10) at this stage. We fine-tune two models: model 1.1 uses templates, while model 1.2 does not (the parameters pertaining to templates are dropped in this case). To obtain the final five models, we perform a second round of fine-tuning with larger sequence crop size and more extra MSA sequences from these two models. All CASP14 models are trained with distillation (subsection 1.3) from a slightly earlier version of the model. Empirically, we find that small changes in the model details have little effect on the distillation procedure. The training process, excluding the preparation of the distillation dataset, takes about two weeks. Since the assessment, we have found that the intermediate fine-tuning stage can be omitted without degradation of quality, and we follow the simplified training protocol (Table 4) in our ablation studies.

We continued to improve the models during the CASP14 assessment, thus some of our submissions used slightly different details, and some submissions had manual interventions. For more details please refer to the short description in [117] and also an upcoming publication in "Proteins: Structure, Function, and Bioinformatics". For the accuracy comparison of the systems see subsubsection 1.12.2 below.

### 1.12.2 Inference and scoring

At inference time, the five models are independently executed on the same set of inputs. Then, the predictions are re-ranked according to the chain pLDDT confidence measure. The original CASP14 submissions were inferenced with $N_{\mathrm{ensemble}} = 8$, which has been later reduced in our system.

After the CASP14 assessment we have re-evaluated the final system described in subsubsection 1.12.1 with $N_{\mathrm{ensemble}} = 3$ and obtained a mean domain GDT of 87.65, compared to 87.66 GDT that our actual submissions get on the same set of domains. The median C$\alpha$ RMSD at 95% coverage is 0.96 for both the described system and the actual submissions. Furthermore, the described system without ensembling ($N_{\mathrm{ensemble}} = 1$) obtains GDT 87.56, which is only 0.1 points less than the full system, while yielding 8 times speedup for the inference.

For this evaluation, we are predicting the structure of all non-cancelled, non-server targets as full chains (except T1085 and T1086, for which the structures are embargoed). Note that the long (2180 residues) target T1044 structure has also been predicted as a whole chain and then split into the domain targets (T1031, T1033, T1035, T1037, T1039–T1043) for the evaluation. We used all FM, TBM-easy, TBM-hard and FM/TBM domains (87 in total):

`T1024-D1, T1024-D2, T1026-D1, T1027-D1, T1029-D1, T1030-D1, T1030-D2, T1031-D1, T1032-D1, T1033-D1, T1034-D1, T1035-D1, T1037-D1, T1038-D1, T1038-D2, T1039-D1, T1040-D1, T1041-D1, T1042-D1, T1043-D1, T1045s2-D1, T1046s1-D1, T1046s2-D1, T1047s1-D1, T1047s2-D1, T1047s2-D2, T1047s2-D3, T1049-D1, T1050-D1, T1050-D2, T1050-D3, T1052-D1, T1052-D2, T1052-D3, T1053-D1, T1053-D2, T1054-D1, T1055-D1, T1056-D1, T1057-D1, T1058-D1, T1058-D2, T1060s2-D1, T1060s3-D1, T1061-D1, T1061-D2, T1061-D3, T1064-D1, T1065s1-D1, T1065s2-D1, T1067-D1, T1068-D1, T1070-D1, T1070-D2, T1070-D3, T1070-D4, T1073-D1, T1074-D1, T1076-D1, T1078-D1, T1079-D1, T1080-D1, T1082-D1, T1083-D1, T1084-D1, T1087-D1, T1089-D1, T1090-D1, T1091-D1, T1091-D2, T1091-D3, T1091-D4, T1092-D1, T1092-D2, T1093-D1, T1093-D2, T1093-D3, T1094-D1, T1094-D2, T1095-D1, T1096-D1, T1096-D2, T1099-D1, T1100-D1, T1100-D2, T1101-D1, T1101-D2.`

We use this set for all CASP14-related results reported in the paper.

## 1.13 Ablation studies

### 1.13.1 Architectural details

We estimate the relative importance of key components of the architecture by training and evaluating a number of ablation models:

**Baseline.** Baseline model as described in the paper without noisy-student self-distillation. All other ablations should be understood relative to this baseline model.

**With self-distillation training.** Full model as described in the paper including noisy-student self-distillation training.

**No templates.** We remove the template stack and the template torsion angle features, see subsubsection 1.7.1 and Suppl. Fig. 1. The number of MSA clusters $N_{\text{clust}}$ is increased by $N_{\text{templ}} = 4$ to keep the overall size of the MSA representation.

**No raw MSA (use MSA pairwise frequencies).** AlphaFold builds representations by performing attention on individual MSA sequences. Here we ablate whether this is important and modify the system to provide only first and second order MSA statistics.

We denote the raw MSA data as $M$, where $M_{si}$ is the amino acid of sequence $s$ at position $i$. We also denote the total number of sequences as $S$. We define the MSA profile for position $i$ as the empirical distribution of amino acids occurring at this position:

$$p_i(A) = \frac{1}{S}\sum_{s=1}^{S}[M_{si} = A], \tag{53}$$

where $[\,]$ denotes the Iverson bracket and is equal to 1 if the expression inside is true and 0 otherwise. Furthermore we define the pairwise frequency table as the probability of observing amino acid A at position $i$ and amino acid B at position $j$:

$$f_{ij}(A, B) = \frac{1}{S}\sum_{s=1}^{S}[M_{si} = A][M_{sj} = B]. \tag{54}$$

We note that these type of MSA features are commonly used in the literature, e.g. by the trRosetta pipeline [118] (with further down-weighting of redundant sequences in the averages). Usually these first and second order statistics are transformed by computing the inverse covariation matrix or fitting a statistical model like

a Potts Model before the features are passed to the network. Here we will just use the raw features without sequence nonlocal preprocessing and allow the model to process them as needed.

This ablation will have access to the following MSA information:

- MSA profile, i.e. the marginal distribution $p_i(A)$;
- Expected number of deletions for a given position;
- MSA pairwise frequency table $f_{ij}(A, B)$;
- Second order statistics for the number of deletions at position $i$ and amino acid types at position $j$;
- Second order statistics for the number of deletions at different positions.

We implement this ablation in terms of a set of small interventions in our system. We will show that with these interventions all provided MSA information can be directly derived from the features listed above.

In the data pipeline, we set the number of clusters $N_{\text{clust}} = 1$ so that the MSA representation only has a single row. The first cluster is always set to the sequence of the modelled protein. Furthermore in the clustering stage all sequences in the MSA will be assigned to this single cluster, this means the cluster profile feature is just the full MSA profile.

We also remove the template torsion angle features that otherwise would be concatenated as additional rows. Since columnwise MSA attention would be trivial in this setup, we remove this operation from the Evoformer stack.

Furthermore we replace the extra MSA stack (Algorithm 18) with a single outer product mean (Algorithm 10) on the extra MSA features $\{\mathbf{f}^{\text{extra\_msa\_feat}}_{s_e i}\}$ and use it to initialize the pair representation.

We denote the set of features (MSA sequences and deletions) usually passed to the extra MSA stack by $\mathbf{f}_{si}$ and analyse what information of the MSA can be accessed by the model. Applying the outer product mean operation on $\mathbf{f}_{si}$ yields linear projections of the following kind of terms: $\left\{\sum_s \mathbf{f}_{si} \otimes \mathbf{f}_{sj}, \sum_s \mathbf{f}_{si}, \sum_s \mathbf{f}_{sj}\right\}$. The first kind of terms comes from the outer product on the linearly projected features, the other two come from the mixed bias and features terms in the outer product, since we use linear transformations with a bias. When writing out the MSA features explicitly, we see that the first term contains the pairwise frequencies. Additionally, we have the second order statistics involving deletions. The other two terms result in an outer sum of profiles and expected number of deletions.

**No triangles, biasing, or gating (use axial attention).** First, we remove the triangle inductive bias. For this we replace the pair bias $b^h_{ij}$ in Algorithm 13 and Algorithm 14 with a projection of the relative distances along the chain, i.e. $\text{relpos}_{ij}$ from Algorithm 4. We also replace the two triangular multiplicative updates (Algorithm 11 and Algorithm 12) with two self-attention layers identical to the ones described above.

Furthermore, we replace the pair bias in the row-wise MSA attention Algorithm 7 with the projected relative distances $\text{relpos}_{ij}$ and remove the gating in all attention operations. After these modifications, the attention used in the main part of the model closely matches the standard axial (or criss-cross) attention [119].

These modifications also imply that the pair representation does not influence the MSA representation directly. Though it is important to note that we keep Algorithm 10 without any modifications, so the MSA representation still influences the pair representation. We also do not modify the structure module, as such the pair representation still participates in building the structure.

**No recycling.** We remove recycling (see subsection 1.10) during training and inference, i.e. we only make a single pass through the model.

**No invariant point attention (use direct projection).** We replace the prediction of backbone frames, including all of the IPA Algorithm 22, with a linear direct projection of the backbone frame similar to Algorithm 23. The direct projection consists of linear layer applied to the final Evoformer single embedding to produce a $N_{\text{res}} \times 7$ matrix. The seven coordinates are converted into a backbone frame in the following way: The first 4 coordinates $(q_w, q_x, q_y, q_z)$ are converted to the rotation matrix by creating a normalized quaternion

$$(a, b, c, d) = (1 + q_w, q_x, q_y, q_z) / \sqrt{(1 + q_w)^2 + q_x^2 + q_y^2 + q_z^2}\,, \tag{55}$$

and applying the standard formula for converting a quaternion to a rotation matrix:

$$R = \begin{pmatrix} a^2+b^2-c^2-d^2 & 2bc-2ad & 2bd+2ac \\ 2bc+2ad & a^2-b^2+c^2-d^2 & 2cd-2ab \\ 2bd-2ac & 2cd+2ab & a^2-b^2-c^2+d^2 \end{pmatrix}. \quad (56)$$

The last 3 predicted coordinates $(t_x, t_y, t_z)$ are used as the translation vector. We also apply the side chain torsion module directly to the single embedding in the same manner as in the full model. In this ablation, there is no iterative process in the Structure module. Additionally, the final pair representation is not used in any way in the Structure module in this ablation but is used for the distogram loss.

**No invariant point attention and no recycling.** This ablation simply combines the changes of the two individual ablations described above.

**No end-to-end structure gradients (keep auxiliary heads).** We stop the gradients from the structure module into the main part of the network (i.e. the structure losses have no effect on Evoformer parameters), which means the Evoformer embeddings are trained only using the distogram loss and other auxiliary losses.

**No auxiliary distogram head.** We remove the auxiliary loss that predicts distograms (see subsubsection 1.9.8).

**No auxiliary masked MSA head.** We remove the auxiliary BERT-style loss that imputes the masked values in the MSA (see subsubsection 1.9.9). The ablation still uses the same MSA processing, i.e. the MSA is still randomly masked.

### 1.13.2 Procedure

**Models training.** For all ablations we kept hyperparameters from the main model configuration, which we have not re-tuned. We trained the models in two stages following the main training protocol shown in Table 4. All models apart from one ablation were trained without the distillation dataset. Including examples from the full AlphaFold distillation dataset would mean leaking architectural choices of the models that were used to create this dataset, while re-creating the dataset for each individual ablation would be too computationally expensive. For each ablation we trained 3 or 4 identical models with different random seeds and discarded any unstable runs using the validation set from subsubsection 1.11.7.

**Datasets and metrics.** We used two different test sets for ablations analysis. Firstly, we used the set of CASP14 domains described in subsubsection 1.12.2 and evaluated individual domain predictions with the Global Distance Test (GDT) [120]. Secondly, we used the redundancy-reduced set of recent PDB chains described in Methods, further restricted to protein chains with template coverage $\leq$ 30% at 30% identity ($N = 2261$ targets). We scored the full chain predictions with lDDT-C$\alpha$ [99].

### 1.13.3 Results

Ablation results are presented in Fig. 4b of the main paper. We show the difference in mean backbone accuracy for each ablation relative to an average of baseline seeds using both the CASP14 targets and the redundancy- and template-reduced set from recent PDB. Confidence intervals for each training seed are given by bootstrap over domains for CASP and full chains for PDB. Additionally, in Suppl. Fig. 10 we find that the different ablations have quite non-uniform dependence on the MSA depth. Some ablations like No Masked MSA head primarily affect shallow MSAs whereas others such as No Recycling have effects at all MSA depths.

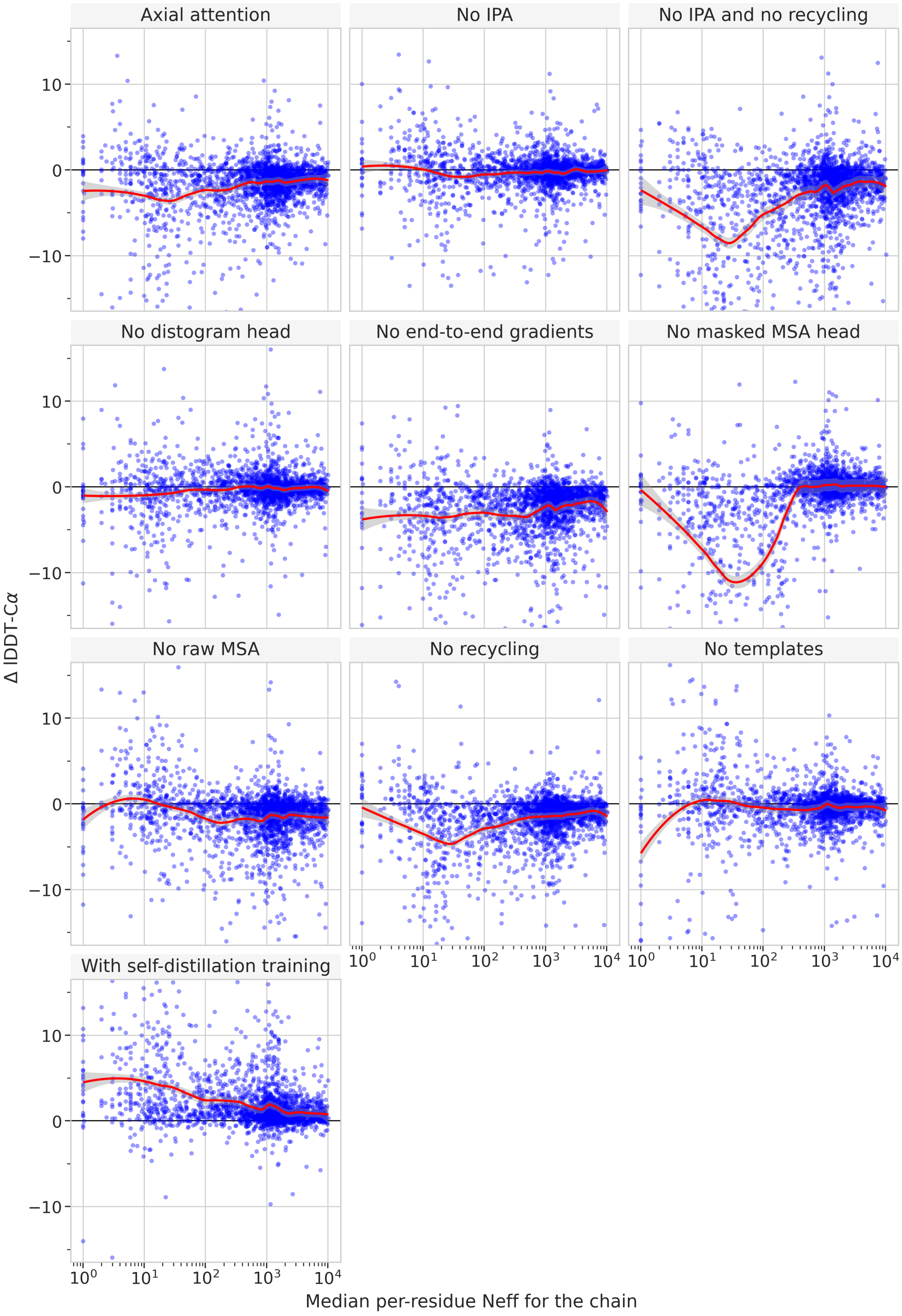


**Supplementary Figure 10** | Ablations accuracy relative to the baseline for different values of the MSA depth on the recent PDB set of chains, filtered by template coverage $\leq 30\%$ at $30\%$ identity ($N = 2261$ protein chains). MSA depth is computed by counting the number of non-gap residues for each position in the MSA (using the Neff weighting scheme with a threshold of 80% identity measured on the region that is non-gap in either sequence) and taking the median across residues. Plots are restricted to the range [-15, 15]; the red lines represent LOESS mean estimates with 95% confidence intervals for the LOESS.

The presented ablations remove relatively independent components of the system, such as end-to-end training, iterative structure refinement, triangular inductive bias, and others from the no-self-distillation baseline. While this analysis helps determine the relative importance of different ideas in the model, there are two major limitations. First, hyperparameters were not re-tuned when performing the ablations which could make ablations appear more significant than in a properly tuned model. Second, interactions between different components may be strongly non-linear as the components of the AlphaFold system can have overlapping or interacting roles. As a dramatic example, removal of Invariant Point Attention (IPA) from the model has only a tiny effect on accuracy for recent PDB, but removing both recycling and IPA from the model has a much larger effect than removing recycling alone. This double-ablation must be interpreted with caution, however, as it is not clear which missing features of recycling (embedding of intermediate structures, much deeper network, intermediate losses, or weight tying) explain the severe ablation.

In general, the ablations confirm that many architecture and training features have an effect on final AlphaFold accuracy and the relative magnitude of effects are broadly consistent whether measured on the human-curated CASP set or the much larger recent PDB set.

The strong contributions of self-distillation training and masked MSA losses suggest that these techniques are making effective use of unlabelled data within the supervised training framework (and despite the lack of unsupervised pre-training). The various architecture ablations confirm that many different architectural innovations contribute to the final AlphaFold accuracy. Finally, careful handling of the structure and intermediate losses (using both end-to-end structure gradients and providing intermediate loss gradients in recycling) is important to achieving full accuracy. This is likely due to pushing the network towards having a concrete representation of the structure as appears to be present in the trajectories of intermediate structure predictions.

A surprisingly small role is played by the distogram head, suggesting that it is not a necessary output to obtain high-accuracy structures. Another surprise is that equivariance in the network is not essential as the single ablation of IPA, which removes all equivariant components, is still a very accurate structure prediction network. Similarly, the single ablation of raw MSA leads to a drop in accuracy but it is not catastrophic. We hypothesize though that each of these features would show much larger effects within multiple ablations similar to the effect seen in ablating both IPA and recycling.

## 1.14 Network probing details

This paper includes supplementary videos with structures evolving over $4$ recycling iterations and $48$ Evoformer blocks. Static versions of those videos are also presented as GDT curves per $4 \cdot 48 = 192$ intermediate points and discussed in the main part of the paper. In this section we provide additional details about how these results were produced.

We started with a full network architecture and added additional probing modules after each Evoformer block. Concretely, we incorporated copies of the Structure module (subsection 1.8) with the same architecture and configuration and optimized the probing loss $\mathcal{L}_{\text{probe}}$, defined as the average of the losses of these modules. For inputs, we provided the current versions of the MSA and pair representations produced up to this point in the network. As with the main network, we had different trainable parameters across Evoformer blocks, but tied across recycling iterations. In other words, we trained $48$ additional sets of weights for these modules. Note that we have not attached any probing modules to the template pair stack or the extra MSA stack. Importantly, when training the probing network, we stopped all gradients coming to the main network parameters both from the probing and the main losses. Outputs of the probing modules have not been recycled in any way, i.e. they were used for the analysis only.

In the presented analysis, we were probing one of the five CASP14 models (model 1.1.2 from Table 5 to be precise). We used the same data sizes as in this model, i.e. $N_{\text{templ}} = 4$, $N_{\text{res}} = 384$, $N_{\text{seq}} = 512$, and $N_{\text{extra\_msa}} = 1024$. For the learning rate, we used the standard schedule from our training stage (see subsubsection 1.11.3). Following the main training stage setup, we have not used violation losses (subsubsection 1.9.11) for the probing. In the evaluator (subsubsection 1.11.7) we used the validation probing loss $\mathcal{L}_{\text{probe}}$ for the model selection and we changed the exponential moving average decay to $0.99$ for a faster training

startup. We trained the probing network for about 5 days until convergence. We used $N_{\text{ensemble}} = 1$ both for the build-in evaluator and for the presented inference to simplify setup and reduce memory usage.

### 1.15 Novel fold performance

Across CASP14 and CASP_Commons, five of the assessed targets were sufficiently distinct from prior PDB entries to be called novel folds. Targets T1035, T1037, T1040 and T1042 (PDB:6vr4) were highlighted as new folds by the CASP14 assessors [121], while C1905 (PDB:6xdc) is called novel in its primary citation [122]. Here we evaluate AlphaFold's performance on an expanded test set of recent structures selected for their novelty.

Three heuristics were used to identify recent PDB chains that might be novel with respect to AlphaFold's training set:

1. The PDB abstract or structure title contains the phrase "novel fold".
2. The chain contains a Pfam domain from a clade not associated with any structure released before 2018-04-30.
3. The chain maps to a SCOP fold not associated with any structure released before 2018-04-30.

The SIFTS resource was used to map PDB chains to Pfam and SCOP [123], with clade and fold annotations downloaded from the respective project websites [124, 125]. Where multiple similar structures were surfaced from the same study only one was kept, and trivial structures consisting of a single helix were also discarded. Where it was clear from the literature that a specific domain was novel, or where the chain consisted of multiple domains joined by an unresolved linker, the structure was cropped to a more reasonable unit for TM-align comparison. The final test set was selected by TM-align comparison against all chains in the training set [104]. Any target with a TM-score greater than 0.6 was eliminated (normalising with respect to the length of the candidate novel structure). Of the remaining 24 targets, 15 have a TM-score of less than 0.5 to the closest training set chain.

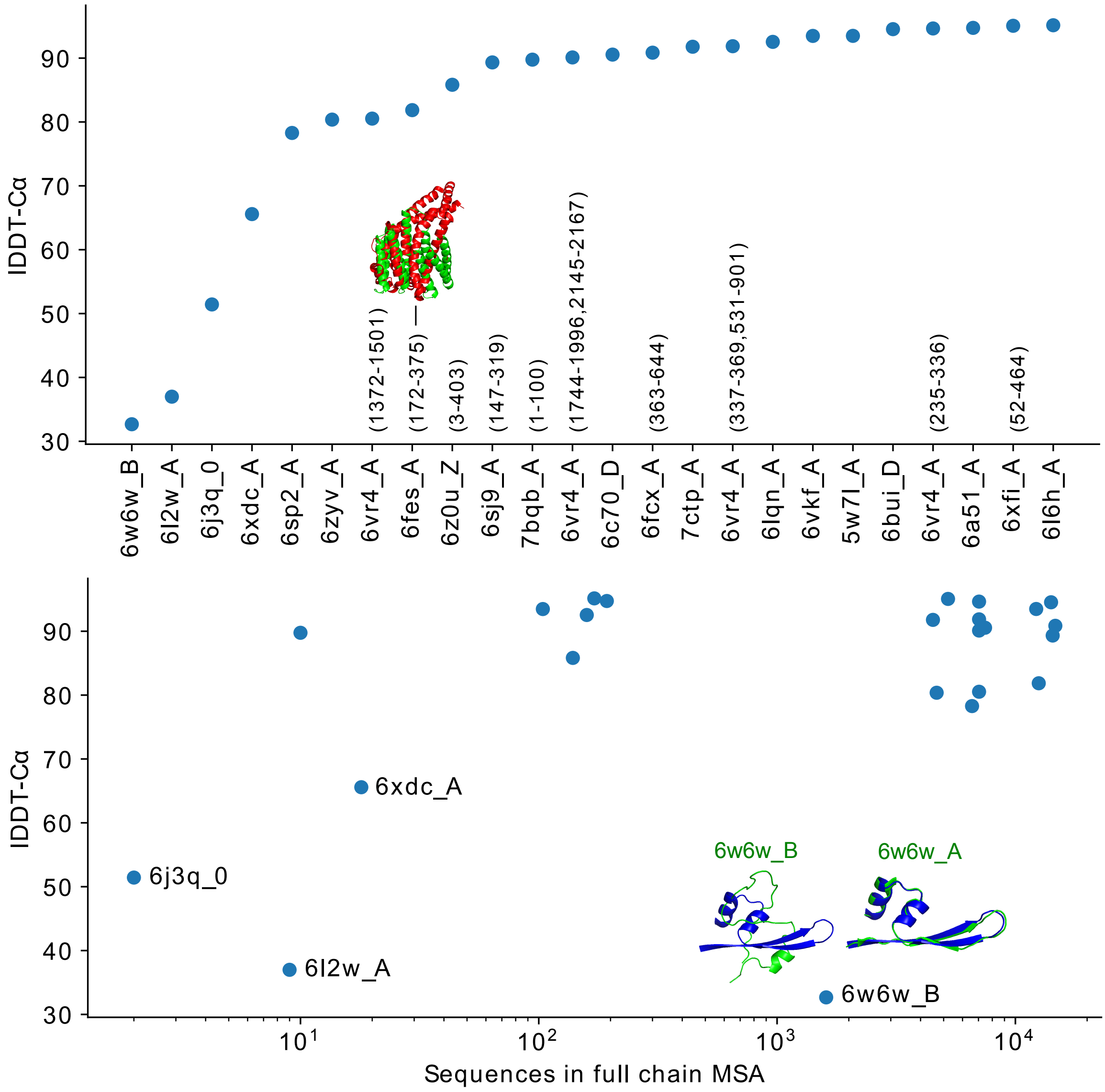


**Supplementary Figure 11** | Performance on a set of novel structures ($N = 24$ protein chains). Where the target is not a full chain, the evaluated region is given as residue ranges in PDB numbering. 6fes_A has the highest TM-score to a training set chain (0.57); the structure comparison shows this target (green) aligned to its closest match with cealign [126] (5jxf_B, red, cropped for easier comparison). The bottom graph shows the relationship between performance and full chain MSA depth, with low lDDT-C$\alpha$ outliers labelled. For outlier 6w6w_B, we also show the prediction (blue) aligned to two possible ground truth structures (green): 6w6w_B and the corresponding subsequence of 6w6w_A which strongly suggests that AlphaFold has simply produced an alternative conformation of this sequence.

Prediction used $N_{\text{ensemble}} = 3$ and disallowed templates released after 2018-04-30, but otherwise followed our CASP14 procedure. The input to AlphaFold was the full chain fasta sequence for each target, extracted from the relevant mmCIF file. Figure 11 shows the results. The median lDDT-C$\alpha$ on this set was 90.3, with mean lDDT-C$\alpha$ 82.6. Investigating the outliers, we found that almost all could be explained by a small MSA containing fewer than 20 sequences. In the case of 6w6w_B, the ground truth structure may be an alternate conformation adopted by this subsequence in complex. The AlphaFold prediction closely matches the conformation seen in 6w6w_A, which forms part of a larger domain. We conclude that AlphaFold is able to predict novel structures well, subject to the same constraints described elsewhere in this work (a sufficiently large MSA, and minimal dependence of the conformation on missing context from surrounding chains).

## 1.16 Visualization of attention

In this section we are going to analyse a small subset of the attention patterns we see in the main part of the model. This will be restricted to relatively short proteins with fairly shallow MSA's in order for easier visualization.

We are going to examine the attention weights in the attention on the pair representation in the "Triangular gated self-attention around starting node" (Algorithm 13). The attention pattern for a given head $h$ is a third order tensor $a^h_{ijk}$ (line 5 of Algorithm 13), here we will investigate different slices along the $i$ axis as well as averages along this axis, and display the $jk$ array as a heat map. We specify the attention pattern for a specific head by the recycling index $r$, layer index $l$ and head index $h$. We show the attention patterns in Suppl. Fig. 12

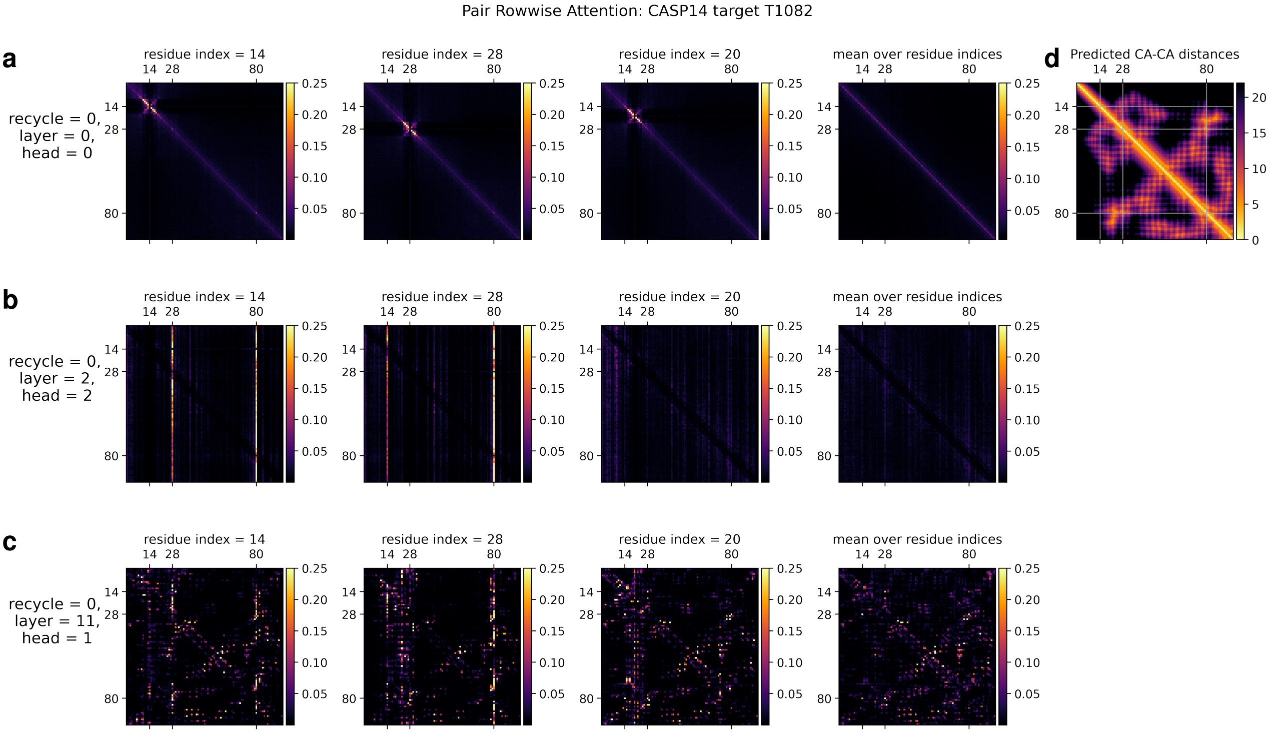


**Supplementary Figure 12** | Visualization of row-wise pair attention. (**a**) Attention patterns in layer 0, head 0. (**b**) Attention patterns in layer 2, head 2. (**c**) Attention patterns in layer 11, head 1. (**d**) Predicted C$\alpha$ – C$\alpha$ distances.

In layer 0, head 0 (Suppl. Fig. 12a) we see a pattern that is similar to a convolution, i.e. the pair representation at $(i, i + j)$ attends to the pair representation $(i, i + k)$, where j and k are relatively small and the pattern is fairly independent of i. The radius of the pattern is 4 residues, we note that the hydrogen bonded residues in an alpha helix are exactly 4 residues apart. Patterns like this are expected in early layers due to the relative position encoding, however one would naively expect it to be wider, as the relative positions run to 32.

In layer 2, head 2 (Suppl. Fig. 12b) we see a very specific attention pattern. We see that positions $14, 28$ and $80$ play a special role. These correspond to the positions of cysteine residues in the protein. We see that at the cysteine positions the attention is localized to the other cysteine positions as exemplified by the bands in the first two panels. Meanwhile away at positions different from the cysteine like position 20, we see that the attention is devoid of features suggesting that the model does not use the attention at these positions. We found this behaviour to be consistent across different positions and proteins, this possibly indicates finding possible disulfides as a key feature of some heads.

A head later in the network (Suppl. Fig. 12c) shows a rich, non-local attention pattern resembling the

distance between pairs in the structure (Suppl. Fig. 12d).

In Suppl. Fig. 13 we show a visualization of the attention pattern in the MSA along the columns $a^h_{sti}$ (line 4 of Algorithm 8). We slice along the last axis $i$ and display the $st$ array as heat map.

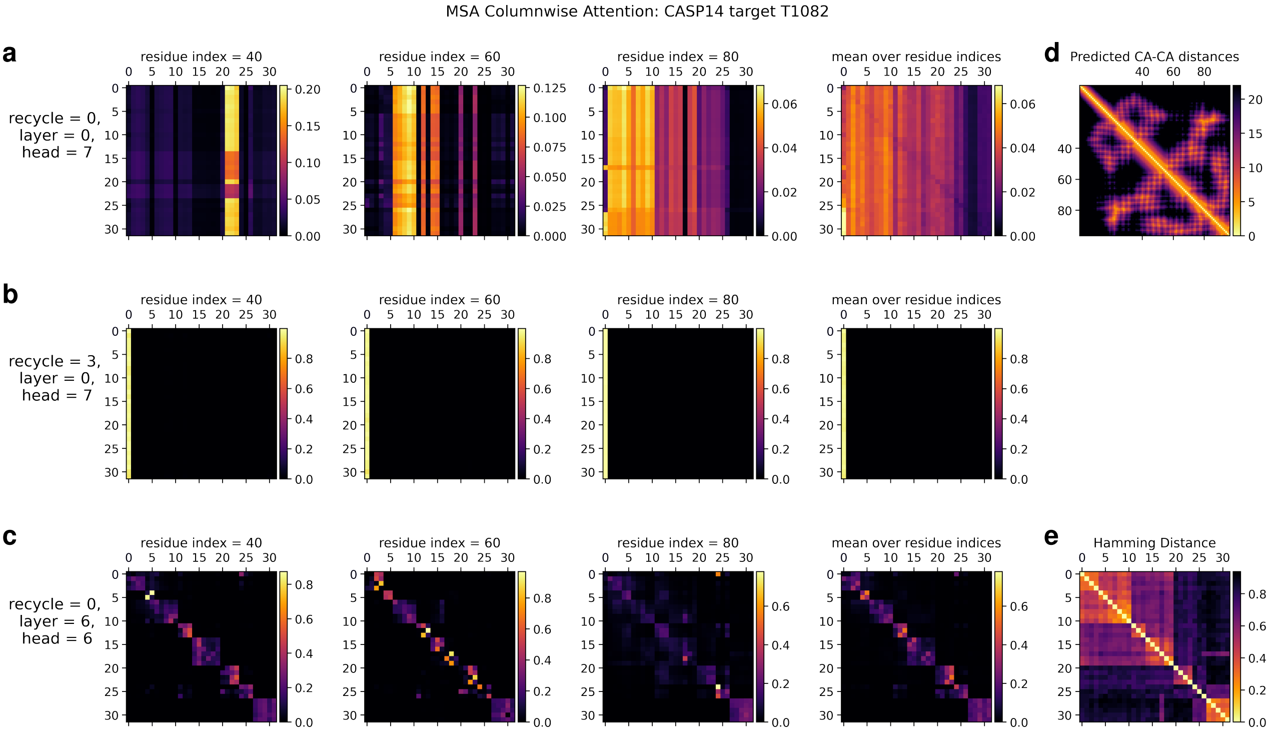


**Supplementary Figure 13** | Visualization of attention in the MSA along sequences. (**a**) Attention patterns in layer 0, head 7. (**b**) Attention patterns in layer 0, head 7 in the last recycling iteration. (**c**) Attention patterns in layer 6, head 6. (**d**) Predicted C$\alpha$ – C$\alpha$ distances (**e**) Hamming distances between the sequences of the MSA.

The original MSA subset shown to the main part of the model is randomly sampled. As such the order is random except for the first row. The first row is special because it contains the target sequence and is recycled in consecutive iterations of the model. Due to the shallow MSA of this protein, the random subset leads to a random permutation of the sequences. In order to facilitate easier interpretation of the attention patterns here we reorder the attention tensor by using a more suitable order for the MSA. We perform a hierarchical clustering using the Ward method with simple Hamming distance as a metric and use the output to re-index the sequence dimension in the MSA attention tensor. We resort the indices from the hierarchical clustering manually to keep the target sequence in the first row. This manual sorting is done in such a way as to keep the tree structure valid. The Hamming distances between the reordered sequences (see Suppl. Fig. 13e) show a block-like structure quite clearly after the reordering.

The attention pattern in the first layer of the network in the first recycling iteration (e.g. layer 0, head 7 in Suppl. Fig. 13a) is not very informative and is largely averaging as can be seen by looking at the range of the attention weights.

In the same head, but in the last recycling iteration (Suppl. Fig. 13b) we see that all sequences at all positions attend to the first row. Therefore this head behaves differently upon recycling and is presumably important for distribution the information in the recycled first row to the rest of the MSA representation.

Layer 6, head 6 (Suppl. Fig. 13c) shows a pattern that is fairly common in the column-wise MSA attention, here the pattern only varies lightly as one goes along the sequence and there is a clear structure in blocks of sequences that attend to each other. We note that these seem somewhat similar to the blocks derived from

hierarchical clustering using Hamming distance.

Whether attention patterns provide a good explanation for the behaviour of a given model or are predictive of interventions into the model is a topic of debate in the community, see [127], [128], [129]. A detailed analysis of the generality and predictivity of these attention patterns is beyond the scope of this paper.

## 1.17 Additional results

We present median all-atom $\mathrm{RMSD}_{95}$ on the CASP14 set in Suppl. Fig. 14. Figure 1 of the main paper shows the corresponding results on just the $\mathrm{C}\alpha$ atoms ($\mathrm{RMSD}_{95}$-$\mathrm{C}\alpha$).

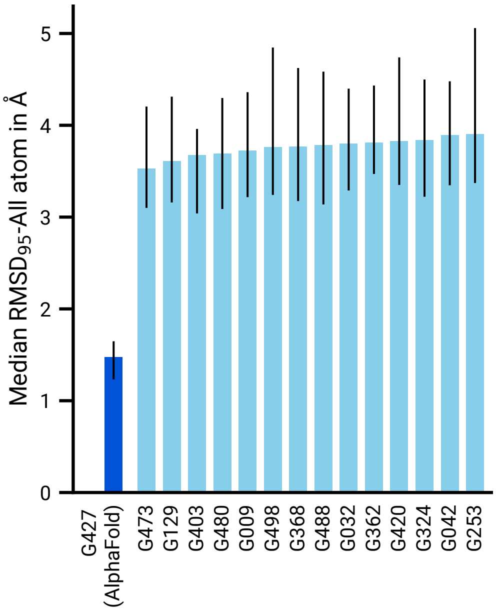


**Supplementary Figure 14** | Median all-atom $\mathrm{RMSD}_{95}$ on the CASP14 set of protein domains ($N = 87$ protein domains) relative to the top-15 entries (out of 146), group numbers correspond to the numbers assigned to entrants by CASP; error bars represent the 95% confidence interval of the median, estimated with 10,000 bootstrap samples.

In addition to the main Figure 2a, we show all-atom RMSD and RMSD for 100% coverage in Suppl. Fig. 15. We also report the quartiles of the distributions in Table 6. For this analysis, the recent PDB set of 10795 chains described in Methods was further filtered to exclude proteins with a template (identified by hmmsearch) from the training set with more than 40% sequence identity covering more than 1% of the chain.

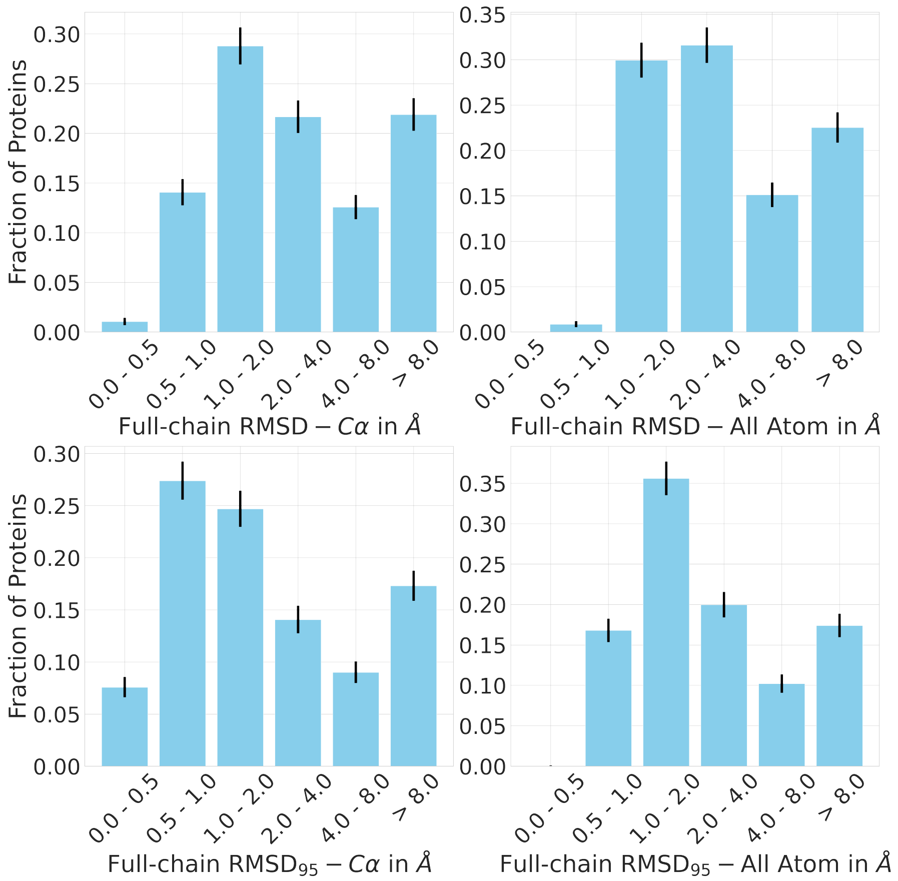


**Supplementary Figure 15** | Histograms of all-atom and backbone RMSD at 95% and 100% coverage for full-chain predictions on the template-reduced set ($N = 3144$ protein chains). Error bars are 95% confidence intervals (Poisson).

**Table 6** | Quartiles of distributions of all-atom and backbone RMSD at 95% and 100% coverage for full-chain predictions on the template-reduced set ($N = 3144$ protein chains).

| Quantity (in Å) | lower quart. | median | upper quart. |
|---|---|---|---|
| RMSD - C$\alpha$ | 1.31 | 2.32 | 6.49 |
| RMSD - All Atom. | 1.80 | 2.80 | 6.78 |
| $\mathrm{RMSD}_{95}$ - C$\alpha$ | 0.79 | 1.46 | 4.33 |
| $\mathrm{RMSD}_{95}$ - All Atom | 1.17 | 1.89 | 4.72 |

## References

[85] Jimmy Ba, Jamie R Kiros, and Geoffrey E Hinton. Layer normalization. *arXiv preprint arXiv:1607.06450*, 2016.

[86] L Steven Johnson, Sean R Eddy, and Elon Portugaly. Hidden markov model speed heuristic and iterative hmm search procedure. *BMC Bioinformatics*, 11(1):1–8, 2010.

[87] Michael Remmert, Andreas Biegert, Andreas Hauser, and Johannes Söding. Hhblits: lightning-fast iterative protein sequence searching by hmm-hmm alignment. *Nature Methods*, 9(2):173–175, 2012.

[88] Alex L Mitchell, Alexandre Almeida, Martin Beracochea, Miguel Boland, Josephine Burgin, Guy Cochrane, Michael R Crusoe, Varsha Kale, Simon C Potter, Lorna J Richardson, Ekaterina Sakharova, Maxim Scheremetjew, Anton Korobeynikov, Alex Shlemov, Olga Kunyavskaya, Alla Lapidus, and Robert D Finn. MGnify: the microbiome analysis resource in 2020. *Nucleic Acids Research*, 48(D1):D570–D578, 11 2019.

[89] Baris E Suzek, Yuqi Wang, Hongzhan Huang, Peter B McGarvey, Cathy H Wu, and UniProt Consortium. Uniref clusters: a comprehensive and scalable alternative for improving sequence similarity searches. *Bioinformatics*, 31(6):926–932, 2015.

[90] Milot Mirdita, Lars von den Driesch, Clovis Galiez, Maria J Martin, Johannes Söding, and Martin Steinegger. Uniclust databases of clustered and deeply annotated protein sequences and alignments. *Nucleic Acids Research*, 45(D1):D170–D176, 2017.

[91] Martin Steinegger, Markus Meier, Milot Mirdita, Harald Vöhringer, Stephan J Haunsberger, and Johannes Söding. Hh-suite3 for fast remote homology detection and deep protein annotation. *BMC Bioinformatics*, 20(1):1–15, 2019.

[92] Timo Lassmann, Oliver Frings, and Erik L L Sonnhammer. Kalign2: high-performance multiple alignment of protein and nucleotide sequences allowing external features. *Nucleic Acids Research*, 37:858–865, 2009.

[93] Martin Steinegger and Johannes Söding. Clustering huge protein sequence sets in linear time. *Nature Communications*, 9(1):1–8, 2018.

[94] Qizhe Xie, Minh-Thang Luong, Eduard Hovy, and Quoc V Le. Self-training with noisy student improves imagenet classification. In *Proceedings of the IEEE/CVF Conference on Computer Vision and Pattern Recognition*, pages 10687–10698, 2020.

[95] Ashish Vaswani, Noam Shazeer, Niki Parmar, Jakob Uszkoreit, Llion Jones, Aidan N Gomez, Lukasz Kaiser, and Illia Polosukhin. Attention is all you need. In *31st Conference on Neural Information Processing Systems (NeurIPS 2017)*, pages 6000–6010, 2017.

[96] Zihang Dai, Zhilin Yang, Yiming Yang, Jaime Carbonell, Quoc Le, and Ruslan Salakhutdinov. Transformer-XL: Attentive language models beyond a fixed-length context. In *Proceedings of the 57th Annual Meeting of the Association for Computational Linguistics*, pages 2978–2988, Florence, Italy, July 2019. Association for Computational Linguistics.

[97] Nitish Srivastava, Geoffrey Hinton, Alex Krizhevsky, Ilya Sutskever, and Ruslan Salakhutdinov. Dropout: a simple way to prevent neural networks from overfitting. *The Journal of Machine Learning Research*, 15(1):1929–1958, 2014.

[98] RA Engh and R Huber. Structure quality and target parameters. In *International Tables for Crystallography, Vol. F.* John Wiley & Sons, Ltd, 2006.

[99] Valerio Mariani, Marco Biasini, Alessandro Barbato, and Torsten Schwede. lddt: A local superposition-free score for comparing protein structures and models using distance difference tests. *Bioinformatics*, 29(21):2722–2728, 2013.

[100] Viktor Hornak, Robert Abel, Asim Okur, Bentley Strockbine, Adrian Roitberg, and Carlos Simmerling. Comparison of multiple amber force fields and development of improved protein backbone parameters. *Proteins: Structure, Function, and Bioinformatics*, 65(3):712–725, 2006.

[101] Peter Eastman, Jason Swails, John D Chodera, Robert T McGibbon, Yutong Zhao, Kyle A Beauchamp, Lee-Ping Wang, Andrew C Simmonett, Matthew P Harrigan, Chaya D Stern, Rafal P Wiewiora, Bernard R Brooks, and Vijay S Pande. Openmm 7: Rapid development of high performance algorithms for molecular dynamics. *PLOS Computational Biology*, 13(7):1–17, 07 2017.

[102] Mohammed AlQuraishi. End-to-End Differentiable Learning of Protein Structure. *Cell Systems*, 8(4):292–301.e3, 24 April 2019.

[103] Yang Zhang and Jeffrey Skolnick. Scoring function for automated assessment of protein structure template quality. *Proteins: Structure, Function, and Bioinformatics*, 57(4):702–710, 2004.

[104] Yang Zhang and Jeffrey Skolnick. TM-align: a protein structure alignment algorithm based on the TM-score. *Nucleic acids research*, 33(7):2302–2309, 2005.

[105] Jacob Devlin, Ming-Wei Chang, Kenton Lee, and Kristina Toutanova. Bert: Pre-training of deep bidirectional transformers for language understanding. *arXiv preprint arXiv:1810.04805*, 2018.

[106] Diederik P Kingma and Jimmy Ba. Adam: A method for stochastic optimization. In *Proceedings of the International Conference on Learning Representations*, 2015.

[107] Razvan Pascanu, Tomas Mikolov, and Yoshua Bengio. On the difficulty of training recurrent neural networks. In *Proceedings of the International Conference on Machine Learning*, pages 1310–1318, 2013.

[108] Y. LeCun, L. Bottou, G. Orr, and K. Muller. Efficient backprop. In *Neural Networks: Tricks of the Trade*. Springer, 1998.

[109] Kaiming He, Xiangyu Zhang, Shaoqing Ren, and Jian Sun. Delving deep into rectifiers: Surpassing human-level performance on imagenet classification. In *Proceedings of the IEEE Conference on Computer Vision*, pages 1026–1034, 2015.

[110] Xavier Glorot and Yoshua Bengio. Understanding the difficulty of training deep feedforward neural networks. In *Proceedings of the Thirteenth International Conference on Artificial Intelligence and Statistics*, pages 249–256, 2010.

[111] Priya Goyal, Piotr Dollár, Ross Girshick, Pieter Noordhuis, Lukasz Wesolowski, Aapo Kyrola, Andrew Tulloch, Yangqing Jia, and Kaiming He. Accurate, large minibatch sgd: Training imagenet in 1 hour. *arXiv preprint arXiv:1706.02677*, 2017.

[112] Boris T Polyak and Anatoli B Juditsky. Acceleration of stochastic approximation by averaging. *SIAM Journal on Control and Optimization*, 30(4):838–855, 1992.

[113] Jürgen Haas, Alessandro Barbato, Dario Behringer, Gabriel Studer, Steven Roth, Martino Bertoni, Khaled Mostaguir, Rafal Gumienny, and Torsten Schwede. Continuous automated model evaluation (cameo) complementing the critical assessment of structure prediction in casp12. *Proteins: Structure, Function, and Bioinformatics*, 86:387–398, 2018.

[114] Andreas Griewank and Andrea Walther. Algorithm 799: revolve: an implementation of checkpointing for the reverse or adjoint mode of computational differentiation. *ACM Transactions on Mathematical Software (TOMS)*, 26(1):19–45, 2000.

[115] Tianqi Chen, Bing Xu, Chiyuan Zhang, and Carlos Guestrin. Training deep nets with sublinear memory cost. *arXiv preprint arXiv:1604.06174*, 2016.

[116] Nikita Kitaev, Łukasz Kaiser, and Anselm Levskaya. Reformer: The efficient transformer. In *Proceedings of the International Conference on Learning Representations*, 2020.

[117] John Jumper, Richard Evans, Alexander Pritzel, Tim Green, Michael Figurnov, Kathryn Tunyasuvunakool, Olaf Ronneberger, Russ Bates, Augustin Žídek, Alex Bridgland, Clemens Meyer, Simon A A Kohl, Anna Potapenko, Andrew J Ballard, Andrew Cowie, Bernardino Romera-Paredes, Stanislav Nikolov, Rishub Jain, Jonas Adler, Trevor Back, Stig Petersen, David Reiman, Martin Steinegger, Michalina Pacholska, David Silver, Oriol Vinyals, Andrew W Senior, Koray Kavukcuoglu, Pushmeet Kohli, and Demis Hassabis. High accuracy protein structure prediction using deep learning. In *Fourteenth Critical Assessment of Techniques for Protein Structure Prediction (Abstract Book)*, pages 22–24, 2020.

[118] Jianyi Yang, Ivan Anishchenko, Hahnbeom Park, Zhenling Peng, Sergey Ovchinnikov, and David Baker. Improved protein structure prediction using predicted interresidue orientations. *Proceedings of the National Academy of Sciences of the United States of America*, 117(3):1496–1503, 21 January 2020.

[119] Zilong Huang, Xinggang Wang, Lichao Huang, Chang Huang, Yunchao Wei, and Wenyu Liu. Ccnet: Criss-cross attention for semantic segmentation. In *Proceedings of the IEEE/CVF International Conference on Computer Vision*, pages 603–612, 2019.

[120] Adam Zemla. LGA – a method for finding 3d similarities in protein structures. *Nucleic Acids Research*, 31:3370–4, 08 2003.

[121] Lisa Kinch, Andriy Kryshtafovych, and Nick Grishin. Target classification in the 14th round of the critical assessment of protein structure prediction (CASP14). https://predictioncenter.org/casp14/doc/presentations/2020_11_30_TargetClassification_Kinch_Updated.pdf, 2020. [Online; accessed 08-June-2021].

[122] David M. Kern, Ben Sorum, Sonali S. Mali, Christopher M. Hoel, Savitha Sridharan, Jonathan P. Remis, Daniel B. Toso, Abhay Kotecha, Diana M. Bautista, and Stephen G. Brohawn. Cryo-EM structure of the SARS-CoV-2 3a ion channel in lipid nanodiscs. *bioRxiv preprint bioRxiv:10.1101/2020.06.17.156554v3*, 2021.

[123] Jose M Dana, Aleksandras Gutmanas, Nidhi Tyagi, Guoying Qi, Claire O'Donovan, Maria Martin, and Sameer Velankar. SIFTS: updated structure integration with function, taxonomy and sequences resource allows 40-fold increase in coverage of structure-based annotations for proteins. *Nucleic acids research*, 47(D1):D482–D489, 2019.

[124] Jaina Mistry, Sara Chuguransky, Lowri Williams, Matloob Qureshi, Gustavo A Salazar, Erik LL Sonnhammer, Silvio CE Tosatto, Lisanna Paladin, Shriya Raj, Lorna J Richardson, et al. Pfam: The protein families database in 2021. *Nucleic Acids Research*, 49(D1):D412–D419, 2021.

[125] Antonina Andreeva, Eugene Kulesha, Julian Gough, and Alexey G Murzin. The SCOP database in 2020: expanded classification of representative family and superfamily domains of known protein structures. *Nucleic acids research*, 48(D1):D376–D382, 2020.

[126] Ilya N Shindyalov and Philip E Bourne. Protein structure alignment by incremental combinatorial extension (CE) of the optimal path. *Protein engineering*, 11(9):739–747, 1998.

[127] Sarthak Jain and Byron C Wallace. Attention is not Explanation. In *Proceedings of the 2019 Conference of the North American Chapter of the Association for Computational Linguistics: Human Language Technologies, Volume 1 (Long and Short Papers)*, pages 3543–3556, Minneapolis, Minnesota, June 2019. Association for Computational Linguistics.

[128] Sofia Serrano and Noah A Smith. Is attention interpretable? In *Proceedings of the 57th Annual Meeting of the Association for Computational Linguistics*, pages 2931–2951, Florence, Italy, July 2019. Association for Computational Linguistics.

[129] Sarah Wiegreffe and Yuval Pinter. Attention is not not explanation. In *Proceedings of the 2019 Conference on Empirical Methods in Natural Language Processing and the 9th International Joint Conference on Natural Language Processing (EMNLP-IJCNLP)*, pages 11–20, Hong Kong, China, November 2019. Association for Computational Linguistics.
