# Supplementary material for: Highly accurate protein structure prediction with AlphaFold
Source: Nature. 2021 Jul 15;596(7873):583–9. doi: 10.1038/s41586-021-03819-2 (PMC8371605; doi:10.1038/s41586-021-03819-2)
Supplement: Supplementary file 2 — Reporting Summary [file 41586_2021_3819_MOESM2_ESM.pdf]

nature research

Corresponding author(s): John Jumper, Demis Hassabis

Last updated by author(s): Jul 11, 2021

Reporting Summary

Nature Research wishes to improve the reproducibility of the work that we publish. This form provides structure for consistency and transparency in reporting. For further information on Nature Research policies, see our Editorial Policies and the Editorial Policy Checklist.

Statistics

For all statistical analyses, confirm that the following items are present in the figure legend, table legend, main text, or Methods section.

n/a	Confirmed	
☐	☒	The exact sample size (*n*) for each experimental group/condition, given as a discrete number and unit of measurement
☒	☐	A statement on whether measurements were taken from distinct samples or whether the same sample was measured repeatedly
☐	☒	The statistical test(s) used AND whether they are one- or two-sided<br>*Only common tests should be described solely by name; describe more complex techniques in the Methods section.*
☒	☐	A description of all covariates tested
☐	☒	A description of any assumptions or corrections, such as tests of normality and adjustment for multiple comparisons
☐	☒	A full description of the statistical parameters including central tendency (e.g. means) or other basic estimates (e.g. regression coefficient) AND variation (e.g. standard deviation) or associated estimates of uncertainty (e.g. confidence intervals)
☒	☐	For null hypothesis testing, the test statistic (e.g. *F*, *t*, *r*) with confidence intervals, effect sizes, degrees of freedom and *P* value noted<br>*Give P values as exact values whenever suitable.*
☐	☒	For Bayesian analysis, information on the choice of priors and Markov chain Monte Carlo settings
☒	☐	For hierarchical and complex designs, identification of the appropriate level for tests and full reporting of outcomes
☐	☒	Estimates of effect sizes (e.g. Cohen's *d*, Pearson's *r*), indicating how they were calculated

Our web collection on statistics for biologists contains articles on many of the points above.

Software and code

Policy information about availability of computer code

Data collection	Source code for the AlphaFold model, trained weights, and inference script will be made available under an open-source license at https://github.com/deepmind/ upon publication.<br><br>Neural networks were developed with TensorFlow v1 (https://github.com/tensorflow/tensorflow), Sonnet v1 (https://github.com/deepmind/sonnet), JAX v0.1.69 (https://github.com/google/jax/), and Haiku v0.0.4 (https://github.com/deepmind/dm-haiku). The XLA compiler is bundled with JAX and does not have a separate version number.<br><br>For MSA search on BFD+Uniclust30, and for template search against PDB70, we used HHBlits and HHSearch from hh-suite v3.0-beta.3 14/07/2017 (https://github.com/soedinglab/hh-suite). For MSA search on UniRef90 and clustered MGnify, we used jackhmmer from HMMER v3.3 (http://eddylab.org/software/hmmer/). For constrained relaxation of structures, we used OpenMM v7.3.1 (https://github.com/openmm/openmm) with the Amber99sb force field.<br><br>Construction of BFD used MMseqs2 version 925AF (https://github.com/soedinglab/MMseqs2) and FAMSA v1.2.5 (https://github.com/refresh-bio/FAMSA).
Data analysis	Data analysis used Python v3.6 (https://www.python.org/), NumPy v1.16.4 (https://github.com/numpy/numpy), SciPy v1.2.1 (https://www.scipy.org/), seaborn v0.11.1 (https://github.com/mwaskom/seaborn), Matplotlib v3.3.4 (https://github.com/matplotlib/matplotlib), bokeh v1.4.0 (https://github.com/bokeh/bokeh), pandas v1.1.5 (https://github.com/pandas-dev/pandas), plotnine v0.8.0 (https://github.com/has2k1/plotnine), statsmodels v0.12.2 (https://github.com/statsmodels/statsmodels) and Colab (https://research.google.com/colaboratory). TM-align v20190822 (https://zhanglab.dcmb.med.umich.edu/TM-align/) was used for computing TM-scores. Structure visualizations were created in Pymol v2.3.0 (https://github.com/schrodinger/pymol-open-source).

For manuscripts utilizing custom algorithms or software that are central to the research but not yet described in published literature, software must be made available to editors and reviewers. We strongly encourage code deposition in a community repository (e.g. GitHub). See the Nature Research guidelines for submitting code & software for further information.

Data

Policy information about availability of data

All manuscripts must include a data availability statement. This statement should provide the following information, where applicable:

- Accession codes, unique identifiers, or web links for publicly available datasets
- A list of figures that have associated raw data
- A description of any restrictions on data availability

All input data are freely available from public sources.

Structures from the PDB were used for training and as templates (https://www.wwpdb.org/ftp/pdb-ftp-sites; for the associated sequence data and 40% sequence clustering see also https://ftp.wwpdb.org/pub/pdb/derived_data/ and https://cdn.rcsb.org/resources/sequence/clusters/bc-40.out). Training used a version of the PDB downloaded 28/08/2019, while CASP14 template search used a version downloaded 14/05/2020. Template search also used the PDB70 database, downloaded 13/05/2020 (https://wwwuser.gwdg.de/~compbiol/data/hhsuite/databases/hhsuite_dbs/).

We show experimental structures from the PDB with accessions 6Y4F77, 6YJ178, 6VR479, 6SK080, 6FES81, 6W6W82, 6T1Z83, and 7JTL84.

For MSA lookup at both training and prediction time, we used UniRef90 v2020_01 (https://ftp.ebi.ac.uk/pub/databases/uniprot/previous_releases/release-2020_01/uniref/), BFD (https://bfd.mmseqs.com), Uniclust30 v2018_08 (https://wwwuser.gwdg.de/~compbiol/uniclust/2018_08/), and MGnify clusters v2018_12 (https://ftp.ebi.ac.uk/pub/databases/metagenomics/peptide_database/2018_12/).
Uniclust30 v2018_08 was further used as input for constructing a distillation structure dataset.

Field-specific reporting

Please select the one below that is the best fit for your research. If you are not sure, read the appropriate sections before making your selection.

☒ Life sciences ☐ Behavioural & social sciences ☐ Ecological, evolutionary & environmental sciences

For a reference copy of the document with all sections, see nature.com/documents/nr-reporting-summary-flat.pdf

Life sciences study design

All studies must disclose on these points even when the disclosure is negative.

Sample size	No sample size was chosen; the method was evaluated on the full CASP14 benchmark set, and all PDB chains not in the training set (subject to the exclusions noted below).
Data exclusions	The recent PDB set was filtered (see Methods for full details). Briefly this excludes chains with too few resolved residues, longer than 1400 residues, solved by NMR or with unknown/ambiguous residues. This set was also redundancy reduced (by taking representatives from a sequence clustering), and for some figures a sequence similarity-based filter was applied to remove entries too similar to the training set (see Methods and figure legends for details).
Replication	Not applicable, no experimental work is described in this study. The results are the output of a computational method which will be made available.
Randomization	Not applicable, we are not making a comparison between two groups
Blinding	Not applicable, we are not making a comparison between two groups

Reporting for specific materials, systems and methods

We require information from authors about some types of materials, experimental systems and methods used in many studies. Here, indicate whether each material, system or method listed is relevant to your study. If you are not sure if a list item applies to your research, read the appropriate section before selecting a response.

Materials & experimental systems

n/a	Involved in the study	
☒	☐	Antibodies
☒	☐	Eukaryotic cell lines
☒	☐	Palaeontology and archaeology
☒	☐	Animals and other organisms
☒	☐	Human research participants
☒	☐	Clinical data
☒	☐	Dual use research of concern

Methods

n/a	Involved in the study	
☒	☐	ChIP-seq
☒	☐	Flow cytometry
☒	☐	MRI-based neuroimaging
